# Exploring implementation strategies in evidence-based open streets programs for promoting physical activity in the Americas: a scoping review

**DOI:** 10.3389/fpubh.2025.1667559

**Published:** 2025-12-01

**Authors:** Raúl D. Gierbolini-Rivera, Milena Franco Silva, Lorna Fabiola Pineda, Bryan J. Weiner, Byron J. Powell, Ross C. Brownson, Diana C. Parra

**Affiliations:** 1 Prevention Research Center, School of Public Health, Washington University in St. Louis, St. Louis, MO, United States; 2 Brown School, Washington University in St. Louis, St. Louis, MO, United States; 3 Department of Health Systems and Population Health, University of Washington School of Public Health, Seattle, WA, United States; 4 Center for Dissemination and Implementation in the Institute for Public Health, Washington University in St. Louis, St. Louis, MO, United States; 5 Alvin J. Siteman Cancer Center and Division of Public Health Sciences, Department of Surgery, Washington University School of Medicine, St. Louis, MO, United States

**Keywords:** physical activity, ERIC strategies, health promotion, implementation science, scoping review

## Abstract

**Introduction:**

Chronic diseases are a leading public health concern in the Americas, and physical inactivity contributes significantly to their burden. Open streets program—community initiatives that temporarily close urban streets to vehicles—promote physical activity and community engagement, demonstrating positive health and social impacts. Effective implementation depends on identifying suitable strategies and frameworks. The Expert Recommendations for Implementing Change (ERIC) taxonomy, developed for clinical/healthcare contexts, has not been widely assessed for community-based interventions such as Open Streets. Implementation strategies could lead to specific outcomes (e.g., adoption, sustainability) that differ from program outcomes (e.g., PA levels, chronic disease prevalence). This scoping review focuses on the strategies that influence implementation outcomes. The primary aims of this review were to (1) identify implementation strategies for Open Streets programs and (2) identify opportunities for Open Streets programs to promote chronic disease prevention and physical activity, specifically in the Americas.

**Methods:**

A scoping review was conducted using Joanna Briggs Institute methodology and PRISMA-ScR guidelines. Six databases (PubMed, Scopus, Scielo, Web of Science, TRIS, and LILACS) were searched for studies on Open Streets programs in the Americas (January 2004–April 2024). Three reviewers independently screened studies in Rayyan. Strategies were extracted and coded according to the 73 ERIC taxonomy strategies. Quality appraisal used MMAT for empirical studies, AMSTAR-2 for reviews, and AACODS for gray literature.

**Results:**

Fifty-nine studies met the inclusion criteria, yielding 63 distinct implementation strategies for Open Streets programs. All 63 strategies were classified within the ERIC taxonomy. Frequently aligned ERIC strategies included building coalitions, capturing local knowledge, conducting needs assessments, and fostering stakeholder engagement. Open Streets strategies emphasized multisectoral collaboration, cultural adaptation, equity, and sustainability.

**Discussion:**

Among the 63 identified Open Streets strategies, many aligned with ERIC, providing a foundation in stakeholder engagement, coalition building, and flexible, context-sensitive implementation. However, several ERIC strategies were not relevant to Open Streets, underscoring that while many ERIC strategies were applicable, not all suited this setting. Open Streets programs may require supplemental approaches to address equity, cultural competence, and multisectoral collaboration. Findings present opportunities to tailor, test, and scale strategies that maximize the population health impact of Open Streets and similar community-based programs.

## 1 Introduction

Chronic diseases are a significant public health concern, responsible for approximately 81% of all deaths in the Americas region, equivalent to 5.8 million deaths annually (1, 2). The age-standardized mortality rate is 411.5 per 100,000 population, with variation between countries, ranging from 301.5 in Canada to 838.7 in Haiti (1, 2). The leading causes of death are cardiovascular disease (34.8%), cancer (23.4%), chronic respiratory diseases (9.2%), and diabetes (4.9%) (1–3). Premature mortality is increasingly concerning, as over one-third of chronic disease deaths occur in individuals under 70 years old, many of which are preventable (1). Modifiable risk factors such as tobacco use, unhealthy diets, obesity, and physical inactivity contribute to the chronic disease burden and are disproportionately distributed across the region (1). While some progress has been made in reducing mortality rates, current efforts remain insufficient to meet global health targets (2). Challenges, such as slow policy implementation, health system disruptions (e.g., the COVID-19 pandemic), and inequitable access to diagnosis and treatment, continue to exacerbate the chronic disease crisis in the Americas (1, 3). As inequities persist, it is crucial to emphasize the importance of enhancing both the uptake and effectiveness of evidence-based chronic disease prevention interventions in Latin America, given that many chronic disease deaths are preventable through such programs (4–6).

The current body of literature has demonstrated that engaging in physical activity (PA) is beneficial against numerous chronic diseases, including various cancers and premature mortality (7, 8). The WHO recommends that people aged 19–64 perform 150–300 min of moderate-intensity aerobic PA per week, or at least 75–150 min of vigorous-intensity aerobic PA per week. However, the global prevalence of physical inactivity has increased from 26.4% in 2010 to 31.3% in 2022, with Latin America and the Caribbean having the highest prevalence of physical inactivity among adults at 36.6% (9, 10). Various PA interventions have been introduced in Latin American cities to increase PA levels, including community-based initiatives such as Open Streets programs (11, 12).

These programs, known as Ciclovías Recreativas or Ciclopaseos in Spanish, Ruas de Lazer or Ciclofaixas de Lazer in Portuguese, and Open Streets programs in English —the term we will use in this paper— involve temporarily closing at least 1 km of city streets, transforming them into car-free zones for several hours a day (13–15). Such programs create safe and accessible areas for pedestrians, runners, skaters, and cyclists, encouraging leisure activities (15, 16) Additionally, community activities are organized alongside Open Streets to promote PA, foster civic engagement, stimulate local economic growth, support community development, revitalize public spaces, and advocate for walking and cycling as transportation alternatives (17, 18).

Since their inception in the 1960s, Open Streets programs have expanded to 400 locations globally (12), including Latin American cities such as Bogotá—the original site of implementation for the program Ciclovía (18)—as well as Quito, Santiago, São Paulo, and others. These programs have been instrumental in motivating urban residents to utilize public spaces, increase PA, and embrace active transportation (16). Furthermore, in addition to increasing PA among its participants, the programs have demonstrated multiple co-benefits, including increased social cohesion, reduced noise pollution, and improved air quality (15–18).

Open Streets programs offer positive health and economic benefits related to PA due to their low implementation costs and broad reach (15, 17). These programs are positive for health promotion and chronic disease prevention, particularly in areas with high rates of physical inactivity (16). However, increasing the speed and quality of evidence-based chronic disease prevention interventions in the Americas will require identifying effective implementation strategies. Implementation strategies are methods or techniques used to improve the adoption, implementation, sustainment, or scale-up of interventions, constituting the practical “how-to” aspect of transforming healthcare practices (19, 20). To address inconsistent language and insufficient descriptions of implementation strategies in the existing literature, the Expert Recommendations for Implementing Change (ERIC) taxonomy was developed to enhance clarity and consistency in defining these strategies (20, 21).

ERIC provides a comprehensive array of implementation strategies that can be tailored and applied to specific contexts and barriers (20). The effective implementation of evidence-based chronic disease prevention interventions depends partly on selecting and deploying strategies that address key implementation barriers. This matching depends on understanding how and why strategies work or fail, which means identifying the mechanisms through which they operate. A misalignment between strategies and barriers can often result in suboptimal implementation without identifying the mechanisms (20, 22, 23). Proctor et al. (19) proposed recommendations for naming, defining, and operationalizing strategies across seven dimensions: actors, actions, action targets, temporality, dose, outcomes affected, and justification for the strategy’s use (19). These guidelines promote the clear operationalization of these strategies, uncover potential implementation mechanisms through which they work, and may help surface strategies and mechanisms that are particularly relevant to low-resource settings. ERIC strategies are appropriate because they offer adaptable approaches that could help maximize limited resources, engage local stakeholders, and address contextual barriers to the implementation of health promotion interventions (24). For example, the ERIC strategy “build a coalition” could support health promotion in a low-resource setting by bringing together community leaders, local organizations, and public health practitioners to collaboratively plan and implement PA programs using shared resources (24).

Understanding implementation mechanisms, particularly those related to policy-based programs for PA, is crucial for low-resource settings domestically and internationally. This knowledge enables the development of the most efficient and resource-sensitive implementation approaches (25, 26). Foundational work is needed to document commonly used strategies and the putative mechanisms for implementing evidence-based PA programs in the Americas. We conducted a scoping review to better understand the opportunities that exist within widely used implementation science frameworks, such as ERIC and Open Streets programs, across the Americas. Implementation strategies could lead to specific outcomes (e.g., adoption, sustainability) that differ from program outcomes (e.g., PA levels, chronic disease prevalence). This scoping review focuses on the strategies that influence implementation outcomes. The primary aims of this review were to (1) identify implementation strategies across the literature on Open Streets programs and (2) identify opportunities where Open Streets strategies align or misalign with ERIC strategies, to promote chronic disease prevention and PA in the context of the Americas.

## 2 Methods

We conducted a scoping review on previously published research on Open Streets programs. We followed the Joanna Briggs Institute (JBI) methodology for scoping reviews and the PRISMA extension for Scoping Reviews (PRISMA-ScR) reporting (27, 28).

### 2.1 Eligibility criteria

Following JBI guidelines (28), we applied the Population, Concept, and Context framework for scoping reviews to define eligibility criteria:

- *Population:* We included studies with a population of any demographic or clinical background. No specific population characteristics were excluded.
- *Concept:* Studies focused on Open Streets programs were included. The outcomes measured included physical activity behavior, aerobic capacity, chronic disease outcomes, and the societal, economic, and environmental impacts of Open Streets programs. Exposure variables included health-promoting environmental (often in the built environment) factors, such as green spaces, walkability, access to public transportation, and health facilities.
- *Context:* We included only studies conducted in the Americas that focused on Open Streets programs.

### 2.2 Inclusion and exclusion criteria

As seen in Table 1, we included peer-reviewed journal articles published between 2004 and 2024 in English, Spanish, and Portuguese. Studies employing a variety of designs were considered, including quantitative empirical studies, qualitative studies, observational research, mixed-methods studies, and reviews (narrative, rapid, umbrella, scoping, systematic, and mixed-methods reviews). There were no restrictions on the population studied, and eligible studies were required to be conducted in community settings (e.g., schools or workplaces) but not in healthcare settings where interventions involved one-on-one advice or counseling. Studies with outcomes evaluating physical activity behavior, aerobic capacity, chronic disease outcomes, or the societal, economic, and environmental impacts of Open Streets or Ciclovia programs were included. Exposures included, but were not limited to, quantitative indicators of health-promoting environmental characteristics, such as green spaces, walkability, access to amenities, public transportation, or health facilities. We only included studies conducted in the Americas, and they had to provide sufficient details about the intervention, particularly concerning Open Streets or Ciclovia programs.

**Table 1:** Inclusion and exclusion criteria.

Criteria category	Inclusion criteria	Exclusion criteria
Document type	All peer-reviewed journal articles and gray literature (editorials, organizational reports)	Magazine articles, Books and book chapters, Book reviews, Poster and conference abstracts, Study protocols, Dissertations.
Study design	Quantitative empirical studies, qualitative studies, observational research, mixed-methods studies, and reviews (narrative, rapid, umbrella, scoping, systematic, and mixed-methods reviews).	No restriction on study design.
Timeframe	2004–2024	Any time before January 2004 and after May 2024
Language	English, Spanish, Portuguese	Other languages that are not English, Spanish, Portuguese
Population	Americas Region	Other regions that are not the Americas
Setting	Urban Streets	Clinical Settings
Exposure	Exposure included health-promoting environmental factors like green spaces, walkability, access to public transit, and health facilities.	Not focusing on health-promoting environmental factors.
Outcome	Outcomes included physical activity behavior, aerobic capacity, chronic disease outcomes, societal, economic, and environmental impacts of Open Streets programs.	Not focusing on outcomes such as physical activity behavior, aerobic capacity, chronic disease outcomes, societal, economic, and environmental impacts of Open Streets programs.

Studies were excluded if they were magazine articles, books, book chapters, book reviews, posters, conference abstracts, study protocols, or dissertations. Studies conducted in exercise laboratories, clinical or hospital settings, or those that used physical activity as a therapeutic intervention or for rehabilitation were also excluded. Additionally, studies that did not specifically mention Ciclovia Recreativa, Open Streets, or similar programs (e.g., Play Streets) or were conducted outside the Americas were excluded.

### 2.3 Search strategy

The search strategy was conducted in May 2024 with the assistance of a librarian, and it included articles published from January 2004 to May 2024, spanning the last 20 years, to capture recent program developments. Six electronic databases (PubMed, Web of Science, Scopus, Scielo, Lilacs, and Transportation Information Services (TRIS)) were systematically searched. PubMed, Scopus, Web of Science, and TRIS were searched in Spanish and Portuguese with translations of the keywords. Scielo and Lilacs were searched using English and Spanish keywords. We selected keywords based on five types of search terms, including “physical activity”, “chronic disease”, “built environment”, “policy” and “Open streets” terms. The search incorporated combinations of these keywords, outlined in Table 2 (for the complete search strategy, see Supplementary Table 1).

**Table 2:** Search strategy.

Topics	Physical activity	Chronic disease	Built environment	Policy	Open streets
Keywords	Exercise	Chronic disease	Built environment	Intervention	Open streets
	Physical activity	Neoplasms	Environmental design	Community intervention	Ciclovia
	Physical fitness	Prevention	Urban design	Program	Recreovia
	Cardiorespiratory fitness	Cancer		Policy	
	Walk*				
	Resistance training				
	Sport				
	Motor activit*				
	Sedentary				
	Leisure activities				
	Bicycl*				
	Active transportation				
	Non-motorized transportation				

Syntax in PubMed-Medline: “Exercise”[Mesh] OR (“physical activity”) OR (“physical fitness”) OR (“cardiorespiratory fitness”) OR (“aerobic capacity”) OR (walk*) OR (“resistance training”) OR (community) OR (“health promotion”) OR (sport) OR (“motor activity”) OR (sedentary) OR (inactivity) OR (“leisure activities”) OR (exercise) OR (“moderate activit*”) OR (“vigorous activit*”) OR (bicycl*) OR (bike) OR (“active transportation”) OR (“non-motorized transportation”) AND “Chronic Disease”[Mesh] OR “Neoplasms”[Mesh] OR “chronic disease” OR “neoplasms” OR “prevention” AND “Built Environment”[Mesh: NoExp] OR (“environmental design”) OR (“built environment”) OR (“urban design”) AND (“intervention”) OR (“community intervention”) OR (“program”) OR (“policy”) AND (“open streets”) OR (“ciclovia”) OR (“ciclovias”) OR (“recreovia”).

We adopted the three-step search strategy proposed by JBI. First, we performed an initial limited search in selected databases and analyzed the titles, abstracts, and index terms of retrieved papers to identify relevant keywords. In the second step, we conducted a comprehensive search across all databases using the identified keywords and index terms. The third and final step focused on references from studies that have been selected for full-text inclusion in the review.

### 2.4 Study selection

One reviewer conducted systematic database searches (RGR) and imported the results into Rayyan (29), a web-based system for screening blind literature reviews. Two independent reviewers (RGR, FP) removed duplicate entries and screened titles and abstracts against predefined inclusion and exclusion criteria to determine study eligibility. Three independent reviewers (RGR, MFS, FP) screened the full texts of potentially relevant articles and documented reasons for exclusion. To ensure inter-rater reliability, in both the title/abstract and full-text phases of the screening, each article was assessed independently and blinded. The three reviewers resolved disagreements during the screening through consensus meetings.

### 2.5 Data extraction, data analysis, and synthesis

Data extraction was performed independently by three of the authors (RGR, MFS, FP). Data extraction was completed using a template developed by the authors in Microsoft Excel. The extraction document included multiple fields divided into descriptive and result extraction fields. Descriptive extraction fields included study characteristics, strategies of Open Streets programs, and identified gaps and opportunities in the articles (see data extraction codebook in Supplementary Table 2). During the data extraction phase, we analyzed the settings in which the included articles were conducted, enabling us to assess the frequency of these articles by country within the Americas. To accomplish this, we compiled the frequency of articles by country and city and then developed a map using Microsoft Excel to represent the data visually. This was developed to identify the countries with the highest representation and to discern whether the articles were based in the Global North or the Global South.

Our extraction approach comprised five key steps. For step 1, three independent reviewers (RGR, MFS, FP) extracted potential implementation strategies from the studies included in the review. In step 2, each extracted Open Streets strategy was matched to the list of 73 ERIC strategies (19). Strategies were coded as “1” if they matched an ERIC strategy and “0” if they did not. This process was divided equally for two reviewers (RGR, MFS) and was reviewed by two additional reviewers (DP, FP) to ensure inter-rater reliability. This process enabled us to systematically assess which Open Streets strategies aligned with ERIC strategies.

In step 3, a deductive thematic analysis approach (30) was employed to identify themes, informed by the feasibility and importance of ERIC strategies in grouping the strategies (31). Color coding was used to organize the Open Streets strategies under each theme and subtheme (see Supplementary Table 3). This strategy enabled the data to be connected to nine themes and 52 sub-themes, allowing flexibility to facilitate the categorization process of the Open Streets strategies (see Supplementary Table 4). For step 4, we calculated the frequencies and percentages for the number of times Open Streets strategies matched the ERIC strategies. Using the frequencies and percentages, strategies were grouped under relevant themes and sub-themes. One reviewer (RGR) initially performed this categorization, and then it was reviewed by three additional team members (DP, MFS, FP) to ensure accuracy and consistency. The final step involved refining the list of Open Streets strategies. Team members independently reviewed each identified strategy to determine whether to include or exclude it, and a consensus meeting was conducted to finalize the list. At the end of the data extraction process, a narrative synthesis was prepared by three members (RGR, MFS, FP) of the research team, which included the frequencies of locations (countries and cities of study) and a narrative synthesis of the study characteristics.

### 2.6 Assessment of methodological quality

We appraised each study with a tool that matched its design: the MMAT (Mixed Methods Appraisal Tool) 2018 for empirical studies (ranging from qualitative, quantitative randomized controlled trials, quantitative non-randomized studies, quantitative descriptive studies, and mixed method studies), recording item-by-item judgments (“Yes/No/Cannot tell”) with brief evidence notes (32). For reviews/evidence syntheses, we applied the AMSTAR-2 (A Measurement Tool to Assess Systematic Reviews, version 2) tool to evaluate the methodological rigor recording, and item-by-item judgments were recorded (“Yes/No/Partial”) (33). For gray literature, technical manuals, and commentaries/conceptual papers, we used the AACODS checklist (Authority, Accuracy, Coverage, Objectivity, Date, Significance), and item-by-item judgments were recorded (“Yes/No/Partial”) (34). All appraisals were logged in an Excel template for MMAT, AMSTAR-2, and AACODS to ensure consistency and to contextualize findings in the synthesis. One member of the team (RGR) appraised all the articles, and two other members (DP and MFS) verified the appraisal for accuracy.

## 3 Results

**Figure 1:**
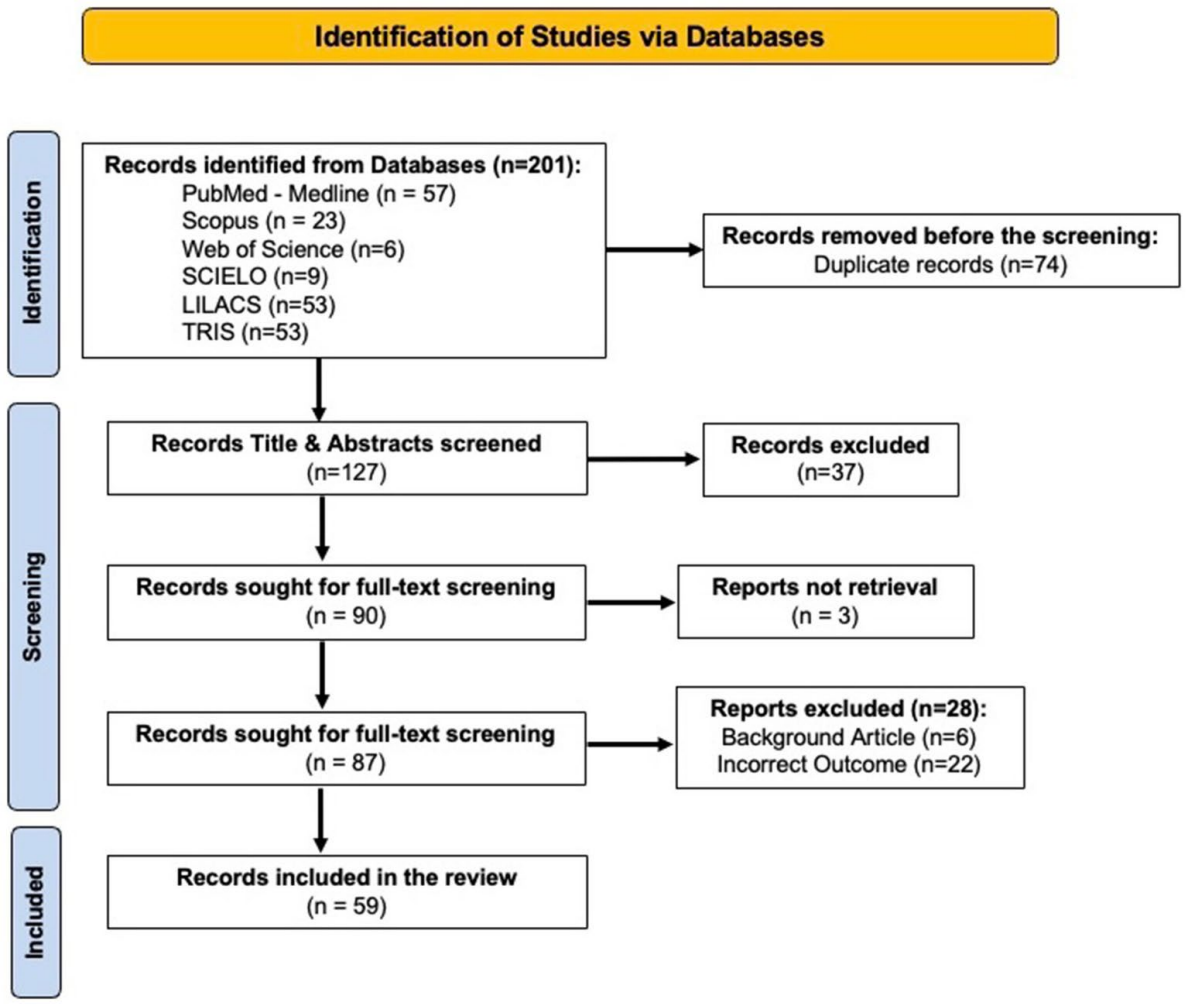
PRISMA Flowchart of the Included Studies.

Overall, we identified 201 articles, and after excluding 74 duplicates, 127 were screened for titles and abstracts. Full-text assessment was performed on a total of 87 studies, and after eligibility assessment, 59 studies were included in this review (see Figure 1). The main reasons for exclusion were incorrect outcome (*n* = 22) or background article (*n* = 6).

### 3.1 Narrative synthesis of study designs, settings, similarities, and differences of key findings

The 59 studies included in this review (see Table 3) were published between 2005 and 2024, spanning nearly two decades on Open Streets programs. Cross-sectional designs and observational methods were the most common, such as intercept surveys of participants (35) and systematic observation counts using SOPARC protocols (36, 37). Several studies incorporated accelerometry (38), GIS (39), or spatial analyses to assess equity in access to Ciclovia (40). Mixed-methods (18, 41) and qualitative (42–44) approaches highlighted program sustainability, community engagement, and policy processes, while participatory and CBPR (Community-Based Participatory Research) designs emphasized advocacy and local ownership as essential factors of Open Streets events (38, 45–47). Economic analysis and natural experiments quantified causal effects and cost-effectiveness, with cost–benefit ratios ranging from 1.02 to 4.26 across different cities (48), and substantial improvements in air quality during Open Streets events (49).

**Table 3:** Summary characteristics of included studies (*N* = 59).

Title/author/year	Study design	Study setting	Country	Key findings	Open street strategies	References
*Social Inclusion and Physical Activity in Ciclovía Recreativa Programs in Latin America* Mejia-Arbelaez et al. (2021)	Cross-sectional, secondary data analysis (harmonized surveys conducted 2015–2019; *N* = 3,282 participants).	Urban settings; comparison of four major cities (Bogotá, Mexico City, Santiago de Cali, Santiago de Chile). Focus on Ciclovía Recreativa routes and neighborhoods (urban segregation context)	Multi-country: Colombia (Bogotá, Santiago de Cali), Mexico (Mexico City), Chile (Santiago de Chile). Region: Latin America → Global South	Participants in Bogotá and Santiago de Cali showed the greatest mobility across neighborhoods of different socioeconomic status, while those in Mexico City and Santiago de Chile mostly stayed within similar areas. Physical activity levels did not differ by sex or SES, suggesting Ciclovía programs can foster social inclusion and provide equitable opportunities for physical activity.	Apply tactical urbanism to Open Street programs to promote mobility in times of health emergencies. (Page. 19, Discussion Section)	(17)
*Engaging citizen scientists to build healthy park environments in Colombia* Alejandra-Rubio et al. (2020)	Mixed-methods, explanatory sequential design (observational park assessments, accelerometry, surveys, participatory citizen science using “Our Voice” model).	Urban parks in Bogotá (Santa Isabel and San Andrés). Parks are in low- to middle-income neighborhoods	Colombia → Global South	The Recreovía program fostered physical activity, social cohesion, and empowerment in community parks. Citizen scientists identified enablers (e.g., inclusive facilities, outdoor equipment, social interaction) and barriers (e.g., cleanliness, safety, maintenance). The Our Voice process empowered residents to advocate for changes, leading to ripple effects including program continuation, expansion, and greater policy engagement.	Encouraging communication between different parties promotes engagement as a common goal of PA promotion. (Page 9, Discussion Section)	(38)
*Active streets for children: The case of the Bogota Ciclovia* Tiana et al. (2019)	Quantitative cross-sectional study (secondary data from ISCOLE and MARA studies; *N* = 923 children, 9–13 years). Measures included accelerometry for PA/SED and anthropometry for BMI.	Urban setting, Bogotá (Ciclovía program routes across diverse socioeconomic neighborhoods)	Colombia → Global South	Frequent Ciclovía users engaged in more MVPA and less sedentary time on Sundays compared with sporadic/non-users, though no differences were observed on weekdays. Participation was higher among low-to-middle SES children. However, frequent users also had higher BMI z-scores, suggesting Sunday-only activity may be insufficient for weight control.	Utilize family cohesion to motivate children to participate in Open Streets. (Page 9, Discussion Section)	(59)
Ciclovia in a Rural Latino Community: Results and Lessons Perry el al., (2017)	Quantitative descriptive, community-based participatory research (evaluation of a single Ciclovía using observation counts and intercept surveys).	Rural setting, agricultural town of ~10,000 residents, 74% Latino, 24% below poverty line	United States (Toppenish, Washington—rural Latino community) → Global North	The rural Ciclovía attracted ~200 participants per hour (≈2% of town population). Most adults and children achieved ≥30 min of physical activity, with 79% reporting they otherwise would have been indoors sedentary. Lessons highlight the potential of Ciclovías to provide safe, low-cost PA opportunities in underserved rural areas.	Motivate participants by promoting social interactions. (Page 5, Discussion Section)	(45)
*Social cohesion emerging from a community-based physical activity program: A temporal network analysis* María-Jaramilo et al. (2021)	Quantitative cross-sectional study; Social network analysis (Facebook data 2008–2016, *N* = 3,597 nodes/51,361 edges) + surveys of participants (*n* = 50). Longitudinal temporal network analysis	Quantitative cross-sectional study; Social network analysis (Facebook data 2008–2016, *N* = 3,597 nodes/51,361 edges) + surveys of participants (*n* = 50). Longitudinal temporal network analysis	Colombia (Bogota) → Global South	The Recreovía’s social networks showed increasing cohesion over time, with physical activity instructors as central hubs. Network growth followed a super-linear model (*β* = 1.73), indicating more ties than nodes, reflecting emergent social cohesion. Innovation periods (new classes, Facebook promotion) spurred growth. Surveys confirmed participants interacted beyond classes, highlighting Recreovía’s role in strengthening social capital and social cohesion.	PA instructors play an important role in PA promotion through social media. (Page 11, Discussion Section)	(78)
*Participation and engagement of a rural community in Ciclovía: progressing from research intervention to community adoption* Ko et al. (2021)	Community-based participatory research (CBPR); longitudinal descriptive evaluation of the same Ciclovía program over 3 years (2017–2019) using attendance counts, intercept surveys, and planning documentation.	Rural setting; small agricultural town with limited PA infrastructure.	United States (Toppenish, Washington—rural agricultural town, ~10,000 residents, 74% Hispanic/Latino, 24% below poverty line) → Global North	Attendance grew from 189/h in 2017 to 394/h in 2019, representing >4% of the town’s population. Engagement increased as the event transitioned from a research-led intervention to a community-adopted program. Women and children were the majority of participants, with notable growth among American Indian attendees. Community leadership, planning tools, and local resource mobilization were key for sustainability.	Active transportation can be socially transmitted and become a norm to promote PA in Open Street programs. (Page 8, Discussion Section)	(46)
*Geographic Distribution of the Ciclovia and Recreovia Programs by Neighborhood SES in Bogotá: How Unequal is the Geographic Access Assessed* Via *Distance-based Measures?* Parra et al. (2021)	Spatial epidemiological study using secondary GIS data (2015); Manhattan/network distances from block centroids to nearest Ciclovía access point or Recreovía site; SES stratification (1–6); Kruskal–Wallis tests for SES differences.	Urban; citywide analysis of Bogotá’s residential/mixed-use blocks using GIS to assess proximity to Ciclovía corridors and Recreovía sites	Colombia (Bogotá) → Global South	Marked inequities in geographic access to Ciclovía: median distance from SES 1 = ~2,938 m vs. SES 6 = ~482 m (six-fold difference); 71% of blocks are >1,000 m from Ciclovía access. Recreovía distances show less disparity overall (median ≈2,125 m), though SES differences remain. Findings argue for expanding routes/sites in low-SES areas to reduce inequities.	The program’s evaluation strategy involves studying the distance between Open Streets and socioeconomic status (SES) categorized areas. (Pages 105–106, Discussion Section)	(58)
*Open Streets Initiatives in the United States: Closed to Traffic, Open to Physical Activity* Kuhlberg et al. (2014)	Descriptive overview of U. S. Open Streets in 2011 using literature/Internet searches and organizer contacts; extracted frequency, route length, attendance, evaluation, and host-city sociodemographics.	Urban/suburban multi-city U. S. context; descriptive comparison of initiatives and host-city characteristics.	United States → Global North. (National overview of U. S. Open Streets)	Identified Open Streets in 47 U. S. cities (2011) with wide variation in route length (a few blocks to 51 miles) and frequency (annual to monthly); attendance reporting was sporadic, and few events conducted formal evaluations. Authors conclude initiatives are growing and have potential to promote PA, but stronger evaluation is needed.	_	(79)
The Ciclovia and Cicloruta Programs: Promising Interventions to Promote Physical Activity and Social Capital in Bogota´, Colombia Torres et al. (2013)	Two cross-sectional intercept surveys (October 2009): *n* = 1,000 Ciclovía participants; *n* = 1,000 Cicloruta users; comparative analysis of PA outcomes, safety, social capital (SC), and equity indicators.	Urban; citywide programs—Ciclovía (121 km of streets opened to people on Sundays/holidays) and Cicloruta (≈300 km bicycle path network).	Colombia (Bogotá) → Global South.	Most Ciclovía participants met leisure-time PA recommendations (59.5%), while most Cicloruta users met transport cycling recommendations (70.5%); Ciclovía participants reported higher safety perceptions (traffic and crime) and higher social capital (adjusted OR ≈ 2.0) than Cicloruta users; both programs served largely low- and middle-SES residents, highlighting potential for equitable PA promotion.	_	(62)
*Reclaiming the streets for people: Insights from Ciclovías Recreativas in Latin America* Sarmiento et al. (2017)	Mixed-methods, convergent parallel: online survey of program coordinators (2014–2015) + semi-structured interviews and policy/document review for five case studies; descriptive statistics and thematic analysis of sustainability/scaling-up factors.	Urban community programs (Ciclovías) analyzed at program level (survey of 67 programs) plus in-depth case studies (interviews, policy docs).	Multi-country: Latin America (programs across 7 LAC; five case studies in Mexico City, Cuautitlán Izcalli, Quito, Santiago de Chile, and Bogotá) → Global South	Latin American Ciclovías have expanded rapidly since 2000, are heterogeneous yet socially inclusive (most routes connect low–high income areas; minority participation common), frequently offer PA classes, and often promote bicycling. All five case studies met sustainability and scaling-up definitions, commonly featuring government support, alliances, champions, community appropriation, and funding stability, though political favorability and funding models varied. Overall, Ciclovías are flexible, scalable strategies to promote physical activity and equity in public space.	_	(18)
*Start Small, Dream Big: Experiences of Physical Activity in Public Spaces in Colombia.* Díaz del Castillo et al. (2017)	Mixed-methods (convergent): semi-structured interviews (May–Oct 2015), document review, and descriptive analysis of program history, characteristics, funding, capacity building, challenges; guided by sustainability frameworks (95, 96).	Urban public spaces (parks, trails, Ciclovías/streets, plazas; free PA classes offered in community settings)	Colombia → Global South (programs analyzed: Bogotá’s Recreovía and the national HEVS (Healthy Habits and Lifestyles Program) program)	Both programs have been sustained >10 years and benefited 1,455 communities. Sustainability/scale-up were enabled by flexibility/adaptability, investment in instructor training/working conditions, public funding with accountability, diversified resources, community support and multilevel champions, and ongoing policy advocacy.	Allow flexibility of the program to adjust to changes in budget and increase participation. (Page 45, Results Section 3.2.1) Encouraging capacity building increased the quality of the program. (Page 45, Results Section 3.2.2) Budget allocation and accountability improved and scaled up the programs. (Page 46, Results Section 3.2.3)	(41)
*The Recreovía of Bogotá, a Community-Based Physical Activity Program to Promote Physical Activity among Women: Baseline Results of the Natural Experiment Al Ritmo de las Comunidades* Sarmiento et al. (2017)	Baseline of a natural experiment using systematic observation (SOPARC) of park users; cross-sectional comparisons of parks with vs. without Recreovía; 2013 data; multilevel regression for park use and PA levels.	Urban public parks; nine parks (3 future-Recreovía, 3 control, 3 existing-Recreovía).	Colombia (Bogotá) → Global South.	Among 4,925 observed users across 702 observations, parks with Recreovía had more women and higher MVPA among women (75% vs. 61%; sedentary 25% vs. 39%) compared with parks without the program; the opposite pattern was observed for men. Recreovía appears to promote park use and PA among women on weekends.	_	(60)
*Social conditions and urban environment associated with participation in the Ciclovia program among adults from Cali, Colombia* Gómez et al. (2015)	Multilevel cross-sectional study (2011–2012) of adults 18–44 (*n* ≈ 729) using a four-stage probabilistic sampling design; face-to-face questionnaire + GIS indicators; multilevel logistic regression for participation in Ciclovía (yes/no).	Urban—neighborhood-level analysis in Cali using GIS measures linked to a household survey.	Colombia (Cali) → Global South.	Participation in the last four weekends was ~7%. Higher odds of participation for men and those with completed high school; positive association with living in neighborhoods with Ciclovía corridors; negative association with traffic fatalities in the neighborhood; middle-SES neighborhoods showed lower odds vs. low-SES.	_	(52)
*Differences between leisure-time physical activity, health-related quality of life and life satisfaction: Al Ritmo de las Comunidades, a natural experiment from Colombia* *Barradas et al. (2016)*	Cross-sectional baseline survey (2013) within a natural experiment; *N* = 1,533 adults (501 in Recreovía parks). Measures: IPAQ (leisure-time PA min/wk), EORTC QLQ-C30 (HRQoL), and Questions on Life Satisfaction.	Urban Bogotá: adults sampled around nine parks linked to the Recreovía natural experiment (parks with Recreovía, matched controls, and “future” sites).	Colombia (Bogotá) → Global South.	Higher leisure-time PA was associated with higher life satisfaction (p < 0.01); no significant differences in HRQoL by PA level. Recreovía participants reported higher HRQoL and LS (Life Satisfaction) than non-participants (both *p* < 0.001).	_	(80)
*The Ciclovía-Recreativa: A Mass-Recreational Program With Public Health Potential* Sarmiento et al. (2010)	Descriptive review/scoping of Ciclovía programs using a systematic search of peer-reviewed and gray literature, complemented by expert interviews/consultation.	Predominantly urban programs (~84% urban)	Multi-country review across the Americas and Caribbean (programs in 11 countries; many in Latin America → Global South emphasis).	Reviewed 38 programs: frequency typically 18–64 events/year, event duration 2–12 h, route length 1–121 km, and wide attendance (≈60 to 1,000,000 per event). About 71% included PA classes and 89% connected with parks. Authors conclude Ciclovías have strong public-health potential, but effectiveness evidence was limited at the time; they call for transnational evaluations.	_	(81)
*Innovative participatory evaluation methodologies to assess and sustain multilevel impacts of two community-based physical activity programs for women in Colombia* Alejandra-Rubio et al. (2022)	Participatory action evaluation using two methods: (1) Our Voice citizen science (geocoded photos + narratives; community meetings to prioritize solutions) and (2) Ripple Effects Mapping (group mind-mapping of outcomes); thematic analysis within a socioecological framework. Participants: stakeholders (*N* = 6) and program users (*N* = 34; Recreovía *n* = 16, My Body *n* = 18).	Urban; Bogotá programs Recreovía (free PA classes in public parks) and My Body (Recreovía-delivered program for breast cancer survivors); stakeholder meetings at university/community sites.	Colombia → Global South.	Infrastructure was the most salient facilitator and barrier to PA; safety and civic culture also mattered. Programs fostered social bonds, civic empowerment, and leadership, supporting sustainability and scale-up; dialogue with stakeholders led to adaptations and continued participation even during COVID-19.	A thorough evaluation of programs and identification of barriers, facilitators, and outcomes are key for sustainability. (Page 12, Discussion Section)	(47)
*Unintended impacts of the Open Streets program on noise complaints in New York City.* Benavides et al. (2023)	Quantitative ecological analysis: negative binomial mixed-effects models with splines; compares summer 2019 (pre) vs. summer 2021 (post) implementation; adjusts for SES, population/building density, time, and other covariates; multiple sensitivity analyses (alternative Open Streets dataset, POIs (Points of Interest), Open Restaurants, spatial correlation).	Urban; NYC census tracts (citywide), linking Open Streets coverage to daily 311 street/sidewalk and vehicle noise complaints.	United States (New York City) → Global North.	Higher proportion of Open Streets in a tract was nonlinearly associated with more street/sidewalk noise complaints; relative to the mean coverage (0.11%), 10% coverage → RR 1.21 (95% CI: 1.04–1.42); patterns robust to multiple sensitivity checks. Open Streets tended to be located in more affluent tracts, while noise complaints were higher in poorer tracts.	_	(82)
*Use of an Elevated Avenue for Leisure-Time Physical Activity by Adults from Downtown São Paulo, Brazil.* Quieroti-Rodrigues et al. (2022)	Cross-sectional survey (Dec 2017–Mar 2019); cluster sampling of adults ≥18 y within 1.5 km; *N* = 235 completed questionnaires. LTPA measured with IPAQ-long (electronic); barriers scale; bivariate tests and Poisson regression with post-stratification weights to estimate Prevalence Ratios, adjusted for sociodemographics, distance, and barriers.	Urban; the “Minhocão” elevated avenue (open to people at night and on weekends). Residents within ≤500 m vs. 501–1,500 m of access points were sampled.	Brazil (São Paulo) → Global South.	Users of the Minhocão had substantially higher odds of meeting ≥150 min/week LTPA than non-users (PR ≈ 2.19; 95% CI 1.66–2.90), and higher walking, moderate, and vigorous LTPA; proximity to access points was not associated with LTPA. Top barriers: safety in/around the site, rainy weather, lack of vegetation, and lack of facilities.	_	(53)
*Prevention of childhood obesity and food policies in Latin America: from research to practice.* Pérez-Escamilla et al. (2017)	Narrative policy review with case studies, coded using the Complex Adaptive Health Systems (CAS) framework to identify elements supporting successful implementation and sustainability.	Policy-level analysis across Latin America; includes a case study on Ciclovías (Open Streets) implemented in many Latin American cities.	Multi-country (Latin America) focus (case studies from Mexico, Chile, Ecuador, Argentina, and Ciclovías across Latin America) → Global South.	Effective and sustainable policy change relied on: evidence justifying policy, evidence-based civil-society advocacy, political will, and intersectoral negotiation/legislation; authors conclude that well-coordinated, intersectoral partnerships are needed to implement evidence-based anti-obesity policies.	Identify champions. (Page 37, Conclusion Section)	(72)
*Evaluation of Event Physical Activity Engagement at an Open Streets Initiative Within a Texas–Mexico Border Town* Salazar-Collier et al. (2018)	Quantitative observational evaluation: direct observation (SOPARC-adapted) of activity along the route and at activity hubs, plus intercept surveys capturing sociodemographics, event PA engagement, prior attendance; descriptive stats, χ^2^ tests, Wilcoxon tests, and logistic regression.	Urban open-streets events (“CycloBia”) on a 2–3 mile route connecting four parks; evaluations across four 2015 events using route counts and on-site intercept surveys.	United States (Brownsville, Texas) → Global North.	Cycling was the most observed activity (73.6%) followed by walking (22.9%); attendees reported a median 120 min of PA at the event, and 17.3% met weekly PA guidelines during the event. Predictors of meeting guidelines included past event attendance, sex, age, and Hispanic ethnicity; the events reached a predominantly low-income, Hispanic population.	_	(36)
*Move on Bikes Program: A Community-Based Physical Activity Strategy in Mexico City* Medina et al. (2019)	Cross-sectional evaluation: on-route intercept survey of participants (*n* = 679) plus program wide counts at 16 observation points; average speed by activity measured via video to classify intensity; descriptive stats and ordinal regression for attendance frequency correlates.	Urban open-streets program (“Muévete en Bici,” 55-km interconnected route; Sundays 8:00 a.m.–2:00 p.m.).	Mexico (Mexico City) → Global South.	On a typical program day, 21,812 people participated; users accumulated 221 min MVPA and 88.4% met ≥150 min/week during the event; 29.6% attended every Sunday. Frequent attendance was more likely among men, ages 41–64, those sufficiently/very active, those using active transport to reach the route, and those who came alone. Estimated program contribution ≈ + 71 min/week MVPA to >20,000 users.	Consider the length and frequency of the program, which can influence participation. (Page 10, Discussion Section)	(51)
*A “Ciclovia” in San Francisco: Characteristics and Physical Activity Behavior of Sunday Streets Participants* Zieff et al. (2014)	Cross-sectional intercept survey using a 36-item instrument; 639 recruited, 600 complete; analyses included descriptive statistics and group comparisons (first-time vs. multiple-event attendees) via MANOVAs; additional scales (reasons to attend/return, subjective vitality).	Urban open-streets events (Sunday Streets SF). Surveys administered at 3 events in 2010 across multiple neighborhoods/routes.	United States (San Francisco, California) → Global North.	Participants were generally active (≈79% reported PA 3–7 days/week) and roughly mirrored the city’s ethnic minority distribution; ~1 h of PA accrued during events. Multiple-event attendees reported more weekly PA bouts (≈4.22 vs. 3.69) and longer PA at the event (≈75 vs. 56 min) than first-timers; intentions to return and positive/safe experience ratings were higher among repeat attendees.	_	(83)
Target Population Involvement in Urban Ciclovias: A Preliminary Evaluation of St. Louis Open Streets Hipp et al. (2012)	Quantitative observational evaluation of four 2010 events: systematic counts/observations (*n* = 1,452 participants observed) and on-site intercept surveys (82 adults; ~65.6% response). Domains: PA, travel, sense of community, marketing, spending, demographics.	Urban open-streets events within the city; 2010 route spanned ~6 miles (April/June) and a ~ 7-mile loop (October).	United States (St. Louis, Missouri) → Global North	April event averaged ~587 participants/h/location; October ~211/h/location; most activity was cycling (adults 67.6%, youth 74.6%). >50% of surveyed adults met CDC weekly PA thresholds at the event; participants reported ~2.5 h at Open Streets. Attendees were primarily white, college-educated, and middle-income, not representative of the majority-minority city; authors recommend efforts to increase participation among low-income/minority residents. 82% spent money associated with the event and 56% discovered new businesses; 89% reported more positive feelings about the city and 91% felt very safe along the route.	Direct communication and marketing efforts toward city residents who experience health disparities. (Page 1014, Discussion Section)	(35)
*Examining the Implementation of Play Streets: A Systematic Review of the Gray Literature* Bridges et al. (2020)	Systematic search (Academic Search Complete, Google Scholar, Google) and screening of gray literature; RE-AIM (Reach, Effectiveness, Adoption, Implementation, Maintenance) guided data extraction. Final *n* = 36 documents.	Urban/suburban Play Streets documented via nonacademic sources (reports, news, web pages, etc.); synthesized using RE-AIM.	Multi-country sources; final sample from United States (*n* = 24), United Kingdom (*n* = 11), Australia (*n* = 1) → predominantly Global North.	Implementation details appeared in all items but with inconsistent depth; only 14/36 reported any effectiveness measures; most lacked standardized outcomes for RE-AIM components. Authors call for consistent reporting and a practical “how-to” implementation guide for Play Streets.	Incorporate common evaluation metrics to support future open street evaluations, such as motivation to host and cost/staffing requirements to maintain the initiative. (Page E8-E9, Discussion Section)	(84)
*Adaptation of the Recreovía During COVID-19 Lockdowns: Making Physical Activity Accessible to Older Adults in Bogotá, Colombia.* *González et al. (2024)*	Convergent mixed-methods, cross-sectional: in-depth interviews (program manager, instructors, older-adult users) + survey of older-adult users (*n* = 90; 59 outdoor, 31 indoor) + accelerometry during sessions + SOPARC park observations + PARA (Physical Activity Resource Assessment) park quality audit; integrated analysis.	Urban; Recreovía delivered via three modalities during lockdowns: (1) Facebook Live virtual classes, (2) “Asómate a la Ventana/Movement Route” balcony sessions, and (3) outdoor park classes with biosafety protocols (weekday and Sunday parks serving mostly low–middle SES areas).	Colombia (Bogotá) → Global South.	Recreovía rapidly adapted via virtual, balcony, and outdoor formats with safety protocols; 72–79% of older-adult participants met WHO PA guidelines during the study period; Recreovía days saw more park users (esp. women) and higher vigorous activity vs. non-program days; participants reported positive health and social experiences, with tech/data needs noted for virtual access.	Utilizing indoor and outdoor settings as alternatives to the Open Street program for older adults during public health emergencies. (Page 11, Discussion Section)	(61)
*CicLAvia: Evaluation of participation, physical activity and cost of an open streets event in Los Angeles* Cohen et al. (2016)	Quantitative observational evaluation combining: 14 route cameras (5-min interval counts), on-route intercept surveys at 5 hubs (duration, travel mode, etc.), and GPS volunteers from subsequent CicLAvia events to estimate speed/distance; then cost per MET-hour analysis.	Urban open-streets event (April 2014), 6-mile Wilshire Blvd route; evaluation focused on the event day.	United States (Los Angeles, California) → Global North.	Attendance estimated 36,800–54,740 active participants; average stay ≈3 h; 176,500–263,000 MET-hours generated; 45% would have been sedentary if not attending; cost per MET-hour (public funds) $1.29–$1.92.	Increasing open street programs’ reach, capacity, and frequency to lower costs. (Page 32, Conclusion Section)	(55)
*Dover Micro Open Street Events: Evaluation Results and Implications for Community-Based Physical Activity Programming* Suminski et al. (2019)	Multi-level, cross-sectional observational evaluation: (1) Individual exit intercept survey (every 3rd adult; 33 items) on demographics, PA, intentions, facilities, safety, social connectedness; (2) Program logs and hourly activity counts (EMA) for participation; (3) Neighborhood walkability audits within 0.25-mile radius. Descriptive analyses (SPSS).	Urban; five “Micro Open Streets Events” (MOSEs) in 2018 on 0.2–0.4 miles of closed streets, often paired with community events; activities included PA classes (e.g., Zumba), sports stations, healthy eating demos.	United States (Dover, Delaware) → Global North.	MOSE attendance ranged ~50–500 adults/event; survey *n* = 78 (≈65% response) showed attendees were largely female (≈69%), African American (≈58%), and overweight/obese (≈68%). 82% strongly liked the events; 80% very likely to attend again; 88% intended PA in next 7 days and ~43% attributed this to MOSE; ~49% learned about local PA facilities. Neighborhood audits indicated generally walkable, inviting routes (except one lower-amenity area). Authors conclude MOSEs are a viable alternative where full-scale Open Streets aren’t feasible.	A small-scale open street event can be easily disseminated and feasible for long-term physical activity participation. (Page 5, Implications for Public Health Section)	(85)
*Quality of Life, Physical Activity, and Built Environment Characteristics Among Colombian Adults* Sarmiento et al. (2010)	Cross-sectional study of adults (N ≈ 1,285) with multilevel modeling. Measures: WHOQOL-BREF (overall score), CDC Healthy Days (perceived health), WHOQOL future item; PA via IPAQ (transport/leisure; Ciclovía participation), BE from administrative data.	Urban; multistage sample of Bogotá neighborhoods; built-environment variables computed in 500-m buffers (density, diversity, design, distance to transit; Ciclovía length) using city datasets.	Colombia (Bogotá) → Global South.	Meeting leisure-time PA recommendations and Ciclovía participation were associated with higher HR-QOL and good/excellent perceived health; bicycling for transport was also positively associated. Land-use heterogeneity and parks (density/size) were positively associated with HR-QOL and optimism about the future; proximity/availability of mass-transit stations showed negative associations with HR-QOL; higher street network density related to lower perceived health/positivity.	_	(86)
*Network Analysis of Bogota’s Ciclovia Recreativa, a Self-Organized Multisectorial Community Program to Promote Physical Activity in a Middle-Income Country* Meisel et al. (2014)	Cross-sectional social network analysis of organizations (25 identified; 22 responded). Measures: ties; link attributes (integration, contact, importance); node attributes (leadership, years in program, sector). Analyses: descriptive/visual SNA + ERGM (GWESP, GWDegree/GWOdegree, GWDSP).	Urban; organizational network behind Bogotá’s weekly Ciclovía (multisector collaboration across government, transport, security, health, sports/recreation, etc.).	Colombia (Bogotá) → Global South.	Network centrality concentrated outside the health sector, especially Sports and Recreation (IDRD), Government, and Security; overall density ≈0.23, reciprocity ≈23.8%. Importance and structural tendencies (e.g., transitivity) were positively associated with integration, while more years in the program were negatively associated with integration; communities were multisectoral rather than siloed.	Community PA programs must engage with multiple sectors for widespread integration. (Page 12, “So What?” Box)	(73)
*Moving the Barricades to Physical Activity: A Qualitative Analysis of Open Streets Initiatives Across the United States* Eyler et al. (2015)	Qualitative phenomenology with structured telephone interviews; list of 47 events (2011), 27 organizers interviewed; audio-recorded, transcribed, and analyzed with constant comparative coding to generate themes.	Urban and suburban communities in the U. S.; interviews with lead organizers of Open Streets.	United States → Global North (organizers of U. S. Open Streets initiatives).	Initiation commonly driven by health and transportation goals; most aimed to reach the general population (some targeted families/children/neighborhoods). Key challenges: explaining the concept to communities, limited funding/personnel, and logistics. Authors conclude Open Streets democratize public space, promote physical activity/social connectedness, and warrant more evaluation and dissemination support.	Gather and foster local political support and collaboration. Incorporate open street initiatives in broader citywide agendas. Partner with academic institutions or organizations that regularly implement evaluations to assess open streets. (Page e57, Discussion Section)	(42)
*Moving targets: Promoting physical activity in public spaces via open streets in the US* Hipp et al. (2017)	Descriptive environmental scan + qualitative interviews (constant comparative analysis). Extracted program features: initiation year, frequency, duration, route length, participation, connectivity, transport access, safety, promotion, sponsorship, etc.	Urban/suburban Open Streets across the U. S.; web/social media review of 122 programs plus telephone interviews with 32 programs.	United States → Global North (national scan of U. S. programs).	As of Jan 2016, 122 U. S. programs; >75% started since 2010; typical scale: 1 date/year, ~4 h, route <5 km for 77%; many report 5,000–9,999 attendees. Funding, permitting, and branding are the main barriers to expanding dates; 13/32 interviewed programs hoped to reach ~12 dates/year. Authors note U. S. programs are generally smaller/less frequent than many Latin American Ciclovías.	Programs must reduce and expedite permitting requirements. New programs should consider different funding mechanisms for costs related to traffic control (such as policing, firefighters, and EMS). Open street programs should build a consistent brand and visibility to provide identity and encourage repeat participation and sponsorship. (Page S20, Conclusion Section)	(43)
*Translating evidence to policy: urban interventions and physical activity promotion in Bogotá, Colombia and Curitiba, Brazil* Díaz del Castillo et al. (2011)	Qualitative case-study design guided by the Physical Activity Policy Framework: literature/gray-literature review plus semi-structured stakeholder interviews (Bogotá ≈20; Curitiba ≈19), with framework/thematic analysis.	Urban programs: Bogotá’s Ciclovía Recreativa and Curitiba’s CuritibAtiva; analysis of how municipal and national policies across sectors (sports/recreation, urban planning, transport, environment, etc.) supported program development/sustainability.	Colombia (Bogotá) and Brazil (Curitiba) → Global South.	Policies that enabled both programs were primarily outside the health sector and depended on multisectoral collaboration; leveraging existing public infrastructure (streets, parks) was key to scale and sustainability; persistent barriers include traffic/business concerns (Ciclovía) and infrastructure/budget/identity challenges (CuritibAtiva).	Foster collaboration between international networks and multidisciplinary groups. (Page 350, Summary Box Research Section)	(44)
*Assessing the effect of physical activity classes in public spaces on leisure-time physical activity: “Al Ritmo de las Comunidades” A natural experiment in Bogota, Colombia* Torres et al. (2017)	Natural experiment with pre–post comparison (new vs. control parks) plus cross-sectional comparison (existing Recreovía); measures: IPAQ leisure-time PA, ActiGraph accelerometry, BMI; general linear mixed models with park random effect.	Urban; 9 public parks: 3 new Recreovía (Sunday 45-min classes, 8:00–12:00), 3 control (no program), 3 existing Recreovía (≥12 years). Community adults sampled from parks/households/groups; 6–8-month follow-up in new vs. control; accelerometer subsample.	Colombia (Bogotá) → Global South.	No overall PA change at 6–8 months comparing new vs. control parks; participants who began using the new Recreovía showed a modest increase in self-reported leisure-time MVPA (incl. Walking; p ≈ 0.06). Existing Recreovía users had significantly higher accelerometer-measured activity (vigorous, weekend, Sunday bouts) than nonusers; program showed meaningful reach (≈23% of eligible residents engaged), especially among women and lower-SES groups.	Selecting and targeting populations benefitting from the program is an optimal strategy. (Discussion Section, prior to limitations) Increase individuals’ participation by creating buddy systems, educational activities, and policy and environmental changes. (Recommendations for future studies Section)	(87)
*Implementation of childhood obesity prevention and control policies in the United States and Latin America: Lessons for cross-border research and practice* Pérez-Escamilla et al. (2021)	Comparative case-study synthesis drawing on scientific and gray literature and key-informant interviews; mapped to RE-AIM (reach, effectiveness, adoption, implementation, maintenance).	Policy/program case studies (national and urban): Open Streets/Play Streets (Bogotá, Colombia and US cities)	Multiple: United States and Latin American countries (Colombia, Bogota; US, San Francisco). Global North and Global South	Across cases, including Open Streets/Play Streets, successful implementation hinged on evidence-based advocacy, political will, and evidence of scalability/impact; programs required context-specific adaptations and would benefit from stronger monitoring/evaluation systems that track equity.	Multi-sectoral collaboration to encourage co-benefits of the program. (Pag. 8), Section 4: “Ciclovía in Bogotá, Colombia”	(68)
*Mixed method assessment of built environment and policy responses to the COVID-19 pandemic by United States municipalities focusing on walking and bicycling actions* Evenson et al. (2023)	Mixed methods: (1) Quantitative web audit of municipal websites (July–Sept 2020) + cross-check with the Shifting Streets COVID-19 Mobility Database; inter-rater reliability check on a 12% subsample. (2) In-depth interviews with 12 municipal Vision Zero leaders (Dec 2020–Jun 2021), recorded, transcribed, thematically coded.	Municipal actions affecting walking/bicycling during 2020 across all 314 U. S. municipalities with population ≥100,000; plus interviews with municipal transportation leaders.	United States (national sample of municipalities). → Global North	Identified 353 pandemic-related actions in 184/314 municipalities; ~294 actions likely facilitated walking/bicycling vs. 59 that likely limited it. Common actions: dedicating public space to dining (*n* = 139), closing streets to motor vehicles (*n* = 53), creating shared/slow/open streets (*n* = 24), reallocating lanes/curbs (*n* = 25), automating walk signals (*n* = 18), and free/reduced-cost bike share (*n* = 18). Larger cities implemented more actions than mid-size cities; interviews contextualized implementation and variability across places.	_	(88)
*Do Health Benefits Outweigh the Costs of Mass Recreational Programs? An Economic Analysis of Four Ciclovía Programs* *Montes et al. (2011)*	Average cost–benefit analysis using program director data + local surveys; defined Direct Health Benefit (DHB) from differences in annual medical costs for active vs. inactive adults; computed benefit–cost ratios and ran sensitivity analyses; also applied HEAT for cycling.	Urban, multisectoral Ciclovía/Open Streets programs (temporary street closures for recreation/PA) using existing road infrastructure.	Multicountry: Colombia (Bogotá, Medellín), Mexico (Guadalajara), USA (San Francisco). Global North and Global South	Annual per-capita program costs: US$6.0 (Bogotá), US$23.4 (Medellín), US$6.5 (Guadalajara), US$70.5 (San Francisco). Benefit–cost ratios for PA health benefits: 3.23–4.26 (Bogotá), 1.83 (Medellín), 1.02–1.23 (Guadalajara), 2.32 (San Francisco). Sensitivity: Bogotá most sensitive to prevalence of active bicyclists; Guadalajara to user costs; Medellín and San Francisco to operational costs. Overall, programs were cost-beneficial from a public health perspective.	With political support, other countries can benefit from road closures and existing infrastructure to promote PA. (Pag. 168, Discussion Section)	(48)
*Ciclovía participation and impacts in San Diego, CA: The first CicloSDias* Engelberg et al. (2014)	Multi-method observational evaluation: (1) City-wide RDD telephone surveys 1 week pre and post (*n* = 805); (2) On-route intercept survey of attendees (*n* = 713); (3) Observational counts to estimate attendance; (4) Business survey along the route (*n* = 26).	Urban open-streets event (Aug 11, 2013), 5.2-mile route, 10:00–16:00, 4 hubs; evaluation included on-route counts at 3 locations.	United States (San Diego, California) → Global North.	Estimated 8,311 attendees; attendees averaged 144 min PA at the event; 97% met 30-min/day and 39% met 150-min/week guideline during the event; 27% would have been inactive without the event. Awareness rose from 10% pre to 26% post city-wide; business impacts were mostly neutral/positive, though Latino/non-White residents were under-represented among attendees.	Community-based participatory research and including a more diverse population in outreach can increase the participation of the under-resourced. (Pag. 7; Discussion, “Event attendees” Section) Plan for sustainability and funding by engaging businesses. (Table 6)	(22)
*Bridging the gap between research and practice: an assessment of external validity of community-based physical activity programs in Bogotá, Colombia, and Recife, Brazil* Paez et al. (2015)	Qualitative case-study using RE-AIM; 17 key-informant interviews (coordinators, unit coordinators, instructors) in 2012; constant comparative analysis with member/expert validation.	Urban community PA programs: Bogotá’s Recreovía (RCP) and Recife’s Academia da Cidade (ACP); free exercise classes in public spaces (parks, plazas, etc.).	Colombia (Bogotá) and Brazil (Recife) → Global South.	Both programs primarily reach underserved populations and are implemented in public spaces; they offer free classes with educational/cultural components and have strong organizational structures. Effectiveness data are limited; funding and policy support are crucial for maintenance. Reporting external validity elements helps bridge research and practice.	PA promotion is underlined with free classes; funding is important for sustainability. (Pag. 1, Abstract)	(23)
*Talking the Walk: Perceptions of Neighborhood Characteristics from Users of Open Streets Programs in Latin America and the USA* *Zieff et al. (2018)*	Citizen-science, pre–post qualitative/observational study using the Stanford Neighborhood Discovery Tool (photo/voice) + brief surveys; ~50 adults completed paired walks (Bogotá 32; San Francisco 10; Temuco 8).	Urban Open Streets initiatives: community participants walked predefined routes on a non-event day and on an event day.	Colombia (Bogotá)—Global South; Chile (Temuco)—Global South; USA (San Francisco)—Global North.	Across sites, participants reported improved ease of walking, neighborhood safety, and friendliness on Open Street days; four cross-site themes, community/social connectedness, family-friendly environment, physical activity, and safety, were consistently identified, indicating social and health co-benefits of Open Streets.	_	(12)
*Active Living Logan Square Joining Together to Create Opportunities for Physical Activity* Gomez-Feliciano et al. (2009)	Descriptive case study using the Active Living by Design (ALbD) 5P model (preparation, promotion, programs, policy, physical projects); includes a community survey (≈400 residents) and implementation results/lessons learned.	Urban, predominantly Latino Logan Square neighborhood; school- and community-level initiatives (McAuliffe Elementary, Ames Middle), plus Open Streets pilots and advocacy for the Bloomingdale Trail (rails-to-trails).	United States (Chicago, Illinois) → Global North.	Partnership piloted Open Streets (two 4-mile events; later 8-mile), advanced the Bloomingdale Trail, and achieved school-based changes (new playgrounds; added recess; wellness councils). Success factors: full-time coordinator with community ties, culturally relevant strategies, and flexibility to pursue tangible, sustainable projects.	PA promotion can be sustainable by fostering good relationships, culturally sensitive programs, and the right staff. (Pag. 1; Lessons Learned of Abstract)	(24)
*Air quality impacts of a CicLAvia event in downtown Los Angeles, CA* Shu et al. (2016)	Natural experiment/observational: simultaneous air-quality measurements on CicLAvia route, control route, and nearby neighborhood using portable instruments (UFP via CPC/WCPC; PM₂.₅ via DustTrak). Traffic volumes measured at four points; freeway volumes from PeMS. Algorithms adjusted for meteorology using control ratios.	Urban open-streets event (CicLAvia, Oct 5, 2014) on ~16 km route through Downtown/East LA (incl. Boyle Heights). On-road and neighborhood monitoring conducted on three Sundays: pre-event (Sep 28), event (Oct 5), post-event (Oct 12); 3 sessions/day (8–15, 89).	United States (Los Angeles, California) → Global North.	Compared with expected (meteorology-adjusted) levels, on-road UFP fell ~21% and on-road PM₂.₅ fell ~49% during the event; community-wide PM₂.₅ fell ~12% on average (↑ in morning, likely due to set-up/arrival vehicles; ↓ at noon/afternoon). Route traffic dropped to zero during closure; nearby freeway volumes were unchanged.	_	(49)
*The Impact of a Temporary Recurrent Street Closure on Physical Activity in New York City* Wolf et al. (2015)	Observational program evaluation with three components: (1) Screen-line counts at 3 locations to estimate attendance; (2) on-route street-intercept survey (28 items) on demographics, usual PA, travel mode, and event activity; (3) traffic study (baseline vs. event day) to assess vehicular impacts.	Urban ciclovía (“Summer Streets”): 6.9 miles of Manhattan streets closed to motor traffic (three consecutive Saturdays, 7 a.m.–1 p.m.). Primary data collected on Aug 16, 2008 (second Saturday).	United States (New York City) → Global North.	Estimated ~50,000 participants (≈33 k pedestrians, 15 k cyclists, 2 k skaters). NYC participants accumulated ~72–86 min of moderate-equivalent PA on the route (walkers 3.6 mi; runners 4.3 mi; cyclists 6.7 mi). 24% of NYC attendees who usually do not meet PA guidelines still achieved ~26–68 min of moderate-equivalent PA at the event. 87% arrived via active/sustainable modes. No significant vehicular congestion/delays were detected on alternative routes. Demographically, participants skewed white, ages 25–64, and Manhattan-based relative to NYC overall.	Community groups are responsible for Play Streets’ operation to engage population’s PA. (Pag. 239; Discussion Section)	(56)
*Taking Physical Activity to the Streets: The Popularity of Ciclovia and Open Streets Initiatives in the United States.* Hipp et al. (2014)	Commentary summarizing motivations, stakeholders, and next steps; includes a table of motivations and outcomes and cites prior evaluations.	Narrative overview of Open Streets/Ciclovía initiatives across U. S. cities (diverse sizes and demographics).	United States → Global North	Open Streets in the U. S. expanded rapidly (90 + cities, 2008–2013); common motivations: increase PA, showcase active transportation, promote social cohesion, and support local economies. Stakeholder collaboration (policy makers, city agencies, businesses, community groups) is essential; sustainable funding/leadership and consistent evaluation are recurring needs. The table summarizes reported outcomes (e.g., per-participant PA minutes, spending, positive city perceptions) from prior studies.	A diversity of entities and stakeholders, such as policymakers, health insurance companies, funders, businesses, and communities, are required to sustain the program. (“Why Open Streets?” Section)	(90)
*Ciclovía initiatives: Engaging communities, partners and policymakers along the route to success* Zieff et al. (2013)	Document review + key informant communications synthesizing primary sources (year-end reports, budgets, MOUs, media, guidelines, prior evaluations) to answer three questions on development, implementation structures, and lessons learned.	Urban Open Streets programs: San Francisco Sunday Streets vs. St. Louis Open Streets; examines organization, route selection, programming, partnerships, outreach, merchant engagement, staffing/volunteers, and funding.	United States (San Francisco, California; St. Louis, Missouri) → Global North.	Both cities shared core implementation features (buy-in, inter-agency collaboration, route selection, programming, outreach/media, merchant support); they differed mainly in staffing/volunteer capacity and funding. Practical lessons include: formalized collaborations/MOUs, early route decisions, robust volunteer programs, strong promotion, and that longer routes and hours can increase reach.	Involve community partners, merchants, residents, and city agencies in the implementation process. Promote the intervention through media. (Page 10; Conclusion Section)	(91)
*Prevalence and Factors Associated with Walking and Bicycling for Transport Among Young Adults in Two Low-Income Localities of Bogotá, Colombia* Gómez et al. (2005)	Cross-sectional (May–Aug 2002); multistage, stratified household cluster sampling of *n* = 1,464 adults aged 18–29; 97.8% response rate. Outcomes from IPAQ (long, Spanish; culturally adapted). Logistic models examined socio-demographic, social, and environmental correlates (incl. Ciclovía use).	Urban, two low-income localities: Tunjuelito (mostly flat terrain) and Santa Fe (hilly). Population-based sample of young adults.	Colombia (Bogotá) → Global South.	Prevalence: bicycling ≥10 min in last week 16.7%; walking ≥90 min in last week 71.7%. Bicycling more likely among residents of Tunjuelito, Ciclovía users, and those with leisure-time PA; less likely among women and those with higher education. Walking more likely among those with regular leisure-time PA; less likely among housewives/househusbands and residents of Tunjuelito. Authors highlight the need for targeted, context-specific strategies (e.g., gender-responsive approaches; terrain considerations) to promote active transport.	Consider context to develop culturally appropriate approaches. (Page 456; Discussion Section)	(65)
*Results from Chile’s 2018 Report Card on* *Physical Activity for Children and Youth* Aguilar-Farias et al. (2018)	Evidence synthesis/scorecard: scientific and gray-literature search; Advisory + Scientific committees; grades A–F per Alliance rubric; data largely self-report and mostly adolescents.	National: synthesis of multiple nationally representative surveys, published studies, and official reports to grade 13 indicators (10 core: Overall PA, Sport, Active Play, Active Transportation, Sedentary Behaviors, Physical Fitness, Family and Peers, School, Community and Environment, Government; plus Sleep, Inclusion, Overweight/Obesity).	Chile → Global South.	Overall PA = D- (≈20% meet guidelines); Community and Environment = B, Government = B-; Active Transportation = F; Sedentary Behaviors = C-; Physical Fitness = D; Family and Peers = F; School = D; Active Play, Sleep, Inclusion = Incomplete. Authors note reliance on self-report and limited data for younger children.	_	(92)
*Policy and Built Environment Changes in Bogota´ and their Importance in Health Promotion* Parra et al. (2007)	Narrative policy/urban health case analysis summarizing municipal initiatives, context, and plausibly related PA/quality-of-life impacts; cites surveys and prior studies (e.g., leisure-time PA, active transport prevalence; cross-sectional associations with Ciclovía/Ciclorutas).	Urban policy and environment changes citywide: weekly Ciclovía (≈117 km, Sundays/holidays 7:00–14:00; ≈400,000 participants), Ciclorutas bicycle network (≈300 km), TransMilenio BRT, park system expansion, Car-Free Day, Pico y Placa (plate-based driving restrictions), and public-space recovery initiatives.	Colombia (Bogotá) → Global South.	Bogotá enacted multi-sector changes—transport, recreation, urban design—that reclaimed public space, expanded active-mobility infrastructure, and likely facilitated physical activity (e.g., high Ciclovía participation; bicycle-path access linked to cycling; women who frequently used Ciclovía more likely to be active in leisure time). The city also reported park area growth (≈2.5 → 4.12 m^2^ per capita, 2001–2003) and traffic/pollution improvements via Pico y Placa and Car-Free Day. Authors call for a research/evaluation agenda to better quantify health impacts and guide policy.	Increase awareness among the population about using the structure available for Open Street programs. Encourage merchants and businesses to support the community goal of open street programs. (Pag. 347; Discussion Section)	(50)
*Participation and physical activity in Recreovia of Bucaramanga, Colombia* Paredes Prada et al. (2021)	Cross-sectional, observational study using SOPARC/iSOPARC at fixed sites. Outcomes: counts by sex, age group, activity type, and PA intensity (sedentary/moderate/vigorous). Reliability assessed (ICC > 0.95). Descriptive stats with χ^2^ tests.	Urban Recreovía (2.5-km route; Sundays 8:00–12:00). Observations over 5 Sundays (Sep–Nov 2017) at 4 points: two on-street count sites and two aerobics-class areas.	Colombia (Bucaramanga) → Global South.	38,577 observations (streets *n* = 34,969; classes *n* = 3,608). On streets: majority men (≈63%), adults (≈62%), and MVPA (≈98%); top activities bicycling 49.6%, walking 30.7%, jogging 9.2%. In classes: mostly women (65%), adults (89%), with vigorous PA ≈ 76%; participation peaked later in the morning; site differences reflected local context (e.g., park proximity). Overall, Recreovía supported high levels of MVPA across users.	_	(37)
*La promoción de estilos de vida saludable aprovechando los espacios públicos* Peña-de León et al. (2017)	Mixed methods (two stages): (1) non-participant observation of route attributes using Servipanoramas/Third Place concepts (field notes and photos); (2) Online survey of adults (*n* = 168 analyzed; from 281 collected) using scales for perceived environment, physical activity, and subjective wellbeing; multiple regression tested associations.	Urban: “Ruta Recreativa” (open-streets) in Saltillo; ~13 km route, Sundays 7:00–13:00, with separated lanes and on-route amenities.	Mexico (Saltillo, Coahuila) → Global South.	The route’s attractiveness and perceived safety were positively associated with visitors’ wellbeing (model R^2^ ≈ 0.74, *p* < 0.01), while perceived physical “quality” showed no effect; visitors rated safety, cleanliness, and separated lanes highest, and noted gaps (e.g., drinking fountains, public toilets, rest areas). The program operates as a multisectoral third place, enabling health-promotion campaigns and social interaction alongside recreation.	Ensure widespread dissemination of the open street program and its benefits. (Pag. 206; Qualitative Results Section)	(54)
*Logic model of the Recreovía: a community program to promote physical activity in Bogota* Rios et al. (2017)	Logic model development following the CDC Physical Activity Evaluation Handbook; mixed-methods convergent approach: semi-structured interviews with current and former coordinators, document/administrative data review (historic users, instructors, budget, sites); descriptive analysis.	Urban community PA program delivering free, instructor-led classes in public spaces (parks, plazas, community centers, commercial areas; also a penitentiary); designed to complement Ciclovía. ~41 points operating across weekdays and weekends.	Colombia (Bogotá) → Global South.	The logic model specifies resources (e.g., 59 staff, partners across multiple sectors; 2015 budget ≈ USD 873,098), activities (aerobics/strength/mobility/rumba/Pilates; weekday and weekend schedules; mass events; instructor training), and outputs/outcomes (e.g., 2015: 1,879 working days of activity; 641,956 users; ~999 weekend sessions with 426,461 users; 609 weekday sessions with 142,697 users). Short-term goals include expanding points/participation and partnerships; long-term goals include increasing PA, reducing sedentary behavior/obesity, and institutionalizing Recreovía as a priority city program.	Strengthen alliances with the public and private sectors. (Pag. 210; “Short-Term Results” Section)	(67)
*Criterios técnicos para implementación de una ciclovía recreativa* Dirección General de Promoción de la Salud, Ministerio de Salud del Perú (2015)	Implementation guidance with step-by-step procedures: (1) intersectoral coordination; (2) planning and programming; (3) budgeting via Programa Presupuestal 101; (4) execution criteria; (5) final reporting to regional health authorities; includes checklists, participant count and survey procedures, and annexed data-collection forms.	Municipal, urban public-space program: temporary street closures one fixed day/week (typically Sunday) for ~4 h with five required modules (3 physical-activity, 1 healthy eating, 1 health promotion); minimum route length tied to population (<80,000: ≥1 km; ≥80,000: ≥2 km).	Peru → Global South.	Although not an empirical study, the manual codifies operational standards: community engagement and detour plans; staffing and volunteer roles; standardized participant counts (e.g., fixed 3-min windows repeated hourly) and brief adult surveys; module installation/teardown, safety and surveillance protocols; and required contents for the final report (participation totals, module logs, photos, materials, checklists). It positions Ciclovía Recreativa as a public-health strategy to promote activity and healthy behaviors through recurring, well-managed street openings.	Implement health education sessions for physical activity, nutrition, and wellness. (Pag. 12) Utilize public health representatives to stress the program’s importance and justification for health and physical activity promotion. (Pag. 11) Use of static publicity of the programs in widely used spaces. (Pag. 11)	(66)
*Open Streets for Whom? Toward a Just Livability Revolution* Slabaugh et al. (2021)	Environmental justice–oriented framework: authors present four components (distributional, interactional, procedural, recognitional) and analyze six potential paradoxes arising in open-streets planning; conclude with policies, programs, partnerships to advance equity.	Urban public space/transportation planning: initiatives that reallocate road space to pedestrians, cyclists, and other non-motorized modes (“open/slow/safe streets”).	Not country-specific; primarily U. S. examples with global relevance (notes ~600 cities worldwide undertaking open/slow/safe streets by Jan 2021). → Global North Focus	The article argues that open-streets interventions can both advance and undermine justice, depending on distribution, process, recognition, and interactions. It surfaces six paradoxes (e.g., displacement, hegemony, safety, White spaces, engagement, stigma) and proposes equity-focused policies, programs, and partnerships to navigate them.	Introduce anti-displacement policies and strategies. Create a policy to site open streets near schools/community centers. Explore and enact community-based alternatives to policing. Provide technical assistance to BIPOC businesses in adapting their strategies. Build partnerships within the environmental justice community. Understand and leverage community power. Build an anti-racist planning culture from within. Encourage agenda setting for open streets by BIPOC residents. (Pag. 5)	(63)
*From “streets for traffic” to “streets for people”: can street experiments transform urban mobility?* Bertolini (2020)	Narrative literature review and discussion; proposes typology (re-marking streets; re-purposing parking; re-purposing sections; re-purposing entire streets) and a framework based on “transition experiments.”	Urban streets/public space and mobility, city street experiments (temporary changes to use/regulation/form).	Dominance of North American and Latin American cases. → Global North and Global South	The review identifies four types of city street experiments (from simple re-markings to full street re-purposing) and summarizes reported impacts, generally positive for physical activity, active transport, safety, and social interaction, with mixed effects on business. It introduces a transition-experiments–based framework to assess whether and how such experiments can catalyze systemic urban mobility change; evidence of broader transformation remains limited.	_	(93)
*Community Benefits and Lessons for Local Engagement in a California Open Streets Event: A Mixed-Methods Assessment of Viva CalleSJ 2018* Douglas et al. (2019)	Mixed-methods evaluation: on-route participant survey (*n* = 1,571); 114 interviews with residents, businesses, and participants; participant observation; review of social media and an augmented-reality (Pokémon Go) component.	Urban Open Streets event “Viva CalleSJ” on Sept 23, 2018; ~6-mile corridor, 10:00–15:00; city’s estimate ~125,000 attendees; activities at multiple hubs.	United States (San José, California) → Global North.	Participants reported high satisfaction, strong sense of community, and ≥1 h of physical activity for most; many arrived by car or bike and made on-route purchases (esp. food/drink). Most local businesses/residents viewed the event positively, though outreach gaps left some under-informed or concerned about closures; recommendations emphasize stronger advance engagement with merchants and neighborhoods.	Ensure targeted outreach to businesses and community-based organizations to increase participation. (Pag. 24: Implications for the VIVA CALLESJ program in the future Section)	(57)
*A Survey of Viva CalleSJ Participants: San Jose, California 2016* Agrawal and Nixon (2016)	Quantitative descriptive on-route survey: one-page, self-complete paper questionnaire; convenience sampling at 5 locations; *n* = 318 usable responses; topics: how heard, travel to event, mode on route, PA time, activities, spending, demographics.	Urban Open Streets event (Viva CalleSJ) on Sept 18, 2016, 10:00–15:00, 6-mile route through Burbank, Downtown, Japantown, Willow Glen; city estimated ~100,000 attendees.	United States (San José, California) → Global North.	Most respondents reported >60 min of PA at the event (≈72%), arrived by bike (51%) or car (32%), and used bike (65%) or walk (37%) along the route; word of mouth (41%) and social media (33%) were top information sources. About 39% expected to spend $21+, and common purchases were food trucks (35%), restaurants (24%), and stores (21%). Demographics skewed young/middle-aged; 84% lived in San José.	Encourage people to market the open street event to peers through multiple channels. Utilize entertainment, food trucks, and resource tables to attract participants. (Pag. 15: Implications for the VIVA CALLESJ program in the future Section)	(94)
*Mapping Equality in Access: The Case of Bogotá’s Sustainable Transportation Initiatives* *Teunissen et al. (2014)*	GIS-based spatial accessibility analysis using 2009 network data (TransMilenio lines/stations and feeders; Cicloruta; Ciclovía) and 2005 census SES blocks. Catchments: TransMilenio walking-time buffers (5–30 min at ~4.4 km/h); Cicloruta/Ciclovía distance buffers (0.5–3 km). Descriptive comparisons and hypothetical network expansions to assess equity gains.	Urban: three initiatives, TransMilenio BRT, Cicloruta bicycle network, and Ciclovía open streets. Focus on spatial access by socioeconomic stratum (SES).	Colombia (Bogotá) → Global South.	TransMilenio: spatial access is fairly equal and often better for low/middle SES at short walks, yet use is lower among low SES (affordability and service factors). Cicloruta: access favors middle/high SES, while cycling share is highest in low SES, suggesting unmet infrastructure equity. Ciclovía: access highest for high SES areas, but most users are low/middle SES; modest route extensions toward low-SES areas could substantially improve equity.	_	(40)
*The Open Streets Guide* Alliance for Biking and Walking (2012)	Descriptive compilation and methods guide: web/print scan, organizer interviews, and a 10-question online survey; synthesized into seven model types (Seattle, Cleveland, San Francisco, Portland, Winnipeg, Savannah, Kentucky) + website companion (OpenStreetsProject.org)	Urban open streets initiatives: temporary street closures for people (walking, biking, play, social activities). Covers route types, settings, frequency, organization, funding, and supporting activities.	Primarily United States and Canada (Seattle, Cleveland, San Francisco, Portland, Winnepeg (Canada), Savannah, Kentucky (statewide)) → Global North focus	By early 2012 there were ~70 initiatives in North America (67 documented). Typology of seven models by organizing/funding patterns; common route types (neighborhood linear, loop, arm-and-loop, multi-neighborhood linear, regional linear) and settings (parks, parkways, residential, neighborhood centers, downtowns). Snapshot stats: 28% of cities <100 k population; 45% publicly organized; 52% publicly–privately funded; 54% use multiple route settings; 73% include supporting activities; 16% run weekly in season. Benefits framed across public health, environmental, economic, and social domains.	(1) Open street organizers should undertake several concurrent political and logistical planning efforts that successfully move the proposal from concept to implementation. (2) Open street organizers should build a willing coalition of advocacy, municipal, and/or private-sector supporters. (3) Establishing political support from the mayor, city council, and/or other political representatives is important because elected officials most commonly allocate the public resources needed for implementation. (4) Once government and political buy-in is obtained, the municipal leaders should assign the appropriate department to organize the initiative or serve as the liaison between the city’s dedicated resources and the lead organizing entity. (5) Acquire Municipal Funding and/or Significant In-Kind Support from Public authorities should lead in dedicating public funds and/or resources. (6) Organizers should critically evaluate and share the successes and failures to improve the next effort. (Pag. 156–160)	(64)
Influences of Built Environments on Walking and Cycling: Lessons from Bogota *Cervero et al. (2009)*	Cross-sectional, multistage household survey + GIS measures; 30 neighborhoods selected, 5 blocks per neighborhood, 10 households per block; 1,500 respondents (66.7% response rate). IPAQ long form adapted; subsample accelerometer validation (*ρ* = 0.42; test–retest *r* = 0.69). Multilevel logistic models relating “5Ds” built-environment factors to utilitarian walking/cycling.	Urban metropolitan context (Bogotá), examining neighborhood and “extended neighborhood” (1,000 m) scales.	Colombia (Bogotá) (Global South)	In Bogotá, street connectivity and street density were the strongest built-environment predictors of walking ≥30 min/day for utilitarian purposes; at a broader scale, proximity to TransMilenio stations also increased odds of walking. In Bogotá, street connectivity and street density were the strongest built-environment predictors of walking ≥30 min/day for utilitarian purposes; at a broader scale, proximity to TransMilenio stations also increased odds of walking. Classic “5D” factors like overall density and land-use mix were not significant in this context (limited variation), whereas street design (connectivity/density) and nearby reserved lanes encouraged Ciclovía use.	_	(39)

PA, physical activity; SES, socioeconomic status; MVPA, moderate-to-vigorous physical activity; SED, sedentary time; BMI, body mass index; CBPR, community-based participatory research; GIS, geographic information system; SC, social capital; OR, odds ratio; HRQoL, health-related quality of life; LS, life satisfaction; EORTC QLQ-C30, European Organisation for Research and Treatment of Cancer Quality of Life Questionnaire, Core 30; IPAQ, International Physical Activity Questionnaire; SOPARC, System for Observing Play and Recreation in Communities; ICC, intraclass correlation coefficient; PARA, Physical Activity Resource Assessment; RE-AIM, Reach, Effectiveness, Adoption, Implementation, Maintenance; EMA, ecological momentary assessment; BRT, bus rapid transit; IDRD, Instituto Distrital de Recreación y Deporte; and EMS, emergency medical services.

**Figure 2:**
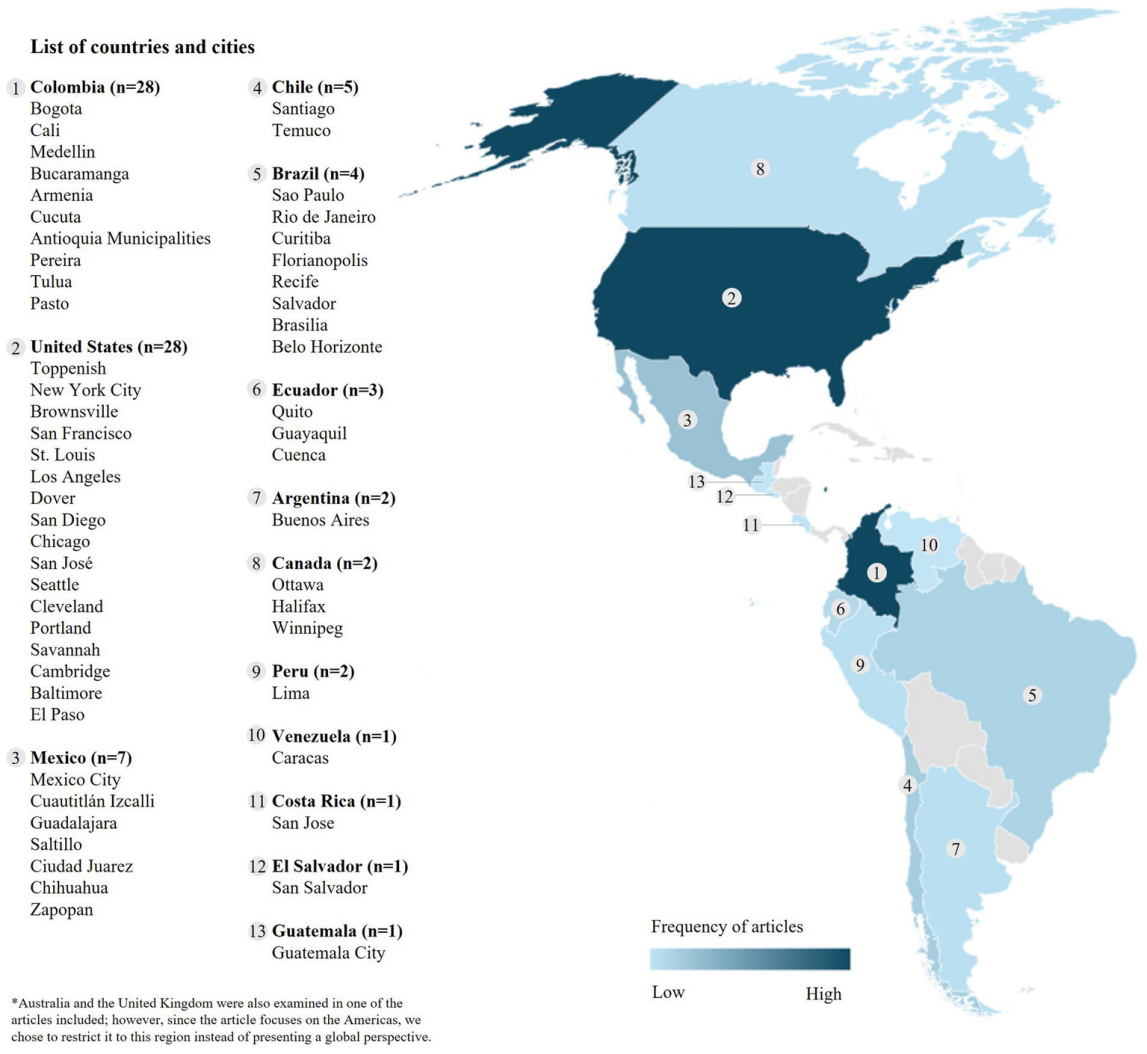
Map of the Distribution of Countries and Cities Represented in the Studies included in this Scoping Review.

The distribution of articles in this review demonstrates that Colombia and the US dominate the evidence base (see Figure 2), each contributing 28 studies, reflecting the long-standing Ciclovia in Bogota and the rapid expansion of Open Streets in US cities. Mexico (*n* = 7), Chile (*n* = 5), and Brazil (*n* = 4) also had notable representation, while several other Latin American countries and Canada contributed fewer studies (1–3 each). It is essential to note that the total number of articles exceeds 59 in the map, as many articles encompass multiple countries in their studies. Most of the studies were set in urban Latin America, particularly Bogotá, Colombia. Bogota’s programs alone attract approximately 400,000 weekly participants, with route lengths exceeding 117 km (50). Other Latin American sites included Mexico City, where Muévete en Bici averages approximately 21,800 participants weekly across a 55-km route (51), as well as Santiago de Chile (17), Cali (52), Quito (18), Saõ Paulo (53), Saltillo (54), and multiple other Latin American cities. In the US, evaluations focused on single events or city initiatives, such as CicLAvia in Los Angeles, which engages approximately 36,800 to 54,700 participants in one event and generates176,000–263,000 MET-hours of PA (55), Summer Streets in New York City, with approximately 50,000 participants in a single day (56), and Viva CalleSJ in San José, with attendance estimates up to 125,000 (57). Rural settings were less common, with only one rural town in this review, Toppenish, Washington, reaching approximately 200–394 participants per hour, which presented 2–4% of the town’s population (45, 46).

Across contexts, Open Streets consistently promoted PA and social connectedness. Participants frequently met or exceeded daily or weekly PA guidelines during events. In Mexico City, 88.4% of users met the 150 min/week recommendation during the Muévete en Bici event, adding approximately 71 min of MVPA weekly (51). In Los Angeles, 45% of CicLAvia attendees reported they would have otherwise been sedentary (55), while in San Diego, 97% met the 30-min/day threshold and 39% met the 150-min/week guideline during CicloSDias (22). Open streets fostered equity and inclusion, with essential differences in context. In Bogotá and Santiago de Cali, Ciclovía participants crossed neighborhoods of varying socioeconomic status, supporting social inclusion (17). Yet inequities in geographic access are even more evident: in Bogotá, the median distance to Ciclovía was 2,938 meters for the lowest socioeconomic status, compared to 482 meters for the highest socioeconomic status, a six-fold disparity (58). In contrast, US programs were smaller and often attracted disproportionately white, higher-income participants, as seen in St. Louis, where attendees were primarily white and college-educated despite the city’s demographics (35). However, targeted outreach and community-led adoption were successful in a rural Open Streets event in Toppenish, Washington, where attendance at the event grew and participants from Hispanic/Latino and American Indian backgrounds were engaged (45, 46).

Findings varied by subpopulations, among children in Bogotá, frequent Ciclovia users engaged in more MVPA on Sundays; however, they had higher BMI z-scores compared with non-users (59). Recreovía increased PA among women in Bogotá, with 75% meeting MVPA guidelines compared with 61% in non-program parks (60). During the COVID-19 pandemic, adaptations such as virtual and balcony-based sessions enabled 72–79% of older adults to meet WHO PA guidelines (61). Environmental factors, such as infrastructure quality, safety, and political support, are consistently linked to sustainability and scalability (12, 40, 42, 62). For example, Diaz del Castillo et al. identified flexibility, instructor training, public funding, and community champions as key to maintaining programs that reached more than 1,455 communities in Colombia (41). Similarly, economic evaluations demonstrated that Ciclovia’s were highly cost-effective in Latin America, with cost–benefit ratios exceeding 3.0 in Bogotá, while US programs had higher per-capita costs (48).

### 3.2 Qualitative results

We identified 63 strategies for Open Streets programs from the 59 studies reviewed in this analysis (see Supplementary Table 8). The identified Open Street strategies were matched with 52 ERIC strategies.

**Figure 3:**
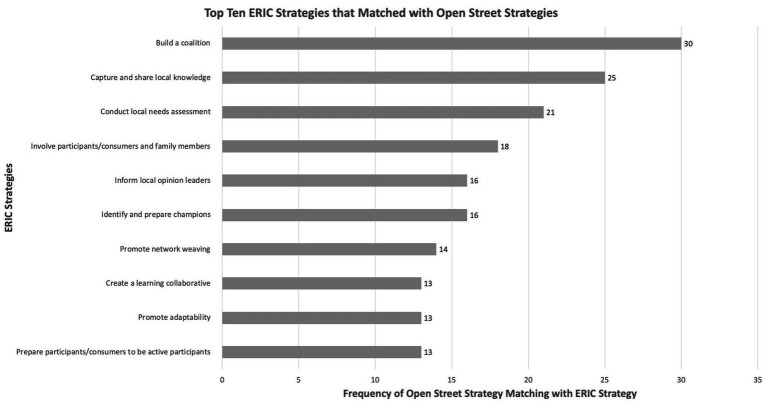
Top Ten ERIC Strategies that Matched with Open Streets Strategies.

Figure 3 presents the top ten ERIC strategies matched with the Open Streets strategies identified in this review. The most aligned ERIC strategy identified was “Build a coalition” (*n* = 30), followed by “Capture and share local knowledge” (*n* = 25), and “Conduct a local needs assessment” (*n* = 21). Other frequently matched strategies included “Involve participants/consumers and family members’ (*n* = 18), followed by “Identify and prepare champions” and “Inform opinion leaders’ (*n* = 16 each). Additionally, the strategies “Promote network weaving” (*n* = 14), “Prepare participants/consumers to be active participants,” “Promote adaptability,” and “Create a learning collaborative” were each matched with 13 Open Streets strategies. Using the thematic analysis approach, we categorized the identified Open Streets strategies into nine themes and 52 sub-themes (see Supplementary Table 5). Table 4 presents example quotes from Open Streets strategies in studies included in the review, organized by theme and the two most prevalent sub-themes.

**Table 4:** Themes and subthemes of open street strategies.

Themes	Subthemes/ERIC strategies	N	Open streets strategies	Example quotes of open streets strategies of the included studies	References
Use evaluative and iterative strategies	1.1 Conduct local needs assessment	21	Encourage agenda setting for open streets by BIPOC residents.	*“Encourage agenda setting for open streets by BIPOC residents.”*	(63)
	1.2 Assess for readiness and identify barriers and facilitators	9	Apply tactical urbanism to Ciclovia programs to promote mobility during public health emergencies.	*“Taking advantage of the temporal nature of the Ciclovía program, as well as, its capability of enhancing the public space function of city streets, over the past few months, urban planners have explored how to apply tactical urbanism measures as a way to respond to all the mobility challenges that have emerged to the COVID-19 pandemic.”*	(17)
Provide interactive assistance	2.1 Facilitation	8	Acquire Municipal Funding and/or Significant In-Kind Support Public authorities should take the lead in dedicating public funds and/or resources.	*“Acquire Municipal Funding and/or Significant In-Kind Support Public authorities should take the lead in dedicating public funds and/or resources.”*	(64)
	2.2 Centralize technical assistance	2	Provide technical assistance to BIPOC businesses in adapting their strategies.	*“Provide technical assistance to BIPOC businesses to adapt strategy.”*	(63)
Adapt and tailor to context	3.1 Promote adaptability	13	Budget allocation and accountability improved and scaled up the programs.	*“Coldeportes allocated national funds, and the departments committed to a progressive increase in local funding. This strengthened existing initiatives and created new programs.”*	(41)
	3.2 Tailor strategies	10	Consider context to develop culturally appropriate approaches.	*“Develop culturally appropriate strategies for promoting physical activity and active commuting, especially among women.”*	(65)
Develop stakeholder interrelationships	4.1 Build a coalition	30	Gather and foster local political support and collaboration.	*“The first necessary component was local political support and collaboration. Support from top city officials and department leads has been identified as a key factor in many community initiatives.”*	(42)
	4.2 Capture and share local knowledge	25	Understand and leverage community power.	*“Develop shared understandings of race, privilege, power.”*	(63)
Train and educate stakeholders	5.1 Create a learning collaborative	13	Capacity building increases the quality of the program.	*“Both programs emphasized staff training.”*	(41)
	5.2 Conduct educational outreach visits	12	Implement health education sessions for physical activity, nutrition, and wellness.	*“Five modules installed: three on PA promotion, one on healthy eating, and one on health promotion.”*	(66)
Support practitioners	6.1 Facilitate relay of data to practitioners	2	Strengthen alliances with the public and private sectors.	*“Strengthen alliances with the public and private sectors.”*	(67)
	6.2 Develop resource sharing agreements	10	Plan for sustainability and funding by engaging businesses.	*“Increasing targeted outreach through partnerships with ethnic-specific media outlets, more promotion in stores and clinics in disadvantaged neighborhoods, promoting event attendance through partnerships with employers of highly diverse employees, and engaging ongoing advisors from underserved communities.”*	(22)
Engage consumers	7.1 Involve patients/consumers and family members	18	Increase awareness among the population about using the structure available for Open Street programs.	*“Increasing awareness and use of environmental resources that are already available in the community may be a cost-effective strategy to increase physical activity and health of the population.”*	(50)
	7.2 Prepare patients/consumers to be active participants	13	Active transportation can be socially transmitted and become a norm to promote PA in Open Street programs.	*“Community norms around active transportation can be created by increasing awareness and accessibility for active transportation.”*	(46)
Utilize financial strategies	8.1 Alter incentive/allowance structures	12	Programs must reduce and expedite permitting requirements.	*“Evidence shows Open Streets provide a space for physical activity and social interaction, but without scale cannot achieve a community-wide culture of health. A replicable structure or model may (1) reduce and expedite permitting requirements, (2) decrease costs related to safety, policing, and intersection control, and (3) build a consistent brand and visibility to provide identity and encourage repeat participation and sponsorship. Without policy to support the expansion of initiatives, in frequency, length, and number, the impact Open Streets can have in promoting community-wide physical activity in public space is limited. Policy is best made with a firm evidence base, and more consistent and robust evaluation is necessary across initiatives.”*	(43)
	8.2 Access new funding	5	Establishing political support from the mayor, city council, and/or other political representatives is important because elected officials most commonly allocate the public resources needed for implementation.	*“Establishing political support from the mayor, city council, and/or other political representatives is important because elected officials most commonly allocate the public resources needed for implementation.”*	(64)
Change infrastructure	9.1 Change physical structure and equipment	7	Utilizing indoor and outdoor settings as alternatives to the Open Street program for older adults during public health emergencies.	*“The decision-making process adapted to the regulations in place and the demand for services, and the health sector recommendations based on the progressive evidence about COVID-19 transmission. Activities in the indoor and outdoor settings were alternatives for older adult users of the Recreovía program to continue being physically active during the strictest period of the pandemic regulations.”*	(61)
	9.2 Start a dissemination organization	4	Use of static publicity of the programs in widely used spaces.	*“Use of static publicity in gyms, parks, malls, schools, land terminals, highlighting the importance of PA and healthy eating.”*	(66)

#### 3.2.1 Theme 1: use evaluative and iterative strategies

This theme emphasizes the development of evaluative and iterative strategies for the Open Streets program, including planning and responding to public health emergencies. The Open Street strategy, “Encourage agenda setting for open streets by BIPOC residents,” is presented in a conceptual paper by Slabaugh et al. as a part of the “partnerships” category. The authors stress the following: “planners build reciprocal partnerships with racial and environmental justice organizations” and conduct engagement with BIPOC residents, particularly “at the agenda-setting stage for open streets” (63). This shift in decision-making power for BIPOC communities, not just city officials, to define the priorities and purposes of open streets, could ensure interventions reflect the lived experiences and local needs (63). This relates directly to the ERIC strategy of “Conducting local needs assessments” (sub-theme 1.1); both strategies center BIPOC expertise and context-specific knowledge: agenda setting empowers residents to shape the vision of Open Street events, while needs assessments could provide the evidence base for what priorities should be, thereby aligning planning and community-defined needs rather than imposing external agendas.

The next Open Street strategy, “Apply tactical urbanism to Open Street programs to promote mobility in time of health emergencies,” is presented in a study by Mejia-Arbelaez et al., in the context of how Ciclovia programs can temporarily transform streets into democratic, flexible spaces that respond to crises such as the COVID-19 pandemic by allowing safe mobility and recreation. This translates to using low-cost, rapid, and adaptable interventions (e.g., pop-up bike lanes, temporary road closures) to sustain access to physical activity and social inclusion during emergencies (17). This relates to the ERIC strategy, “Assess for readiness and identify barriers and facilitators” (sub-theme 1.2), because before deploying tactical urbanism, cities must evaluate community capacity, political will, and logistical obstacles to ensure that emergency responses are both feasible and effective.

#### 3.2.2 Theme 2: provide interactive assistance

Interactive assistance between public authorities, the community, event organizers, and participants is essential for implementing and sustaining Open Streets programs. The Open Street strategy, “Acquire Municipal Funding and/or Significant In-Kind Support from Public authorities should lead in dedicating public funds and/or resources,” is in the Open Streets Guide developed by the Alliance for Biking and Walking. It is presented in the guide as a “best practice” to help ensure program sustainability, highlighting that when cities commit budgetary resources or staff time, it signals institutional support and could allow initiatives to grow beyond pilot phases (64). Municipalities should not only permit Open Street events but also actively invest in them, for example, through direct funding, police and staff time, or logistical support (64). This aligns with the ERIC strategy of “Facilitation” (sub-theme 2.1) because public agencies’ in-kind resources (e.g., traffic control, permits, staffing) directly facilitate the safe and consistent operation of open streets, which can lower barriers for community partners and aid in implementation (64). The next Open Street strategy, “Provide technical assistance to BIPOC businesses in adapting their strategies,” is presented in a conceptual paper by Slabaugh et al., in the context of programmatic responses that help navigate paradoxes such as displacement and safety by supporting BIPOC-owned businesses to participate in and benefit from Open Streets (e.g., permitting for outdoor dining, materials support) (63). Offering resources and guidance to BIPOC businesses could help in adjusting to new street uses rather than being excluded or displaced (63). This relates to the ERIC strategy “Centralized Technical Assistance” (sub-theme 2.2) because while centralized creates a broader support system, targeted assistance to BIPOC businesses ensures equity by addressing specific inequities and barriers they face within that system.

#### 3.2.3 Theme 3: adapt and tailor to context

Adapting Open Streets programs to local contexts could help increase their relevance, accessibility, and sustainability across different localities. The Open Street strategy, “Budget allocation and accountability to improve and scale up program,” is presented in the results of a study by Díaz Del Castillo et al., as a part of the continuation and growth of Colombia’s HEVS (Healthy Habits and Lifestyles) program, where a co-funding scheme launched in 2008 allowed national and local governments to dedicate resources, hire trained personnel, and require accountability of program guidelines and goals (41). Providing financial resources and connecting them with clear responsibilities transformed policies from paper commitments into sustained, concrete actions (41). This relates to the ERIC strategy of “Promoting Adaptability” (sub-theme 3.1) because stable funding and accountability mechanisms give the programs the flexibility to adjust to changing conditions while ensuring they continue scaling up effectively.

The next Open Street strategy, “Consider context to develop culturally appropriate approaches,” is presented in a study by Gómez et al., where the authors emphasize that differences in gender, socioeconomic status, and neighborhood terrain (flat vs. hilly) affect walking and bicycling behaviors in Bogotá (65). Interventions should be designed with sensitivity to local cultural, social, and environmental conditions rather than relying on models from high-income countries (65). This relates directly to “Tailor Strategies” (sub-theme 3.2), because tailoring requires adapting interventions to these contextual differences to ensure effectiveness and equity in promoting active transport in Open Street events.

#### 3.2.4 Theme 4: develop stakeholder interrelationships

Theme four emphasizes building strong networks and stakeholder collaborations to support physical activity promotion programs. The Open Street strategy, “Gather and foster local political support and collaboration,” is presented in a study by Eyler et al. as a necessary foundation for Open Streets success, since strong backing from city officials and interdepartmental collaboration reduced costs and helped embed initiatives into broader agendas such as transportation and sustainability (42). Engaging elected leaders and agencies to champion and sustain Open Streets through advocacy, visibility, and shared resources (42). This is related to the ERIC strategy “Build a Coalition” (sub-theme 4.1), because political support becomes more effective when connected to coalitions of community groups, businesses, and health advocates working together toward long-term sustainability. The next Open Street strategy, “Understand and leverage community power,” is presented in a conceptual paper by Slabaugh et al., in the section on partnerships and institutional culture, using tools such as the City of Austin’s (Texas) equity assessment that grew out of grassroots organizing (63). Recognizing the influence and expertise that communities, particularly BIPOC residents, bring, and using that power to reshape policies and planning processes toward justice (63). This is related to the ERIC strategy “Capture and share local knowledge” (sub-theme 4.2), as both strategies center on community expertise, with one emphasizing power as a lever in decision-making and the other emphasizing knowledge as a resource to inform and transform planning of programs such as Open Streets.

#### 3.2.5 Theme 5: train and educate stakeholders

This theme focuses on training and educating stakeholders to promote Open Streets programs, supporting collaboration and capacity building. The Open Street strategy, “Encouraging capacity building increases the quality of the program,” is presented in the results of a study by Díaz Del Castillo et al., as part of the continuation and growth strategies for Recreovía and HEVS, where investment in staff training and better working conditions was essential to ensure skilled instructors who could attract and retain participants (41). Prioritizing human resources and professional development could strengthen program delivery and sustainability (41). This connects to the ERIC strategy “Create a learning collaborative” (sub-theme 5.1), because both emphasize continuous learning; capacity building improves individual and organizational quality, while a learning collaborative fosters a shared knowledge and collective improvement across Open Street programs. The next Open Street strategy, “Implement health education sessions for physical activity, nutrition, and wellness,” is presented in a technical report by the Ministry of Health of Peru in 2015, as a part of the required modules of Ciclovia Recreativa, which include three physical activity modules, one on healthy eating, and one on general health promotion (66). This integration of structured educational content into the program enables participants to engage not only in physical activity but also to learn about healthy lifestyles (66). This relates to the ERIC strategy “Conduct educational outreach visits” (sub-theme 5.2), since both focus on extending health knowledge, and sessions provide on-site learning during the Ciclovia, while outreach visits expand this education into neighborhoods, schools, or workplaces to reinforce the program’s impact.

#### 3.2.6 Theme 6: support clinicians/stakeholders

This theme focuses on supporting stakeholders/practitioners and facilitating collaboration across sectors to promote physical activity. The Open Street strategy, “Strengthen alliances with the public and private sectors,” is presented in the term results of the Recreovía logic model by Ríos et al. emphasizing the role of multi-sector partnerships in sustaining program delivery, expanding resources, and increasing visibility (67). Building collaborative networks with government agencies, companies, and civil society could reinforce program impact and reach (67). This relates to the ERIC strategy “Facilitate relay of data to stakeholders” (sub-theme 6.1), because effective alliances depend on transparent communication, sharing program results and data ensures accountability, maintains trust, and could help partners see the value of continued investment. The next Open Street strategy, “Plan for sustainability and funding by engaging businesses,” is presented as a recommendation for improving Open Street events in an evaluation study by Engelberg et al. of San Diego’s first Open Street initiative (CicloSDias) (22). Actively cultivating business engagement to secure ongoing resources could help sustain the program beyond one-time events (22). This aligns with the ERIC strategy “Develop resource-sharing agreements” (sub-theme 6.2), because both focus on creating structured partnerships, business engagement brings in funding or sponsorship, while resource-sharing agreements formalize contributions (e.g., staff, equipment, services) to ensure sustainability and reduce reliance on a single funding stream (22).

#### 3.2.7 Theme 7: engage consumers

Theme seven focuses on actively involving participants, community members, and diverse groups in Open Streets programs to maximize the co-benefits of the program. The Open Street strategy, “increased awareness among the population about using the structure available for Open Street programs,” is presented in a paper by Parra et al., where the authors emphasize that despite significant investments in Ciclovía, Ciclorutas, and parks, their health impact depends on people knowing about and using them (50). This means that communication, education, and engagement efforts are needed so residents take advantage of these infrastructures for physical activity (50). This relates to the ERIC strategy “Involving participants/family members” (sub-theme 7.1), because raising awareness is more cost-effective when families and communities are directly engaged, creating social support networks that reinforce participation and regular use of the available structures.

The next Open Street strategy, “Active transportation can be socially transmitted and become a norm to promote PA in Open Street programs,” is presented in a study by Ko et al., where the authors note that children and adults in rural ciclovias began adopting bicycling and walking when opportunities and visibility increased, demonstrating how behaviors can be spread through social learning and community modeling (46). When people see others regularly engaging in active transportation, it normalizes the behavior and could encourage broader participation (46). This aligns with the ERIC strategy “Prepare participants/consumers to be active participants” (sub-theme 7.2), as equipping community members with the skills, resources, and confidence can enhance their ability to join in and reinforce these emerging social norms.

#### 3.2.8 Theme 8: utilize financial strategies

This theme emphasizes the use of financial approaches to sustain and scale up Open Streets programs. The Open Street strategy, which states that “programs must reduce and expedite permitting requirements,” is presented in a study by Hipp et al. The authors argue that burdensome and inconsistent permitting processes remain a key barrier to scaling Open Streets in the US. Streamlining or creating dedicated permitting pathways can make it easier, cheaper, and faster for organizers to host events (43). This aligns with the ERIC strategy “Alter incentive/allowance structures” (sub-theme 8.1), because both strategies address systemic barriers, such as reducing permitting obstacles and adjusting incentives to create an enabling environment that encourages municipalities and communities to hold more frequent and sustainable Open Street programs (43). The next Open Street strategy: “Establishing political support from the mayor, city council, and/or other political representatives is important because elected officials most commonly allocate the public resources needed for implementation.” It is presented in the Open Streets Guide developed by the Alliance for Biking and Walking, as a part of the lessons on securing sustainability and legitimacy, emphasizing that high-level political champions are often decisive in dedicating funds, staff, and institutional backing (64). Cultivating visible support from elected officials could improve access to the municipal budgets and resources required to run and expand programs (64). This aligns with the ERIC strategy, “Accessing New Funding” (sub-theme 8.2), as political support can provide access to public resources and position programs to pursue new and diversified funding streams with stronger credibility and institutional endorsement.

#### 3.2.9 Theme 9: change infrastructure

Theme nine focuses on changing infrastructure to promote physical activity through various strategies. The Open Street strategy: “Utilizing indoor and outdoor settings as alternatives to the Open Street program for older adults during public health emergencies.” It is presented in a study by Gónzalez et al., where the authors describe how the Recreovía adapted during the COVID-19 pandemic with virtual sessions, window-based classes, and socially distanced park activities to maintain physical activity opportunities (61). Creating flexible delivery models, particularly for older adults, to enable them to remain active despite lockdowns or social distancing conditions (61). This relates to the ERIC strategy “Changing physical structures and equipment” (sub-theme 9.1), since both focus on adaptation. Providing safe alternative venues complements the modification of physical spaces and tools to ensure continuity of physical activity during public health emergencies (61).

The next Open Street strategy, “Use of static publicity of the programs in widely used spaces” is presented in a technical report by the Ministry of Health of Peru in 2015, as a part of the promotion and communication activities for Ciclovia Recreativa, where municipalities are instructed to place posters and signage in gyms, parks, schools, and transportation terminants to raise awareness and participation (66). This means leveraging visible, everyday community spaces to keep the program in the public eye and encourage consistent engagement (66). This relates to the ERIC strategy, “Start a Dissemination Organization” (sub-theme 9.2), because a dedicated dissemination organization would formalize and expand these efforts, coordinating broader communication campaigns and sustaining outreach across regions.

### 3.3 Assessment of methodological quality of studies

In the studies appraised with the MMAT-Qualitative (section 1) studies (*n* = 11), most demonstrated clear questions, appropriate case-study or interview designs, and clear linkages between data and interpretations. Limitations commonly included small purposive samples, limited reflexivity/positionality reporting, and sparse detail coding inter-reliability, which could constrain transferability. For the MMAT-Quantitative non-randomized (section 3) studies (*n* = 13), validated measures and multivariable analyses were common strengths, while cross-sectional designs, residual confounding, and self-report biases constrained causal inference and external validity. In the MMAT-Quantitative descriptive (section 4) studies (*n* = 20) were the most common, methods fit the event-day description (counts, intercept surveys, SOPARC protocol). However, representativeness was often uncertain due to convenience sampling, single-day data collection, and unreported nonresponse. For MMAT-Mixed methods (section 5) studies (*n* = 8), most provided a clear rationale and triangulated surveys, interviews, and administrative data to yield actionable insights, and treatment of quantitative-qualitative divergences was limited.

Across reviews (*n* = 3) appraised with the AMSTAR-2 tool, multiple critical domains (*a priori* protocol, comprehensive search, duplicate selection/extraction, and risk-of-bias assessment) were frequently unmet, yielding critically low confidence ratings. Even where eligibility criteria and search procedures were described, syntheses were primarily narrative with limited incorporation of study quality into conclusions; findings are best interpreted as contextual rather than effects. For gray-literature items assessed with the AACODS checklist (*n* = 4), Authority and Significance were high (governmental/sector sources with strong implementation detail), and procedures were clearly specified. Objectivity was often partial and coverage context-bounded; accordingly, these sources are used to describe standards, processes, and implementation context, not to infer intervention effectiveness. See Supplementary Table 6 for the methodological appraisal of the studies.

## 4 Discussion

This scoping review identified Open Street strategies in the literature across the Americas and examined their alignment with the ERIC taxonomy. We identified 63 strategies (see Supplementary Table 8) from 59 studies, all of which aligned within the ERIC taxonomy, however, there were approximately 21 ERIC strategies (see Supplementary Table 7) that were not relevant to Open Streets, underscoring that while many ERIC strategies were applicable, not all suited this setting. These findings illustrate that while ERIC provides a strong foundation, adaptations are needed to capture strategies specific to community-based, non-clinical interventions.

Numerous studies have demonstrated that incorporating physical activity is crucial for preventing chronic diseases (7, 8, 21, 26–34, 38, 45, 59). A robust literature exists describing how Open Streets programs promote physical activity, many of which are included in this review. To date, there has been sparse information on Open Streets implementation strategies. The implementation of this program can improve the health of populations, particularly those with the lowest prevalence of physical activity in the Americas (9, 10). We found that Open Street programs consistently foster physical activity and social connectedness across diverse settings, with participants frequently meeting or exceeding PA guidelines during events (17). However, the reach and equity of these programs vary by context. For example, in Bogotá and Santiago de Cali, Ciclovia facilitated cross-neighborhood mobility across socioeconomic divides (17, 18). However, geographic access remained highly unequal, with low-SES residents facing significantly longer distances to travel on these routes (58). In contrast, US programs were smaller in scale and often attracted disproportionately white, higher-income participants in urban areas (35). Conversely, targeted outreach in rural towns was successful in engaging with Hispanic/Latino and American Indian communities (45, 46). These contextual disparities underscore the importance of tailoring strategies to local demographics and built environment conditions.

The strategies we identified clustered around planning promotion, evaluation, and sustainability, with coalition-building emerging as the most common ERIC-aligned strategy. This is consistent with prior literature, fostering multisectoral partnerships (68), political support (48), and community engagement (66) were essential for program adoption and long-term sustainability (18, 41, 46). Strategies unique to Open Streets included applying tactical urbanism to reimagine street use during emergencies (17), leveraging family cohesion to motivate child/adolescent participation (59), and rapidly adapting programs to virtual or balcony formats during the COVID-19 pandemic (61). These innovations illustrate the adaptability of Open Streets to evolving public health challenges (e.g., infectious diseases, climate change, physical inactivity) (15, 69–71).

Despite promising evidence, several gaps remain. First, while strategies were well-documented, few studies have evaluated their effectiveness across contexts or over time. For example, we cannot yet determine which strategies are most effective for reaching underserved groups, sustaining funding, or scaling programs. Second, most studies came from Colombia and the US, limiting generalizability to other parts of the Americas or globally. Additionally, rural contexts remained remarkably underexplored. Third, social inclusion and equity were evident in multiple studies reviewed (17, 62); however, the systematic assessment of implementation mechanisms, such as how strategies reduce inequities in access or outcomes, was not explored. Offering equitable access and inviting the community as a whole to participate in the program is especially important for promoting physical activity and improving public health (48). Participation in the Open Streets should be voluntary and welcoming to all socioeconomic levels, races, ethnicities, abilities, ages, and genders to reduce inequities (41, 58, 72). For example, Open Street strategies such as targeted outreach, partnerships with local organizations (e.g., non-profits, businesses, and local government), and transportation support could help engage low-socioeconomic-status, rural, and indigenous communities (45, 46, 57). Additionally, adapting program activities to cultural preferences and ensuring representation in the planning processes could foster inclusivity and more participation in Open Streets (64, 65). Examples of operationalizing Open Street Strategies with local governments and non-profits include integrating these programs into existing health promotion plans, allocating dedicated funding for equity-focused outreach, and forming cross-sector coalitions to coordinate resources and community engagement (44, 73).

A strength of this review is that it is the first study to identify strategies from Open Streets programs in diverse countries and compare them to the ERIC strategies, exposing several unique comparisons that are useful for the program’s implementation. Some of the Open Street’s strategies obtained from this review carry specific steps for upscaling the program and can provide useful techniques for implementers (see Supplementary Table 7). This is not surprising, given that ERIC was initially developed for healthcare interventions. However, our review indicates that most Open Streets strategies align well with ERIC strategies, particularly those emphasizing coalition-building, context adaptation, stakeholder engagement, and interactive evaluation. These findings highlight that while specific ERIC strategies are not directly transferable to non-clinical, community-based interventions, the taxonomy’s core principles are highly relevant and serve as a valuable foundation for adapting existing taxonomies or frameworks to new settings. Additionally, our use of ERIC highlights the similarities between established implementation strategies in healthcare and those effective in public health initiatives, such as Open Streets. While our review identifies strategies being used, we cannot mention which strategies are most effective in various contexts. This reveals more areas of overlap than differences, suggesting that adapting existing taxonomies, rather than introducing new ones, could be feasible and advantageous.

Future opportunities include testing strategies in controlled or quasi-experimental designs, assessing their transferability across diverse socio-political contexts, and adapting the ERIC strategies to be more explicit for community and public-space interventions. Particular attention should be given to implementation mechanisms in low-resource and culturally diverse settings, ensuring that Open Streets expand access equitably. Programs could also benefit from embedding evaluation processes that track sustainability, costs, and co-benefits such as environmental quality, social cohesion, and local economic activity. There is an excellent opportunity for future studies to continue bridging the gap between the disciplines of physical activity and implementation science, testing, adapting, and scaling up implementation strategies to strengthen the population health impact of Open Streets and similar programs (74–76). While this review concentrated on classifying the identified Open Street strategies to ERIC to highlight areas of alignment and misalignment, this is only the first paper in a planned series of studies. In our follow-up work, we aim to specify and refine the strategies identified in this review, guided by the recommendations of Proctor et al. (19).

There are several limitations of this review; first, not all countries in the Americas were included in the studies or documented these initiatives, which decreases its generalizability to the rest of the world. Second, due to our focus on the Americas only and considering the cultural differences with other parts of the world, modifications may be necessary for implementation. Third, we only included a limited number of gray literature; future studies should systematically examine the gray literature to get a broader context on Open Street programs. Fourth, we decided to focus our search on English, Spanish, and Portuguese; however, future studies should examine if there is gray literature in the indigenous languages of the Americas, particularly in Latin America, which are spoken by millions of individuals (77).

## 5 Conclusion

This scoping review presents the first systematic classification of Open Streets implementation strategies in relation to the ERIC taxonomy of strategies. While substantial alignment exists, Open Streets also introduces unique approaches that highlight the value of adapting healthcare-driven frameworks for community-based health promotion programs. The dissemination and replication of Open Streets programs into other cities and rural areas are essential for public health, both locally and globally, in low and middle-income countries. They are crucial to reducing chronic disease disparities and increasing access to and equity in physical activity. Future research should focus on implementation mechanisms for low-resourced domestic and international settings to inform the most parsimonious and resource-sensitive approaches to Open Streets programs.

## Data Availability

The raw data supporting the conclusions of this article will be made available by the authors, without undue reservation.

## References

[1] Luciani S Nederveen L Martinez R Caixeta R Chavez C Sandoval RC . Noncommunicable diseases in the Americas: a review of the Pan American health organization's 25-year program of work. Rev Panam Salud Publica. (2023) 47:e13. doi: 10.26633/RPSP.2023.13, PMID: 37114168 PMC10128884

[2] Luciani S Agurto I Caixeta R Hennis A. Prioritizing noncommunicable diseases in the Americas region in the era of COVID-19. Rev Panam Salud Publica. (2022) 46:e83. doi: 10.26633/RPSP.2022.83, PMID: 35875322 PMC9299393

[3] Curado MP de Souza DL. Cancer burden in Latin America and the Caribbean. Ann Glob Health. (2014) 80:370–7. doi: 10.1016/j.aogh.2014.09.009, PMID: 25512152

[4] Colditz GA Wei EK. Preventability of Cancer: the relative contributions of biologic and social and physical environmental determinants of Cancer mortality. Annu Rev Public Health. (2012) 33:137–56. doi: 10.1146/annurev-publhealth-031811-124627, PMID: 22224878 PMC3631776

[5] Colditz GA Wolin KY Gehlert S. Applying what we know to accelerate cancer prevention. Sci Transl Med. (2012) 4:127rv4. doi: 10.1126/scitranslmed.3003218 PMC3343638 22461645

[6] American Cancer Society, Institute of Medicine. Fulfilling the potential of cancer prevention and early detection: an American Cancer Society and Institute of Medicine Symposium. Washington, DC: National Academies Press (2004). Available online at: http://www.nap.edu/catalog/10941 25009868

[7] Warburton DER Bredin SSD. Health benefits of physical activity: a systematic review of current systematic reviews. Curr Opin Cardiol. (2017) 32:541–56. doi: 10.1097/HCO.0000000000000437, PMID: 28708630

[8] McTiernan A Friedenreich CM Katzmarzyk PT Powell KE Macko R Buchner D . Physical activity in Cancer prevention and survival: a systematic review. Med Sci Sports Exerc. (2019) 51:1252–61. doi: 10.1249/MSS.0000000000001937, PMID: 31095082 PMC6527123

[9] Guthold R Stevens GA Riley LM Bull FC. Worldwide trends in insufficient physical activity from 2001 to 2016: a pooled analysis of 358 population-based surveys with 1.9 million participants. Lancet glob. Health. (2018) 6:e1077–86. doi: 10.1016/S2214-109X(18)30357-7, PMID: 30193830

[10] Strain T Flaxman S Guthold R Semenova E Cowan M Riley LM . National, regional, and global trends in insufficient physical activity among adults from 2000 to 2022: a pooled analysis of 507 population-based surveys with 5·7 million participants. Lancet Glob Health. (2024) 12:e1232–43. doi: 10.1016/S2214-109X(24)00150-5, PMID: 38942042 PMC11254784

[11] Galaviz KI Harden SM Smith E Blackman KC Berrey LM Mama SK . Physical activity promotion in Latin American populations: a systematic review on issues of internal and external validity. Int J Behav Nutr Phys Act. (2014) 11:77. doi: 10.1186/1479-5868-11-77 24938641 PMC4073811

[12] Zieff SG Musselman EA Sarmiento OL Gonzalez SA Aguilar-Farias N Winter SJ . Talking the walk: perceptions of neighborhood characteristics from users of open streets programs in Latin America and the USA. J Urban Health. (2018) 95:899–912. doi: 10.1007/s11524-018-0262-6, PMID: 29948785 PMC6286281

[13] Ciclovías Recreativas de las Américas. Ciclovías Recreativas de las Américas. (2019). Available online at: https://cicloviasrecreativas.org/

[14] Sá T Garcia L Andrade D. Reflexões sobre os benefícios da integração dos programas Ruas de Lazer e Ciclofaixas de Lazer em São Paulo. Rev Bras Ativ Fis Saude. (2017) 22:5–12. doi: 10.12820/rbafs.v.22n1p5-12

[15] Velázquez-Cortés D Nieuwenhuijsen MJ Jerrett M Rojas-Rueda D. Health benefits of open streets programmes in Latin America: a quantitative health impact assessment. Lancet Planet Health. (2023) 7:e590–9. doi: 10.1016/S2542-5196(23)00109-2, PMID: 37438000

[16] Bird A Díaz del Castillo A Hipp A . Open streets: trends and opportunities. (2017). Available online at: https://www.880cities.org/images/880tools/openstreets-policy-brief-english.pdf

[17] Mejia-Arbelaez C Sarmiento OL Mora Vega R Flores Castillo M Truffello R Martínez L . Social inclusion and physical activity in Ciclovía Recreativa programs in Latin America. Int J Environ Res Public Health. (2021) 18:655. doi: 10.3390/ijerph18020655, PMID: 33466637 PMC7828741

[18] Sarmiento OL Díaz Del Castillo A Triana CA Acevedo MJ Gonzalez SA Pratt M. Reclaiming the streets for people: insights from Ciclovías Recreativas in Latin America. Prev Med. (2017) 103:S34–40. doi: 10.1016/j.ypmed.2016.07.028, PMID: 27497659

[19] Proctor EK Powell BJ McMillen JC. Implementation strategies: recommendations for specifying and reporting. Implement Sci. (2013) 8:139. doi: 10.1186/1748-5908-8-139, PMID: 24289295 PMC3882890

[20] Powell BJ Waltz TJ Chinman MJ Damschroder LJ Smith JL Matthieu MM . A refined compilation of implementation strategies: results from the expert recommendations for implementing change (ERIC) project. Implement Sci. (2015) 10:21. doi: 10.1186/s13012-015-0209-1, PMID: 25889199 PMC4328074

[21] Castro Torres AF Alburez-Gutierrez D. North and south: naming practices and the hidden dimension of global disparities in knowledge production. Proc Natl Acad Sci USA. (2022) 119:e2119373119. doi: 10.1073/pnas.2119373119, PMID: 35238625 PMC8915996

[22] Engelberg JK Carlson JA Black ML Ryan S Sallis JF. Ciclovía participation and impacts in San Diego, CA: the first CicloSDias. Prev Med. (2014) 69:S66–73. doi: 10.1016/j.ypmed.2014.10.005, PMID: 25459488 PMC4313726

[23] Paez DC Reis RS Parra DC Hoehner CM Sarmiento OL Barros M . Bridging the gap between research and practice: an assessment of external validity of community-based physical activity programs in Bogotá, Colombia, and Recife, Brazil. Transl Behav Med. (2015) 5:1–11. doi: 10.1007/s13142-014-0275-y, PMID: 25729448 PMC4332909

[24] Gomez-Feliciano L McCreary LL Sadowsky R Peterson S Hernandez A McElmurry BJ . Active living Logan Square. Am J Prev Med. (2009) 37:S361–7. doi: 10.1016/j.amepre.2009.09.003 19944936

[25] Lawrence RS. Diffusion of the U.S. preventive services task force recommendations into practice. J Gen Intern Med. (1990) 5:S99–S103. doi: 10.1007/BF02600852 2231074

[26] Woolf SH DiGuiseppi CG Atkins D Kamerow DB. Developing evidence-based clinical practice guidelines: lessons learned by the US preventive services task force. Annu Rev Public Health. (1996) 17:511–38. doi: 10.1146/annurev.pu.17.050196.002455, PMID: 8724238

[27] Peters MDJ Marnie C Colquhoun H Garritty CM Hempel S Horsley T . Scoping reviews: reinforcing and advancing the methodology and application. Syst Rev. (2021) 10:263. doi: 10.1186/s13643-021-01821-3, PMID: 34625095 PMC8499488

[28] Tricco AC Lillie E Zarin W O’Brien KK Colquhoun H Levac D . PRISMA extension for scoping reviews (PRISMA-ScR): checklist and explanation. Ann Intern Med. (2018) 169:467–73. doi: 10.7326/M18-0850 30178033

[29] Ouzzani M Hammady H Fedorowicz Z Elmagarmid A. Rayyan—a web and mobile app for systematic reviews. Syst Rev. (2016) 5:210. doi: 10.1186/s13643-016-0384-4, PMID: 27919275 PMC5139140

[30] Fife ST Gossner JD. Deductive qualitative analysis: evaluating, expanding, and refining theory. Int J Qual Methods. (2024) 23:1–12. doi: 10.1177/16094069241244856

[31] Waltz TJ Powell BJ Matthieu MM Damschroder LJ Chinman MJ Smith JL . Use of concept mapping to characterize relationships among implementation strategies and assess their feasibility and importance: results from the expert recommendations for implementing change (ERIC) study. Implement Sci. (2015) 10:109. doi: 10.1186/s13012-015-0295-0, PMID: 26249843 PMC4527340

[32] Hong QN Pluye P Fàbregues S Bartlett G Boardman F Cargo M . Mixed methods appraisal tool (MMAT), version 2018. (2018).

[33] Shea BJ Reeves BC Wells G Thuku M Hamel C Moran J . AMSTAR 2: a critical appraisal tool for systematic reviews that include randomised or non-randomised studies of healthcare interventions, or both. BMJ. (2017) 358:j4008. doi: 10.1136/bmj.j4008 28935701 PMC5833365

[34] Tyndall J. AACODS Checklist Flinders University (2010). Available online at: https://fac.flinders.edu.au/dspace/api/core/bitstreams/e94a96eb-0334-4300-8880-c836d4d9a676/content

[35] Hipp JA Eyler AA Kuhlberg JA. Target population involvement in urban ciclovias: a preliminary evaluation of St. Louis open streets. J Urban Health. (2012) 90:1010–5. doi: 10.1007/s11524-012-9759-6, PMID: 22948790 PMC3853177

[36] Salazar-Collier CL Reininger B Gowen R Rodriguez A Wilkinson A. Evaluation of event physical activity engagement at an open streets initiative within a Texas-Mexico border town. J Phys Act Health. (2018) 15:605–12. doi: 10.1123/jpah.2017-0112, PMID: 29741429

[37] Prada ETP Camargo-Lemos DM Férmino RC. Participation and physical activity in recreovia of Bucaramanga, Colombia. J Phys Act Health. (2021) 18:1277–85. doi: 10.1123/jpah.2021-0047, PMID: 34489368

[38] Rubio MA Triana C King AC Rosas LG Banchoff AW Rubiano O . Engaging citizen scientists to build healthy park environments in Colombia. Health Promot Int. (2021) 36:223–34. doi: 10.1093/heapro/daaa031, PMID: 32361761 PMC7954214

[39] Cervero R Sarmiento OL Jacoby E Gomez LF Neiman A. Influences of built environments on walking and cycling: lessons from Bogotá. Int J Sustain Transp. (2009) 3:203–26. doi: 10.1080/15568310802178314

[40] Teunissen T Sarmiento O Zuidgeest M Brussel M. Mapping equality in access: the case of Bogotá’s sustainable transportation initiatives. Int J Sustain Transp. (2015) 9:457–67. doi: 10.1080/15568318.2013.808388

[41] Díaz Del Castillo A González SA Ríos AP Páez DC Torres A Díaz MP . Start small, dream big: experiences of physical activity in public spaces in Colombia. Prev Med. (2017) 103:S41–50. doi: 10.1016/j.ypmed.2016.08.028, PMID: 27575321

[42] Eyler AA Hipp JA Lokuta J. Moving the barricades to physical activity: a qualitative analysis of open streets initiatives across the United States. Am J Health Promot. (2015) 30:e50–8. doi: 10.4278/ajhp.131212-QUAL-633, PMID: 25162326

[43] Hipp JA Bird A Van Bakergem M Yarnall E. Moving targets: promoting physical activity in public spaces via open streets in the US. Prev Med. (2017) 103:S15–20. doi: 10.1016/j.ypmed.2016.10.014 27773707

[44] Díaz Del Castillo A Sarmiento OL Reis RS Brownson RC. Translating evidence to policy: urban interventions and physical activity promotion in Bogotá, Colombia and Curitiba. Brazil Behav Med Pract Policy Res. (2011) 1:350–60. doi: 10.1007/s13142-011-0038-y, PMID: 24073055 PMC3717654

[45] Perry CK Ko LK Hernandez L Ortiz R Linde S. Ciclovia in a rural Latino community: results and lessons learned. J Public Health Manag Pract. (2017) 23:360–3. doi: 10.1097/PHH.0000000000000555, PMID: 28542020 PMC5785095

[46] Montes F Jaramillo AM Sarmiento OL Ríos AP García L Rubiano O . Community engagement of a rural community in Ciclovía: progressing from research intervention to community adoption. BMC Public Health. (2021) 21:1964. doi: 10.1186/s12889-021-11980-6 34717591 PMC8556949

[47] Rubio MA Guevara-Aladino P Urbano M Cabas S Mejia-Arbelaez C Rodriguez Espinosa P . Innovative participatory evaluation methodologies to assess and sustain multilevel impacts of two community-based physical activity programs for women in Colombia. BMC Public Health. (2022) 22:771. doi: 10.1186/s12889-022-13180-2, PMID: 35428285 PMC9012256

[48] Montes F Sarmiento OL Zarama R Pratt M Wang G Jacoby E . Do health benefits outweigh the costs of mass recreational programs? An economic analysis of four Ciclovía programs. J Urban Health. (2012) 89:153–70. doi: 10.1007/s11524-011-9628-8, PMID: 22170324 PMC3284592

[49] Shu S Batteate C Cole B Froines J Zhu Y. Air quality impacts of a CicLAvia event in downtown Los Angeles, CA. Environ Pollut. (2016) 208:170–6. doi: 10.1016/j.envpol.2015.09.010 26493865

[50] Parra D Gomez L Pratt M Sarmiento OL Mosquera J Triche E. Policy and built environment changes in Bogotá and their importance in health promotion. Indoor Built Environ. (2007) 16:344–8. doi: 10.1177/1420326X07080462

[51] Medina C Romero-Martinez M Bautista-Arredondo S Barquera S Janssen I. Move on bikes program: a community-based physical activity strategy in Mexico City. Int J Environ Res Public Health. (2019) 16:1685. doi: 10.3390/ijerph16101685, PMID: 31091737 PMC6572080

[52] Gómez LF Mosquera J Gómez OL Parra DC Rodríguez DA Sarmiento OL . Social conditions and urban environment associated with participation in the Ciclovia program among adults from Cali, Colombia. Cad Saude Publica. (2015) 31:257–66. doi: 10.1590/0102-311X00086814 26648379

[53] Rodrigues EQ Garcia LMT Ribeiro EHC Barrozo LV Bernal RTI Andrade DR . Use of an elevated avenue for leisure-time physical activity by adults from downtown São Paulo, Brazil. Int J Environ Res Public Health. (2022) 19:5581. doi: 10.3390/ijerph19095581, PMID: 35564976 PMC9106045

[54] De La Peña-de LA Amezcua Núñez JB Hernández-Bonilla A. La promoción de estilos de vida saludable aprovechando los espacios públicos. Horizonte Sanitario. (2017) 16:201–210. doi: 10.19136/hs.a16n3.1878

[55] Cohen D Han B Derose KP Williamson S Paley A Batteate C. CicLAvia: evaluation of participation, physical activity and cost of an open streets event in Los Angeles. Prev Med. (2016) 90:26–33. doi: 10.1016/j.ypmed.2016.06.009, PMID: 27317978 PMC5083970

[56] Wolf SA Grimshaw VE Sacks R Maguire T Matera C Lee KK. The impact of a temporary recurrent street closure on physical activity in new York City. J Urban Health. (2015) 92:230–41. doi: 10.1007/s11524-014-9925-0, PMID: 25575672 PMC4411324

[57] Douglas G Agrawal AW Currin-Percival M Cushing K DeHaan J. Community benefits and lessons for local engagement in a California open streets event: a mixed-methods assessment of Viva CalleSJ 2018. San José, CA: Mineta Transportation Institute (2019).

[58] Parra DC Adlakha D Pinzon JD Van Zandt A Brownson RC Gomez LF. Geographic distribution of the Ciclovia and Recreovia programs by neighborhood SES in Bogotá: how unequal is the geographic access assessed via distance-based measures? J Urban Health. (2021) 98:101–10. doi: 10.1007/s11524-020-00496-w, PMID: 33236318 PMC7873177

[59] Triana CA Sarmiento OL Bravo-Balado A González SA Bolívar MA Lemoine P . Active streets for children: the case of the Bogotá Ciclovía. PLoS One. (2019) 14:e0207791. doi: 10.1371/journal.pone.0207791, PMID: 31091227 PMC6519789

[60] Sarmiento OL Rios AP Paez DC Quijano K Fermino RC. The Recreovía of Bogotá, a community-based physical activity program to promote physical activity among women: baseline results of the natural experiment Al Ritmo de las Comunidades. Int J Environ Res Public Health. (2017) 14:633. doi: 10.3390/ijerph14060633, PMID: 28608844 PMC5486319

[61] González SA Adlakha D Cabas S Sánchez-Franco SC Rubio MA Ossa N . Adaptation of the Recreovía during COVID-19 lockdowns: making physical activity accessible to older adults in Bogotá, Colombia. J Aging Phys Act. (2024) 32:91–106. doi: 10.1123/japa.2022-0236, PMID: 37883645

[62] Torres A Sarmiento OL Stauber C Zarama R. The ciclovia and cicloruta programs: promising interventions to promote physical activity and social capital in Bogotá, Colombia. Am J Public Health. (2013) 103:e23–30. doi: 10.2105/AJPH.2012.301142 PMC3558786 23237179

[63] Slabaugh D Németh J Rigolon A. Open streets for whom?: toward a just livability revolution. J Am Plan Assoc. (2022) 88:253–61. doi: 10.1080/01944363.2021.1955735

[64] Alliance for Biking and Walking. The open streets guide. Available online at: https://bikeleague.org/wp-content/uploads/2023/03/OpenStreetsGuide.pdf

[65] Gómez LF Sarmiento OL Lucumí DI Espinosa G Forero R Bauman A. Prevalence and factors associated with walking and bicycling for transport among young adults in two low-income localities of Bogotá, Colombia. J Phys Act Health. (2005) 2:445–59. doi: 10.1123/jpah.2.4.445

[66] Ministerio de Salud de Perú (2015) Criterios técnicos - implementacion de una ciclovía recreativa

[67] Rios A Paez D Pinzón E Fermino R Sarmiento O. Logic model of the Recreovía: a community program to promote physical activity in Bogota. Rev Bras Ativ Fis Saude. (2017) 22:206–11. doi: 10.12820/rbafs.v.22n2p206-211

[68] Pérez-Escamilla R Vilar-Compte M Rhodes E Safdie M Grajeda R Ibarra L . Implementation of childhood obesity prevention and control policies in the United States and Latin America: lessons for cross-border research and practice. Obes Rev. (2021) 22:e13247. doi: 10.1111/obr.13247 33951275 PMC8365637

[69] Mora C McKenzie T Gaw IM Dean JM von Hammerstein H Knudson TA . Over half of known human pathogenic diseases can be aggravated by climate change. Nat Clim Chang. (2022) 12:869–75. doi: 10.1038/s41558-022-01426-1, PMID: 35968032 PMC9362357

[70] Rocque RJ Beaudoin C Ndjaboue R Cameron L Poirier-Bergeron L Poulin-Rheault RA . Health effects of climate change: an overview of systematic reviews. BMJ Open. (2021) 11:e046333. doi: 10.1136/bmjopen-2020-046333, PMID: 34108165 PMC8191619

[71] Franco Silva M Favarão Leão AL O'Connor Á Hallal PC Ding D Hinckson E . Understanding the relationships between physical activity and climate change: an umbrella review. J Phys Act Health. (2024) 21:1263–75. doi: 10.1123/jpah.2024-0284, PMID: 39389572

[72] Pérez-Escamilla R Lutter CK Rabadan-Diehl C Rubinstein A Calvillo A Corvalán C . Prevention of childhood obesity and food policies in Latin America: from research to practice. Obes Rev. (2017) 18:28–38. doi: 10.1111/obr.12574, PMID: 28741904

[73] Meisel JD Sarmiento OL Montes F Martinez EO Cepeda M Rodríguez DA . Network analysis of Bogotá's Ciclovía Recreativa, a self-organized multisectorial community program to promote physical activity in a middle-income country. Am J Health Promot. (2014) 28:e127–36. doi: 10.4278/ajhp.120912-QUAN-443 23971523 PMC4752846

[74] Wensing M Grol R. Knowledge translation in health: how implementation science could contribute more. BMC Med. (2019) 17:88. doi: 10.1186/s12916-019-1322-9, PMID: 31064388 PMC6505277

[75] Bosch M Van Der Weijden T Wensing M Grol R. Tailoring quality improvement interventions to identified barriers: a multiple case analysis. Eval Clin Pract. (2007) 13:161–8. doi: 10.1111/j.1365-2753.2006.00660.x, PMID: 17378860

[76] Lovero KL Kemp CG Wagenaar BH Giusto A Greene MC Powell BJ . Application of the expert recommendations for implementing change (ERIC) compilation of strategies to health intervention implementation in low- and middle-income countries: a systematic review. Implement Sci. (2023) 18:56. doi: 10.1186/s13012-023-01310-2, PMID: 37904218 PMC10617067

[77] The World Bank. Indigenous Latin America in the twenty-first century. Latin America & Caribbean Region; (2023). Available online at: World Bank website. https://www.worldbank.org/en/region/lac/brief/indigenous-latin-america-in-the-twenty-first-century-brief-report-page

[78] Jaramillo AM Montes F Sarmiento OL Ríos AP Rosas LG Hunter RF . Social cohesion emerging from a community-based physical activity program: a temporal network analysis. Netw Sci. (2021) 9:35–48. doi: 10.1017/nws.2020.31, PMID: 34322275 PMC8315584

[79] Kuhlberg JA Hipp JA Eyler A Chang G. Open streets initiatives in the United States: closed to traffic, open to physical activity. J Phys Act Health. 11:1468–74. doi: 10.1123/jpah.2012-0376, PMID: 24384529

[80] Barradas SC Finck Barboza C Sarmiento OL. Differences between leisure-time physical activity, health-related quality of life and life satisfaction: Al Ritmo de las Comunidades, a natural experiment from Colombia. Glob Health Promot. (2019) 26:5–14. doi: 10.1177/1757975917703303, PMID: 28762871

[81] Sarmiento O Torres A Jacoby E Pratt M Schmid TL Stierling G. The Ciclovía-Recreativa: a mass-recreational program with public health potential. J Phys Act Health. (2010) 7:S163–80. doi: 10.1123/jpah.7.s2.s163, PMID: 20702905

[82] Benavides J Rowland ST Do V Goldsmith J Kioumourtzoglou MA. Unintended impacts of the open streets program on noise complaints in new York City. Environ Res. (2023) 224:115501. doi: 10.1016/j.envres.2023.115501, PMID: 36796610

[83] Zieff SG Kim MS Wilson J Tierney P. A “Ciclovia” in San Francisco: characteristics and physical activity behavior of Sunday streets participants. J Phys Act Health. (2014) 11:249–55. doi: 10.1123/jpah.2011-0290, PMID: 23363639

[84] Bridges CN Prochnow TM Wilkins EC Porter KMP Meyer MRU. Examining the implementation of play streets: a systematic review of the Grey literature. J Public Health Manag Pract. (2020) 26:E1–E10. doi: 10.1097/PHH.0000000000001015, PMID: 31033807 PMC7329138

[85] Suminski RR Jackson-Short C Duckworth N Plautz E Speakman K Landgraf R . Dover Micro open street events: evaluation results and implications for community-based physical activity programming. Front Public Health. (2019) 7:356. doi: 10.3389/fpubh.2019.00356, PMID: 31824914 PMC6883340

[86] Sarmiento OL Schmid TL Parra DC Díaz-del-Castillo A Pinzón JD Pratt M . Quality of life, physical activity, and built environment characteristics among Colombian adults. J Phys Act Health. (2010) 7:S181–95. doi: 10.1123/jpah.7.s2.s181 20702906

[87] Torres A Díaz MP Hayat MJ Lyn R Pratt M Salvo D . Assessing the effect of physical activity classes in public spaces on leisure-time physical activity: “Al Ritmo de las Comunidades” a natural experiment in Bogota, Colombia. Prev Med. (2017) 103:S51–8. doi: 10.1016/j.ypmed.2016.11.005 27847217

[88] Evenson KR Naumann RB Taylor NL LaJeunesse S Combs TS. Mixed method assessment of built environment and policy responses to the COVID-19 pandemic by United States municipalities focusing on walking and bicycling actions. J Transp Health. (2023) 28:101557. doi: 10.1016/j.jth.2022.101557, PMID: 36510600 PMC9729651

[89] Bull FC Al-Ansari SS Biddle S Borodulin K Buman MP Cardon G . World Health Organization 2020 guidelines on physical activity and sedentary behaviour. Br J Sports Med. (2020) 54:1451–62. doi: 10.1136/bjsports-2020-102955, PMID: 33239350 PMC7719906

[90] Hipp JA Eyler AA Zieff SG Samuelson MA. Taking physical activity to the streets: the popularity of Ciclovía and open streets initiatives in the United States. Am J Health Promot. (2014) 28:S114–5. doi: 10.4278/ajhp.28.3s.S114 24380455

[91] Zieff SG Hipp JA Eyler AA Kim MS. Ciclovía initiatives: engaging communities, partners, and policy makers along the route to success. J Public Health Manag Pract. (2013) 19:S74–82. doi: 10.1097/PHH.0b013e3182841982 23529059 PMC4551419

[92] Aguilar-Farias N Miranda-Marquez S Sadarangani KP Chandia-Poblete D Chandia-Poblete R Cristi-Montero C . Results from Chile's 2018 report card on physical activity for children and youth. J Phys Act Health. (2018) 15:S331–2. doi: 10.1123/jpah.2018-0553 30475142

[93] Bertolini L. From “streets for traffic” to “streets for people”: can street experiments transform urban mobility? Transp Rev. (2020) 40:734–53. doi: 10.1080/01441647.2020.1761907

[94] Agrawal AW Nixon H. A survey of Viva CalleSJ participants. San José, CA: Mineta Transportation Institute (2016).

[95] Shediac-Rizkallah MC Bone LR. Planning for the sustainability of community-based health programs: conceptual frameworks and future directions for research, practice and policy. Health Educ Res. (1998) 13:87–108. doi: 10.1093/her/13.1.87 10178339

[96] Scheirer MA Dearing JW. An agenda for research on the sustainability of public health programs. Am J Public Health. (2011) 101:2059–2067. doi: 10.2105/AJPH.2011.300193 21940916 PMC3222409

