# Supplementary material for: Exploring implementation strategies in evidence-based open streets programs for promoting physical activity in the Americas: a scoping review
Source: Front Public Health. 2025 Dec 1;13:1667559. doi: 10.3389/fpubh.2025.1667559 (PMC12704201; doi:10.3389/fpubh.2025.1667559)
Supplement: Supplementary file 1 [file Table_1.docx]

Supplementary Material

# Supplementary Tables

**1.1 Supplementary Table 1.** Complete search strategy.

| **Information Source (Database, Journal, Email, In-press)** | **Description of search strategy (if applicable)** | **Number of total articles or journal issues that met search criteria (where applicable)** |
| --- | --- | --- |
| Pubmed-Medline | **English**  "Exercise"[Mesh] OR ("physical activity") OR ("physical fitness") OR ("cardiorespiratory fitness") OR ("aerobic capacity") OR (walk*) OR ("resistance training") OR (community) OR ("health promotion") OR (sport) OR ("motor activity") OR (sedentary) OR (inactivity) OR ("leisure activities") OR (exercise) OR ("moderate activit*") OR ("vigorous activit*") OR (bicycl*) OR (bike) OR ("active transportation") OR ("non-motorized transportation") AND "Chronic Disease"[Mesh] OR "Neoplasms"[Mesh] OR "chronic disease" OR "neoplasms" OR "prevention" AND "Built Environment"[Mesh:NoExp] OR ("environmental design") OR ("built environment") OR ("urban design") AND ("intervention") OR ("community intervention") OR ("program") OR ("policy") AND ("open streets") OR ("ciclovia") OR ("ciclovias") OR ("recreovia") | 57 articles |
|  | **Spanish**  ("actividad fisica") OR ("aptitud fisica") OR ("aptitud cardiorrespiratoria") OR ("capacidad aerobica") OR (caminar) OR ("entrenamiento de resistencia") OR (comunidad) OR ("promocion de la salud") OR (deporte) OR ("actividad motora") OR (sedentario) OR (inactividad) OR (ejercicio) OR (bicicleta) OR (ciclismo) OR ("transporte activo") AND ("enfermedad cronica") OR (cancer) OR (prevencion) AND ("entorno construido") AND (ciclovia) OR (recreovia) OR (ciclovias) | 0 articles |
| SCOPUS | **English**  TITLE-ABS-KEY("physical activity") OR TITLE-ABS-KEY("cardiorespiratory fitness") OR TITLE-ABS-KEY("aerobic capacity") OR TITLE-ABS-KEY("walk*") OR TITLE-ABS-KEY("resistance training") OR TITLE-ABS-KEY("community") OR TITLE-ABS-KEY("health promotion") OR TITLE-ABS-KEY("sport") OR TITLE-ABS-KEY("motor activity") OR TITLE-ABS-KEY("sedentary") OR TITLE-ABS-KEY("inactivity") OR TITLE-ABS-KEY("leisure activities") OR TITLE-ABS-KEY("exercise") OR TITLE-ABS-KEY("moderate activit*") OR TITLE-ABS-KEY("vigorous activit*") OR TITLE-ABS-KEY("bicycl*") OR TITLE-ABS-KEY("bike") OR TITLE-ABS-KEY("active transportation") OR TITLE-ABS-KEY("chronic disease") OR TITLE-ABS-KEY("cancer") OR TITLE-ABS-KEY("prevention") OR TITLE-ABS-KEY("non-motorized transportation") AND TITLE-ABS-KEY("environmental design") OR TITLE-ABS-KEY("built environment") OR TITLE-ABS-KEY("urban design") AND TITLE-ABS-KEY("open streets") OR ("ciclovia") OR ("ciclovias") AND TITLE-ABS-KEY("intervention") OR TITLE-ABS-KEY("community intervention") OR TITLE-ABS-KEY("program") OR TITLE-ABS-KEY("policy") AND TITLE-ABS-KEY("Latin America") OR TITLE-ABS-KEY("United States") OR TITLE-ABS-KEY("Brazil") OR TITLE-ABS-KEY("Brasil") OR TITLE-ABS-KEY("Colombia") OR TITLE-ABS-KEY("Argentina") OR TITLE-ABS-KEY("Chile") OR TITLE-ABS-KEY("Bolivia") OR TITLE-ABS-KEY("Venezuela") OR TITLE-ABS-KEY("Peru") OR TITLE-ABS-KEY("Mexico") AND PUBYEAR > 2003 | 23 articles |
|  | **Spanish**  TITLE-ABS-KEY("actividad física") OR TITLE-ABS-KEY("aptitud cardiorrespiratoria") OR TITLE-ABS-KEY("capacidad aeróbica") OR TITLE-ABS-KEY("caminar") OR TITLE-ABS-KEY("entrenamiento de resistencia") OR TITLE-ABS-KEY("comunidad") OR TITLE-ABS-KEY("promoción de la salud") OR TITLE-ABS-KEY("deporte") OR TITLE-ABS-KEY("actividad motora") OR TITLE-ABS-KEY("sedentario") OR TITLE-ABS-KEY("inactividad") OR TITLE-ABS-KEY("actividades de ocio") OR TITLE-ABS-KEY("ejercicio") OR TITLE-ABS-KEY("actividad moderada") OR TITLE-ABS-KEY("actividad vigorosa") OR TITLE-ABS-KEY("bicicleta") OR TITLE-ABS-KEY("ciclismo") OR TITLE-ABS-KEY("transporte activo") OR TITLE-ABS-KEY("enfermedad crónica") OR TITLE-ABS-KEY("cáncer") OR TITLE-ABS-KEY("prevención") OR TITLE-ABS-KEY("transporte no motorizado") AND TITLE-ABS-KEY("diseño ambiental") OR TITLE-ABS-KEY("entorno construido") OR TITLE-ABS-KEY("diseño urbano") AND TITLE-ABS-KEY("calles abiertas") OR TITLE-ABS-KEY("ciclovia") OR TITLE-ABS-KEY("ciclovías")  OR TITLE-ABS-KEY(“recreovia”) AND TITLE-ABS-KEY("intervención") OR TITLE-ABS-KEY("intervención comunitaria") OR TITLE-ABS-KEY("programa") OR TITLE-ABS-KEY("política") AND PUBYEAR > 2003 | 0 Articles |
| Web of Science | **English**  TS=(("physical activity") OR ("physical fitness") OR ("cardiorespiratory fitness") OR ("aerobic capacity") OR ("walk*") OR ("resistance training") OR ("community") OR ("health promotion") OR ("sport") OR ("motor activity") OR ("sedentary") OR ("inactivity") OR ("leisure activities") OR ("exercise") OR ("moderate activit*") OR ("vigorous activit*") OR ("bicycl*") OR ("bike") OR ("active transportation") OR ("chronic disease") OR ("cancer") OR ("prevention") OR ("non-motorized transportation")  ) AND TS=(("environmental design") OR ("built environment") OR ("urban design")) AND TS=(("open streets") OR ("ciclovia") OR ("ciclovias") OR ("recreovia")) AND TS=(("intervention") OR ("community intervention") OR ("program") OR ("policy")) AND TS=(("Latin America") OR ("United States") OR ("Brazil") OR ("Brasil") OR ("Colombia") OR ("Argentina") OR ("Chile") OR ("Bolivia") OR ("Venezuela") OR ("Peru") OR ("Mexico")) | 6 Articles |
|  | **Spanish**  TS=(("actividad física") OR ("aptitud física") OR ("aptitud cardiorrespiratoria") OR ("capacidad aeróbica") OR ("caminar") OR ("entrenamiento de resistencia") OR ("comunidad") OR ("promoción de la salud") OR ("deporte") OR ("actividad motora") OR ("sedentario") OR ("inactividad") OR ("actividades de ocio") OR ("actividad en tiempo libre") OR ("ejercicio") OR ("actividad moderada") OR ("actividad vigorosa") OR ("bicicleta") OR ("ciclismo") OR ("transporte activo") OR ("enfermedad crónica") OR ("cáncer") OR ("prevención") OR ("transporte no motorizado")) AND TS=(("diseño ambiental") OR ("entorno construido") OR ("diseño urbano")) AND TS=(("calles abiertas") OR ("ciclovia") OR ("ciclovias") OR ("recreovia")) AND TS=(("intervención") OR ("intervención comunitaria") OR ("programa") OR ("política")) | 0 Articles |
| SCIELO | **Spanish**  ((TS=(("actividad física") OR ("aptitud física") OR ("aptitud cardiorrespiratoria") OR ("capacidad aeróbica") OR ("caminar") OR ("entrenamiento de resistencia") OR ("comunidad") OR ("promoción de la salud") OR ("deporte") OR ("actividad motora") OR ("sedentario") OR ("inactividad") OR ("actividades de ocio") OR ("actividad en tiempo libre") OR ("ejercicio") OR ("actividad moderada") OR ("actividad vigorosa") OR ("bicicleta") OR ("ciclismo") OR ("transporte activo") OR ("enfermedad crónica") OR ("cáncer") OR ("prevención") OR ("transporte no motorizado"))) AND TS=(("diseño ambiental") OR ("entorno construido") OR ("diseño urbano"))) AND TS=(("intervención") OR ("intervención comunitaria") OR ("programa") OR ("política")) | 7 articles |
|  | **Portuguese**  ((((TS=(("atividade física") OR ("aptidão física") OR ("aptidão cardiorrespiratória") OR ("capacidade aeróbica") OR ("caminhar") OR ("treinamento de resistência") OR ("comunidade") OR ("promoção da saúde") OR ("esporte") OR ("atividade motora") OR ("sedentário") OR ("inatividade") OR ("atividades de lazer") OR ("atividade no tempo livre") OR ("exercício") OR ("atividade moderada") OR ("atividade vigorosa") OR ("bicicleta") OR ("ciclismo") OR ("transporte ativo") OR ("doença crônica") OR ("câncer") OR ("prevenção") OR ("transporte não motorizado"))) AND TS=(("projeto ambiental") OR ("ambiente construído") OR ("projeto urbano")))) AND TS=(("ruas abertas") OR ("ciclovias") OR ("ciclovía") OR ("recreovia"))) | 2 articles |
| LILACS | **Spanish**  ("ciclovia") OR ("ciclovias") OR ("recreovia") OR ("calles abiertas") | 27 Articles |
|  | **Portugues**  ("ruas abertas") OR ("ciclovias") OR ("ciclovía") OR ("recreovia")) | 26 Articles |
| Transportation Research Information Services | **English**  (“open streets”) OR (“ciclovia”) OR (“ciclovias”) OR (“recreovia”) | 32 articles |
|  | **Spanish**  (“calles abiertas”) OR (“ciclovias”) OR (“recreovia”) OR (“ciclovía”) | 21 articles |

**1.2 Supplementary Table 2.** Complete data extraction.

| Extraction fields | Response options |
| --- | --- |
| Article Title / Author/ Year | As indicated in text |
| Journal | As indicated in text |
| Study Design | As indicated in text |
| Language | As indicated in text (English, Spanish or Portuguese) |
| Study Setting | As indicated in the text (Urban/Rural) |
| Country/City | As indicated in text (e.g., Bogotá, Colombia) |
| Key Findings | Summarized findings from the studies. |
| Identified Open Street Strategies | Paraphrased from the studies with the location of where it was found. (e.g., Apply tactical urbanism to Open Street programs to promote mobility in times of health emergencies. (Page. 19, Discussion Section) |
| MMAT Appraisal | Overall appraisal after using the MMAT tool (Qualitative, Quantitative, and Mixed Method studies). |
| AACOPS Appraisal | Overall appraisal of grey literature after using the AACOPS checklist. |
| AMSTAR-2 Appraisal | Overall appraisal after using the AMSTAR-2 tool (Review studies). |

**1.3 Supplementary Table 3.** Color Coding for ERIC-Open Streets Strategies

**Themes adapted from the following study:** Waltz, T.J., Powell, B.J., Matthieu, M.M. *et al.* Use of concept mapping to characterize relationships among implementation strategies and assess their feasibility and importance: results from the Expert Recommendations for Implementing Change (ERIC) study. *Implementation Sci* **10**, 109 (2015). <https://doi.org/10.1186/s13012-015-0295-0>

| Theme 1: Use evaluative and iterative strategies |
| --- |
| - Assess for readiness and identify barriers and facilitators - Audit and provide feedback - Purposefully reexamine the implementation - Develop and implement tools for quality monitoring - Develop and organize quality monitoring systems - Develop a formal implementation blueprint - Conduct local need assessment - Stage implementation scale up - Obtain and use patients/consumers and family feedback - Conduct cyclical small tests of change |
| Theme 2: Provide interactive assistance |
| - Facilitation - Provide local technical assistance - Provide clinical supervision - Centralize technical assistance |
| Theme 3: Adapt and tailor to context |
| - Tailor strategies - Promote adaptability - Use data experts - Use data warehousing techniques |
| Theme 4: Develop stakeholder interrelationships |
| - Identify and prepare champions - Organize clinician implementation team meetings - Recruit, designate, and train for leadership - Inform local opinion leaders - Build a coalition - Obtain formal commitments - Identify early adopters - Conduct local consensus discussions - Capture and share local knowledge - Use advisory boards and workgroups - Use an implementation advisor - Model and simulate change - Visit other sites - Involve executive boards - Develop an implementation glossary - Develop academic partnerships - Promote network weaving |
| Theme 5: Train and educate stakeholders |
| - Conduct ongoing training - Provide ongoing consultation - Develop educational materials - Make training dynamic - Distribute educational materials - Use train-the-trainer strategies - Conduct educational meetings - Conduct educational outreach visits - Create a learning collaborative - Shadow other experts - Work with educational institutions |
| Theme 6: Support clinicians |
| - Facilitate relay of clinical data to providers - Remind clinicians - Develop resource sharing agreements - Revise professional roles - Create new clinical teams |
| Theme 7: Engage consumers |
| - Involve patients/consumers and family members - Intervene with patients/consumers to enhance uptake and adherence - Prepare patients/consumers to be active participants - Increase demand - Use mass media |
| Theme 8: Utilize financial strategies |
| - Fund and contract for the clinical innovation - Access new funding - Place innovation on fee for service lists/formularies - Alter incentive/allowance structures - Make billing easier - Alter patient/consumer fees - Use other payment schemes - Develop disincentives - Use capitated payments |
| Theme 9: Change infrastructure |
| - Mandate change - Change record systems - Change physical structure and equipment - Create or change credentialing and/or licensure standards - Change service sites - Change accreditation or membership requirements - Start a dissemination organization - Change liability laws |

| ID | **Open Street Strategies** | Counts |
| --- | --- | --- |
| 1 | Build a coalition | 30 |
| 2 | Capture and share local knowledge | 25 |
| 3 | Conduct local needs assessment | 21 |
| 4 | Involve patients/consumers and family members | 18 |
| 5 | Identify and prepare champions | 16 |
| 6 | Inform local opinion leaders | 16 |
| 7 | Promote network weaving | 14 |
| 8 | Prepare patients/consumers to be active participants | 13 |
| 9 | Promote adaptability | 13 |
| 10 | Create a learning collaborative | 13 |
| 11 | Conduct educational outreach visits | 12 |
| 12 | Alter incentive/allowance structures | 12 |
| 13 | Use mass media | 11 |
| 14 | Intervene with patients/consumers to enhance uptake and adherence | 11 |
| 15 | Tailor strategies | 10 |
| 16 | Develop resource sharing agreements | 10 |
| 17 | Distribute educational materials | 10 |
| 18 | Increase demand | 10 |
| 19 | Assess for readiness and identify barriers and facilitators | 9 |
| 20 | Facilitation | 8 |
| 21 | Conduct local consensus discussions | 8 |
| 22 | Involve executive boards | 8 |
| 23 | Obtain formal commitments | 8 |
| 24 | Develop academic partnerships | 8 |
| 25 | Develop educational materials | 8 |
| 26 | Conduct educational meetings | 7 |
| 27 | Change physical structure and equipment | 7 |
| 28 | Develop and organize quality monitoring systems | 5 |
| 29 | Work with educational institutions | 5 |
| 30 | Audit and provide feedback | 5 |
| 31 | Access new funding | 5 |
| 32 | Conduct ongoing training | 5 |
| 33 | Recruit, designate, and train for leadership | 4 |
| 34 | Use advisory boards and workgroups | 4 |
| 35 | Start a dissemination organization | 4 |
| 36 | Develop and implement tools for quality monitoring | 4 |
| 37 | Use data experts | 3 |
| 38 | Purposely reexamine the implementation | 3 |
| 39 | Mandate change | 3 |
| 40 | Fund and contract for the clinical innovation | 3 |
| 41 | Develop a formal implementation blueprint | 3 |
| 42 | Identify early adopters | 2 |
| 43 | Stage implementation scale up | 2 |
| 44 | Centralize technical assistance | 2 |
| 45 | Make training dynamic | 2 |
| 46 | Facilitate relay of clinical data to providers | 2 |
| 47 | Use an implementation advisor | 1 |
| 48 | Alter patient/consumer fees | 1 |
| 49 | Change service sites | 1 |
| 50 | Create or change credentialing and/or licensure standards | 1 |
| 51 | Provide local technical assistance | 1 |
| 52 | Use other payment schemes | 1 |
| Note: 21 excluded because zero Open Streets Strategies Matched with ERIC. | | |

**1.4 Supplementary Table 4.** Definitions of Themes and Codebook Development

| Themes | Subthemes | Definitions (Powell et al. 2015) |
| --- | --- | --- |
| 1. Use evaluative and iterative strategies | 1.1 Conduct local needs assessment | Collect and analyze data related to the need for the innovation. |
|  | 1.2 Assess for readiness and identify barriers and facilitators | Assess various aspects of an organization to determine its degree of readiness to implement, barriers that may impede implementation, and strengths that can be used in the implementation effort |
|  | 1.3 Audit and provide feedback | Collect and summarize performance data over a specified period and give it to practitioners and administrators to monitor, evaluate, and modify provider behavior. |
|  | 1.4 Develop and implement tools for quality monitoring | Develop, test, and introduce into quality-monitoring systems the right input—the appropriate language, protocols, algorithms, standards, and measures (of processes, patient/consumer outcomes, and implementation outcomes) that are often specific to the innovation being implemented. |
|  | 1.5 Develop and organize quality monitoring systems | Develop and organize systems and procedures that monitor clinical processes and/or outcomes for the purpose of quality assurance and improvement. |
|  | 1.6 Purposely reexamine the implementation | Monitor progress and adjust clinical practices and implementation strategies to continuously improve the quality of care. |
|  | 1.7 Stage implementation scale up | Phase implementation efforts by starting with small pilots or demonstration projects and gradually move to a system wide rollout. |
|  | 1.8 Develop a formal implementation blueprint | Develop a formal implementation blueprint that includes all goals and strategies. The blueprint should include the following: 1) aim/purpose of the implementation; 2) scope of the change (e.g., what organizational units are affected); 3) timeframe and milestones; and 4) appropriate performance/progress measures. Use and update this plan to guide the implementation effort over time. |
| 1. Provide interactive assistance | 2.1 Facilitation | A process of interactive problem solving and support that occurs in a context of a recognized need for improvement and a supportive interpersonal relationship. |
|  | 2.2 Centralize technical assistance | Develop and use a centralized system to deliver technical assistance focused on implementation issues. |
|  | 2.3 Provide local technical assistance | Develop and use a system to deliver technical assistance focused on implementation issues using local personnel. |
| 1. Adapt and tailor to context | 3.1 Promote adaptability | Identify the ways a clinical innovation can be tailored to meet local needs and clarify which elements of the innovation must be maintained to preserve fidelity. |
|  | 3.2 Tailor strategies | Tailor the implementation strategies to address barriers and leverage facilitators that were identified through earlier data collection. |
|  | 3.3 Use data experts | Involve, hire, and/or consult experts to inform management on the use of data generated by implementation efforts. |
| 1. Develop stakeholder interrelationships | 4.1 Build a coalition | Recruit and cultivate relationships with partners in the implementation effort. |
|  | 4.2 Capture and share local knowledge | Capture local knowledge from implementation sites on how implementers and clinicians made something work in their setting and then share it with other sites. |
|  | 4.3 Identify and prepare champions | Identify and prepare individuals who dedicate themselves to supporting, marketing, and driving through an implementation, overcoming indifference or resistance that the intervention may provoke in an organization. |
|  | 4.4 Inform local opinion leaders | Inform providers identified by colleagues as opinion leaders or “educationally influential” about the clinical innovation in the hopes that they will influence colleagues to adopt it. |
|  | 4.5 Promote network weaving | Identify and build on existing high-quality working relationships and networks within and outside the organization, organizational units, teams, etc. to promote information sharing, collaborative problem-solving, and a shared vision/goal related to implementing the innovation. |
|  | 4.6 Conduct local consensus discussions | Include local providers and other stakeholders in discussions that address whether the chosen problem is important and whether the clinical innovation to address it is appropriate. |
|  | 4.7 Involve executive boards | Involve existing governing structures (*e.g.*, boards of directors, medical staff boards of governance) in the implementation effort, including the review of data on implementation processes. |
|  | 4.8 Obtain formal commitments | Obtain written commitments from key partners that state what they will do to implement the innovation. |
|  | 4.9 Develop academic partnerships | Partner with a university or academic unit for the purposes of shared training and bringing research skills to an implementation project. |
|  | 4.10 Recruit, designate, and train for leadership | Recruit, designate, and train leaders for the change effort. |
|  | 4.11 Use advisory boards and workgroups | Create and engage a formal group of multiple kinds of stakeholders to provide input and advice on implementation efforts and to elicit recommendations for improvements. |
|  | 4.12 Identify early adopters | Identify early adopters at the local site to learn from their experiences with the practice innovation. |
|  | 4.13 Use an implementation advisor | Seek guidance from experts in implementation. |
| 1. Train and educate stakeholders | 5.1 Create a learning collaborative | Facilitate the formation of groups of providers or provider organizations and foster a collaborative learning environment to improve implementation of the clinical innovation. |
|  | 5.2 Conduct educational outreach visits | Have a trained person meet with providers in their practice settings to educate providers about the clinical innovation with the intent of changing the provider’s practice. |
|  | 5.3 Distribute educational materials | Distribute educational materials (including guidelines, manuals, and toolkits) in person, by mail, and/or electronically. |
|  | 5.4 Develop educational materials | Develop and format manuals, toolkits, and other supporting materials in ways that make it easier for stakeholders to learn about the innovation and for clinicians to learn how to deliver the clinical innovation. |
|  | 5.5 Conduct educational meetings | Hold meetings targeted toward different stakeholder groups (*e.g.*, providers, administrators, other organizational stakeholders, and community, patient/consumer, and family stakeholders) to teach them about the clinical innovation. |
|  | 5.6 Work with educational institutions | Encourage educational institutions to train clinicians in the innovation |
|  | 5.7 Conduct ongoing training | Plan for and conduct training in the clinical innovation in an ongoing way. |
|  | 5.8 Make Training Dynamic | Vary the information delivery methods to cater to different learning styles and work contexts, and shape the training in the innovation to be interactive. |
| 1. Support Practitioners | 6.1 Facilitate relay of clinical data to providers | Provide as close to real-time data as possible about key measures of process/outcomes using integrated modes/channels of communication in a way that promotes use of the targeted innovation. |
|  | 6.2 Develop resource sharing agreements | Develop partnerships with organizations that have resources needed to implement the innovation. |
| 1. Engage consumers | 7.1 Involve patients/consumers and family members | Engage or include patients/consumers and families in the implementation effort. |
|  | 7.2 Prepare patients/consumers to be active participants | Prepare patients/consumers to be active in their care, to ask questions, and specifically to inquire about care guidelines, the evidence behind clinical decisions, or about available evidence-supported treatments. |
|  | 7.3 Use mass media | Use media to reach large numbers of people to spread the word about the clinical innovation. |
|  | 7.4 Intervene with patients/consumers to enhance uptake and adherence | Develop strategies with patients to encourage and problem solve around adherence. |
|  | 7.5 Increase demand | Attempt to influence the market for the clinical innovation to increase competition intensity and to increase the maturity of the market for the clinical innovation. |
| 1. Utilize financial strategies | 8.1 Alter incentive/allowance structures | Work to incentivize the adoption and implementation of the clinical innovation. |
|  | 8.2 Access new funding | Access new or existing money to facilitate the implementation. |
|  | 8.3 Fund and contract for the clinical innovation | Governments and other payers of services issue requests for proposals to deliver the innovation, use contracting processes to motivate providers to deliver the clinical innovation, and develop new funding formulas that make it more likely that providers will deliver the innovation. |
|  | 8.4 Alter patient/consumer fees | Create fee structures where patients/consumers pay less for preferred treatments (the clinical innovation) and more for less-preferred treatments. |
|  | 8.5 Use other payment schemes | Introduce payment approaches (in a catch-all category). |
| 1. Change infrastructure | 9.1 Change physical structure and equipment | Evaluate current configurations and adapt, as needed, the physical structure and/or equipment (e.g., changing the layout of a room, adding equipment) to best accommodate the targeted innovation. |
|  | 9.2 Start a dissemination organization | Identify or start a separate organization that is responsible for disseminating the clinical innovation. It could be a for-profit or non-profit organization. |
|  | 9.3 Mandate change | Have leadership declare the priority of the innovation and their determination to have it implemented. |
|  | 9.4 Change service sites | Change the location of clinical service sites to increase access. |
|  | 9.5 Create or change credentialing and/or licensure standards | Create an organization that certifies clinicians in the innovation or encourage an existing organization to do so. Change governmental professional certification or licensure requirements to include delivering the innovation. Work to alter continuing education requirements to shape professional practice toward the innovation. |

**1.5 Supplementary Table 5.** Themes and Sub-Themes of Open Streets Strategies with Example Quotes

| Themes | Subthemes | N | Open Streets Strategies | Example Quotes |
| --- | --- | --- | --- | --- |
| Use evaluative and iterative strategies | 1.1 Conduct local needs assessment | 21 | Encourage agenda setting for open streets by BIPOC residents. | *“Encourage agenda setting for open streets by BIPOC residents.”* |
|  | 1.2 Assess for readiness and identify barriers and facilitators | 9 | Apply tactical urbanism to Ciclovia programs to promote mobility during public health emergencies. | *“Creating tools to facilitate planning, implementation, and evaluation of Ciclovía.”* |
|  | 1.3 Audit and provide feedback | 5 | Organizers should critically evaluate and share the successes and failures to improve the next effort. | *“There is a need for implementing systematic and coordinated process evaluations in their national programs.”* |
|  | 1.4 Develop and implement tools for quality monitoring | 4 | A thorough evaluation of programs and identification of barriers, facilitators, and outcomes are key for sustainability. | *“The implementation of both participatory methodologies provided a multidimensional understanding of the programs’ impacts and multisectoral dialogues that fostered efforts to sustain the community‑based PA programs.”* |
|  | 1.5 Develop and organize quality monitoring systems | 5 | Utilizing indoor and outdoor settings as alternatives of the Recreovia program for older adults during public health emergencies. | *“The decision-making process adapted to the regulations in place and the demand for services, and the health sector recommendations based on the progressive evidence about COVID-19 transmission. Activities in the indoor and outdoor settings were alternatives for older adult users of the Recreovia program to continue being physically active during the strictest period of the pandemic regulations.”* |
|  | 1.6 Purposely reexamine the implementation | 3 | The program's evaluation strategy involves studying the distance between Open Streets and socioeconomic status (SES) categorized areas. | *“This study extends the current state of research on Ciclovia and Recreovia programs in Bogotá by examining the geographic distribution and differences in proximity according to the socioeconomic status of the neighborhoods.”* |
|  | 1.7 Stage implementation scale up | 2 | Organizers should critically evaluate and share the successes and failures to improve the next effort. | *“Organizers should critically evaluate and share the successes and failures so that the next effort may be improved.”* |
|  | 1.8 Develop a formal implementation blueprint | 3 | Open street organizers should undertake several concurrent political and logistical planning efforts that successfully move the proposal from concept to implementation. | *“Open street organizers should then undertake a number of concurrent political and logistical planning efforts that move the proposal successfully from concept to implementation.”* |
| Provide interactive assistance | 2.1 Facilitation | 8 | Acquire Municipal Funding and/or Significant In-Kind Support Public authorities should take the lead in dedicating public funds and/or resources. | *"Logistical coordination during the initiative is essential and must be carried out with attention to detail."* |
|  | 2.2 Centralize technical assistance | 2 | Provide technical assistance to BIPOC businesses in adapting their strategies. | *“Provide technical assistance to BIPOC businesses to adapt strategy.”* |
|  | 2.3 Provide local technical assistance | 1 | Provide technical assistance to BIPOC businesses in adapting their strategies. | *“Provide technical assistance to BIPOC businesses to adapt strategy.”* |
| Adapt and tailor to context | 3.1 Promote adaptability | 13 | Budget allocation and accountability improved and scaled up the programs. | *“Coldeportes allocated national funds, and the departments committed to a progressive increase in local funding. This strengthened existing initiatives and created new programs.”* |
|  | 3.2 Tailor strategies | 10 | Consider context to develop culturally appropriate approaches. | *“Develop culturally appropriate strategies for promoting physical activity and active commuting, especially among women.”* |
|  | 3.3 Use data experts | 3 | Utilize public health representatives to stress the program's importance and justification for health and physical activity promotion. | *“Use of the district's media to communicate the start of the program and the diversion plan, including a press conference where the health representative can participate to emphasize the importance of PA for health and justify the program.”* |
| Develop stakeholder interrelationships | 4.1 Build a coalition | 30 | Gather and foster local political support and collaboration. | *“The first necessary component was local political support and collaboration. Support from top city officials and department leads has been identified as a key factor in many community initiatives.”* |
|  | 4.2 Capture and share local knowledge | 25 | Understand and leverage community power. | *“Develop shared understandings of race, privilege, power.”* |
|  | 4.3 Identify and prepare champions | 16 | Understand and leverage community power. | *“Understand and leverage community power.”* |
|  | 4.4 Inform local opinion leaders | 16 | Gather and foster local political support and collaboration. | *“The first necessary component was local political support and collaboration. Support from top city officials and department leads has been identified as a key factor in many community initiatives.”* |
|  | 4.5 Promote network weaving | 14 | Involve community partners, merchants, residents, and city agencies in the implementation process. | *“Operationalize an efficient programming process. Streamline application, select food vendors to comply with goals of program, market scheduled events, and encourage local communities to offer programs.”* |
|  | 4.6 Conduct local consensus discussions | 8 | Gather and foster local political support and collaboration. | *“The first necessary component was local political support and collaboration. Support from top city officials and department leads has been identified as a key factor in many community initiatives.”* |
|  | 4.7 Involve executive boards | 8 | Open street organizers should undertake several concurrent political and logistical planning efforts that successfully move the proposal from concept to implementation. | *“Open streets organizers should build a willing coalition of advocacy, municipal, and/or private-sector supporters.”* |
|  | 4.8 Obtain formal commitments | 8 | Establishing political support from the mayor, city council, and/or other political representatives is important because elected officials most commonly allocate the public resources needed for implementation. | *“Establishing political support from the mayor, city council, and/or other political representatives is important because elected officials most commonly allocate the public resources needed for implementation.”* |
|  | 4.9 Develop academic partnerships | 8 | Multi-sectoral collaboration to encourage co-benefits of the program. | *“Companies, hospitals, schools, universities and civil society organizations take advantage of this space to disseminate educational material, carry out preventive health studies and implement specific campaigns on health and well-being issues.”* |
|  | 4.10 Recruit, designate, and train for leadership | 4 | Encouraging capacity building increased the quality of the program. | *“Both programs emphasized staff training.”* |
|  | 4.11 Use advisory boards and workgroups | 4 | Foster collaboration between international networks and multidisciplinary groups. | *“Evaluating PA community programs and catching-up with practice-based evidence, can be enhanced by the joint work of international networks and multidisciplinary groups.”* |
|  | 4.12 Identify early adopters | 2 | Plan for sustainability and funding by engaging businesses. | *“Increasing targeted outreach through partnerships with ethnic-specific media outlets, more promotion in stores and clinics in disadvantaged neighborhoods, promoting event attendance through partnerships with employers of highly diverse employees, and engaging ongoing advisors from underserved communities.”* |
|  | 4.13 Use an implementation advisor | 1 | Partner with academic institutions or organizations that regularly implement evaluations to assess open streets. | *“Because each community and initiative is unique, there is a need to tailor the definition of what organizers see as success. Once defined, efforts should be planned and implemented to measure success based on the resources available. Partnering with universities or organizations that regularly implement evaluations may help ease the burden.”* |
| Train and educate stakeholders | 5.1 Create a learning collaborative | 13 | Capacity building increased the quality of the program. | *“Both programs emphasized staff training.”* |
|  | 5.2 Conduct educational outreach visits | 12 | Implement health education sessions for physical activity, nutrition, and wellness. | *“Five modules installed: three on PA promotion, one on healthy eating, and one on health promotion.”* |
|  | 5.3 Distribute educational materials | 10 | Direct communication and marketing efforts toward city residents who experience health disparities. | *“This gap between participants and populations facing disparities presents an opportunity for directed communication and marketing efforts to increase participation by city residents and those facing health disparities.”* |
|  | 5.4 Develop educational materials | 8 | PA instructors play an important role in PA promotion through social media. | *“Physical activity instructors are crucial actors to promote collaborative behaviors, and to be role models in the promotion and dissemination of the Recreovia program.”* |
|  | 5.5 Conduct educational meetings | 7 | Increase awareness among the population about using the structure available for Open Street programs. | *“Increasing awareness and use of environmental resources that are already available in the community may be a cost-effective strategy to increase physical activity and health of the population.”* |
|  | 5.6 Work with educational institutions | 5 | Strengthen alliances with the public and private sectors. | *"Strengthen alliances with the public and private sectors."* |
|  | 5.7 Conduct ongoing training | 5 | Capacity building increased the quality of the program. | *“Both programs emphasized staff training.”* |
|  | 5.8 Make Training Dynamic | 2 | Allow flexibility of the program to adjust to changes in budget and increase participation. | *"Flexibility permitted the program to adjust to changes in budget and increased participation."* |
| Support Practitioners | 6.1 Facilitate relay of clinical data to providers | 2 | Strengthen alliances with the public and private sectors. | *"Strengthen alliances with the public and private sectors."* |
|  | 6.2 Develop resource sharing agreements | 10 | Plan for sustainability and funding by engaging businesses. | *“Increasing targeted outreach through partnerships with ethnic-specific media outlets, more promotion in stores and clinics in disadvantaged neighborhoods, promoting event attendance through partnerships with employers of highly diverse employees, and engaging ongoing advisors from underserved communities.”* |
| Engage consumers | 7.1 Involve patients/consumers and family members | 18 | Increase awareness among the population about using the structure available for Open Street programs. | *“Increasing awareness and use of environmental resources that are already available in the community may be a cost-effective strategy to increase physical activity and health of the population.”* |
|  | 7.2 Prepare patients/consumers to be active participants | 13 | Active transportation can be socially transmitted and become a norm to promote PA in Open Street programs. | *“Community norms around active transportation can be created by increasing awareness and accessibility for active transportation.”* |
|  | 7.3 Use mass media | 11 | Promote the intervention through media. | *“Promotion of event is necessary for success. Market through a variety of media and include a line item in the budget for paid media. Day of wayfinding (connected bus routes, metro stops, and where to park) and signage and maps posted throughout the route indicating route, activities and facilities are critically important.”* |
|  | 7.4 Intervene with patients/consumers to enhance uptake and adherence | 11 | Encourage agenda setting for open streets by BIPOC residents. | *Encourage agenda setting for open streets by BIPOC residents.* |
|  | 7.5 Increase demand | 10 | Increasing open street programs’ reach, capacity, and frequency to lower costs. | *“The enthusiastic response suggests that there is good reason to expand the event to longer routes, to increase the reach and capacity of the event, as well as to hold them more frequently. Even though the event needs government support and is relatively expensive, it is a civic activity that has health benefits to participants.”* |
| Utilize financial strategies | 8.1 Alter incentive/allowance structures | 12 | Programs must reduce and expedite permitting requirements. | *“Coldeportes allocated national funds, and the departments committed to a progressive increase in local funding. This strengthened existing initiatives and created new programs.”* |
|  | 8.2 Access new funding | 5 | Acquire Municipal Funding and/or Significant In-Kind Support Public authorities should take the lead in dedicating public funds and/or resources. | *"Logistical coordination during the initiative is essential and must be carried out with attention to detail."* |
|  | 8.3 Fund and contract for the clinical innovation | 3 | With political support, other countries can benefit from road closures and existing infrastructure to promote PA. | *“The presence of road networks and their relative underutilization during certain hours suggests that with appropriate multisectoral partnerships, political support, and effective management and promotion, many more cities can support Ciclovías on the scale of Bogotá.”* |
|  | 8.4 Alter patient/consumer fees | 1 | Open street programs should build a consistent brand and visibility to provide identity and encourage repeat participation and sponsorship. | *“Build a consistent brand and visibility to provide identity and encourage repeat participation and sponsorship.”* |
|  | 8.5 Use other payment schemes | 1 | Plan for sustainability and funding by engaging businesses. | *“Increasing targeted outreach through partnerships with ethnic-specific media outlets, more promotion in stores and clinics in disadvantaged neighborhoods, promoting event attendance through partnerships with employers of highly diverse employees, and engaging ongoing advisors from underserved communities.”* |
| \Change infrastructure | 9.1 Change physical structure and equipment | 7 | Utilizing indoor and outdoor settings as alternatives to the Open Street program for older adults during public health emergencies. | *“The decision-making process adapted to the regulations in place and the demand for services, and the health sector recommendations based on the progressive evidence about COVID-19 transmission. Activities in the indoor and outdoor settings were alternatives for older adult users of the Recreovia program to continue being physically active during the strictest period of the pandemic regulations.”* |
|  | 9.2 Start a dissemination organization | 4 | Use of static publicity of the programs in widely used spaces. | *“Use of static publicity in gyms, parks, malls, schools, land terminals, highlighting the importance of PA and healthy eating.”* |
|  | 9.3 Mandate change | 3 | Create a policy to site open streets near schools/ community centers. | *“Create a policy to site open streets near schools/ community centers.”* |
|  | 9.4 Change service sites | 1 | Create a policy to site open streets near schools/ community centers. | *“Create policy to site open streets near schools/ community centers.”* |
|  | 9.5 Create or change credentialing and/or licensure standards | 1 | Capacity building increased the quality of the program. | *“Both programs emphasized staff training.”* |

**1.6 Supplementary Table 6.** Assessment of Methodological Quality of Studies

| **ID** | **Tool** | **Article** | **Criterion** | **Assessment** | **Notes** | **Overall Appraisal** |
| --- | --- | --- | --- | --- | --- | --- |
| 1.0 | MMAT | Social Inclusion and Physical Activity in Ciclovía Recreativa Programs in Latin America | 4.1. Is the sampling strategy relevant to address the research question? | Yes | Sampling used surveys of participants across multiple Ciclovía routes in four cities (2015–2019), aligned with aim of studying inclusion and PA. | The study is methodologically solid for a descriptive cross-sectional design. Its main limitations are representativeness of the sample and uncertainty about nonresponse bias, which should be noted when interpreting the generalizability of findings. |
|  |  |  | 4.2. Is the sample representative of the target population? | Partial / Can’t tell | Participants were volunteers in Ciclovía surveys; representativeness of the broader population (or all attendees) may be limited. |  |
|  |  |  | 4.3. Are the measurements appropriate? | Yes | Standardized questionnaires assessed PA, health perception, and sociodemographics; validated PA recommendations considered. |  |
|  |  |  | 4.4. Is the risk of nonresponse bias low? | Can’t tell | Nonresponse bias not fully described; unknown if respondents differ systematically from non-respondents. |  |
|  |  |  | 4.5. Is the statistical analysis appropriate to answer the research question? | Yes | Analyses compared PA outcomes, mobility patterns, and demographics across cities; methods consistent with study design. |  |
| 2.0 | MMAT | Engaging citizen scientists to build healthy park environments in Colombia | 5.1. Adequate rationale for using a mixed methods design? | Yes | The study explicitly used an explanatory sequential mixed-methods design (surveys + accelerometry + participatory citizen science) to capture both behavioral patterns and community perspectives. | The study shows strong methodological quality in its use of mixed methods. Its main limitation is a somewhat limited discussion of divergences between quantitative and qualitative findings, but integration overall was effective and justified. |
|  |  |  | 5.2. Are the different components of the study effectively integrated? | Yes | Quantitative park/PA data were complemented with qualitative citizen scientist observations, and findings were combined to identify barriers/enablers and advocate for policy action. |  |
|  |  |  | 5.3. Are the outputs of the integration adequately interpreted? | Yes | The study connected objective data with community narratives, showing how empirical findings informed advocacy and program changes. |  |
|  |  |  | 5.4. Are divergences/inconsistencies between quantitative and qualitative results adequately addressed? | Partial | While both strands generally aligned, explicit discussion of divergences (if any) was limited; integration was mostly used to strengthen convergent findings. |  |
|  |  |  | 5.5. Do the different components meet the quality criteria of each tradition (qual/quant)? | Yes | Quantitative measures (accelerometry, surveys) and qualitative methods (Our Voice participatory model) were appropriate and described with sufficient rigor. |  |
| 3.0 | MMAT | Active streets for children: The case of the Bogota´ Ciclovía | 4.1. Is the sampling strategy relevant to address the research question? | Yes | Data came from ISCOLE and MARA studies, using structured school-based recruitment across Bogotá; sampling aligns with aim of examining children’s PA in relation to Ciclovía participation. | The study is methodologically robust, especially in measurement and statistical analysis. The main limitations relate to representativeness (restricted to urban schoolchildren) and unclear nonresponse bias, which may limit generalizability. |
|  |  |  | 4.2. Is the sample representative of the target population? | Partial | The sample includes urban schoolchildren (9–13 years) from Bogotá, but may not fully represent all children (e.g., rural, out-of-school, older/younger). |  |
|  |  |  | 4.3. Are the measurements appropriate? | Yes | Accelerometers used for MVPA/SED and standard anthropometry for BMI; validated and reliable measures. |  |
|  |  |  | 4.4. Is the risk of nonresponse bias low? | Can’t tell | Limited detail on recruitment refusals or dropout patterns, making it unclear if respondents differ systematically from non-respondents. |  |
|  |  |  | 4.5. Is the statistical analysis appropriate to answer the research question? | Yes | Used comparisons across Ciclovía participation groups; analyses addressed PA/SED and BMI outcomes appropriately. |  |
| 4.0 | MMAT | Ciclovia in a Rural Latino Community: Results and Lessons | 4.1. Is the sampling strategy relevant to address the research question? | Yes | Used direct observations and intercept surveys aligned with the aim of evaluating event participation and activity. | The study is methodologically adequate for a descriptive pilot event evaluation. Main limitations relate to the small survey sample and uncertainty about nonresponse bias, which constrain generalizability. |
|  |  |  | 4.2. Is the sample representative of the target population? | Partial | The sample (38 survey respondents, mainly women/Latinos) may not fully represent the broader rural community; participation limited to event attendees. |  |
|  |  |  | 4.3. Are the measurements appropriate? | Yes | Observational counts and standardized surveys (in English and Spanish) captured relevant outcomes (attendance, activity type, demographics). |  |
|  |  |  | 4.4. Is the risk of nonresponse bias low? | Can’t tell | Small survey sample; nonrespondent characteristics not described. Risk of bias possible. |  |
|  |  |  | 4.5. Is the statistical analysis appropriate to answer the research question? | Yes | Simple descriptive analysis was appropriate for reporting attendance, activity patterns, and survey responses. |  |
| 5.0 | MMAT | Social cohesion emerging from a community-based physical  activity program: A temporal network analysis | 4.1. Is the sampling strategy relevant to address the research question? | Yes | Facebook network data included all public Recreovía interactions; participant surveys targeted active program users. | The study is methodologically strong in its quantitative analysis of social networks and descriptive surveys. Limitations include the small survey sample size and potential biases in representativeness (both online data and surveys). |
|  |  |  | 4.2. Is the sample representative of the target population? | Partial | Facebook network captures online interactions (which may not represent offline participants equally); surveys limited to 50 participants, not representative of the full user base. |  |
|  |  |  | 4.3. Are the measurements appropriate? | Yes | Network metrics (nodes, edges, super-linear growth) and standardized survey items were appropriate to assess social cohesion and engagement. |  |
|  |  |  | 4.4. Is the risk of nonresponse bias low? | Can’t tell | Survey recruitment/response process not fully detailed; possible selection bias. |  |
|  |  |  | 4.5. Is the statistical analysis appropriate to answer the research question? | Yes | Applied temporal network analysis and descriptive statistics; interpretations consistent with methods. |  |
| 6.0 | MMAT | Participation and engagement of a rural community in Ciclovía: progressing from research intervention to community adoption | 4.1. Is the sampling strategy relevant to address the research question? | Yes | Attendance counts and intercept surveys were systematically conducted at each Ciclovía year to capture trends in participation and engagement. | The study maintains adequate methodological quality, with strong alignment between methods and objectives. However, nonresponse/response bias is a significant limitation, as noted by the authors themselves, and this affects the representativeness of survey findings. |
|  |  |  | 4.2. Is the sample representative of the target population? | Partial | The surveys reflected event attendees, not the entire rural community; participation grew, but generalizability to all residents remains limited. |  |
|  |  |  | 4.3. Are the measurements appropriate? | Yes | Used direct observation counts, standardized intercept surveys, and documentation of planning processes — appropriate for assessing attendance and engagement. |  |
|  |  |  | 4.4. Is the risk of nonresponse bias low? | Can’t tell | Survey sample sizes were relatively small; unclear how respondents compared with non-respondents or non-attendees. |  |
|  |  |  | 4.5. Is the statistical analysis appropriate to answer the research question? | Yes | Analyses tracked changes in attendance and demographics across years; descriptive approach suited to the study’s aims. |  |
| 7.0 | MMAT | Geographic Distribution of the Ciclovia and Recreovia Programs by Neighborhood SES in Bogotá: How Unequal is the Geographic Access Assessed Via Distance-based Measures? | 4.1. Sampling strategy relevant to the research question? | Yes | The study analyzes all residential/mixed-use blocks citywide, computing distance from each block centroid to the nearest Ciclovía/Recreovía access point—directly aligned with assessing geographic access by SES. | Strong methodological fit for a citywide spatial assessment. Main caveats are measurement scope (centroid-to-access distance; other access dimensions like connectivity or transit not modeled), which the authors explicitly acknowledge. |
|  |  |  | 4.2. Sample representative of the target population? | Yes | The unit of analysis is the full set of city blocks across all neighborhoods (stratified by SES), so the spatial “sample” is essentially complete for Bogotá. |  |
|  |  |  | 4.3. Measurements appropriate? | Yes | Uses distance-based measures from block centroids to nearest program access point and reports SES-stratified medians; approach matches the study question (with limitations acknowledged). |  |
|  |  |  | 4.4. Risk of nonresponse bias low? | Not applicable | No human respondents; analysis is based on spatial units (blocks), so nonresponse bias does not apply. (Authors instead discuss measurement/construct limitations, not response bias.) |  |
|  |  |  | 4.5. Statistical analysis appropriate? | Yes | SES differences are reported with nonparametric tests (p < 0.001) and clear descriptive statistics (medians); analytic approach fits the design and data. |  |
| 8.0 | MMAT | Open Streets Initiatives in the United States: Closed to Traffic, Open to Physical Activity | 4.1. Is the sampling strategy relevant to address the research question? | Yes | Authors systematically identified U.S. Open Streets initiatives held in 2011 via internet searches, databases, social media, and personal contacts, then extracted standardized descriptors to compare sites—aligned with their aim to describe initiatives nationally. | Methodologically appropriate for a national descriptive overview. Key limitation is uncertain representativeness/completeness due to reliance on publicly available information and variable reporting (e.g., attendance and evaluations reported for a minority of events). |
|  |  |  | 4.2. Is the sample representative of the target population? | Can’t tell | The compilation yields 47 cities in 2011, but completeness is uncertain (no formal sampling frame; relies on public information and contacts). Reporting gaps (e.g., attendance available for only ~36% of events) underscore uncertainty about coverage. |  |
|  |  |  | 4.3. Are the measurements appropriate? | Yes | Extracted frequency, route length, attendance, evaluation procedures, and host-city sociodemographics—appropriate variables for describing initiatives and enabling cross-city comparisons. |  |
|  |  |  | 4.4. Is the risk of nonresponse bias low? | Can’t tell | No human respondents (catalogue of events); nonresponse bias isn’t directly applicable. Data completeness is the main risk (some variables missing across events). |  |
|  |  |  | 4.5. Is the statistical analysis appropriate to answer the research question? | Yes | Descriptive statistics (counts, means/medians for route length, duration; frequencies for timing/recurrence; attendance ranges) fit the descriptive design and reported data. |  |
| 9.0 | MMAT | The Ciclovia and Cicloruta Programs: Promising Interventions to Promote Physical Activity and Social Capital in Bogota´, Colombia | 4.1. Is the sampling strategy relevant to address the research question? | Yes | Two intercept surveys were conducted (Oct 2009): 1,000 Ciclovía participants sampled at 16 equidistant interception points over three Sundays (every third adult), and 1,000 Cicloruta cyclists sampled at 10 weekday interception points across 5 zones covering all SES strata (every third adult). This aligns with the aim to compare program users citywide. | Methodologically appropriate for a cross-sectional comparison of two citywide programs. Strengths include large samples with broad spatial coverage and validated measures. Main limitations are uncertain representativeness and unknown nonresponse bias, which temper generalizability. |
|  |  |  | 4.2. Is the sample representative of the target population? | Partial / Can’t tell | Coverage across the full circuit/zones and SES strata improves coverage, but intercept convenience sampling of passersby may not fully represent all users (e.g., different times/routes) and no weighting for non-coverage is reported. |  |
|  |  |  | 4.3. Are the measurements appropriate? | Yes | PA assessed with IPAQ (domain-specific; LTPA for Ciclovía, transport cycling for Cicloruta) and classification vs PA guidelines; social capital measured via adapted, previously validated questionnaires; safety perceptions captured via items. These match the constructs of interest. |  |
|  |  |  | 4.4. Is the risk of nonresponse bias low? | Can’t tell | The intercept approach implies some approached individuals may have declined; response rates or comparisons of respondents vs. nonrespondents are not reported, so nonresponse bias cannot be ruled out. (Methods describe who was surveyed but not refusals/response proportions.) |  |
|  |  |  | 4.5. Is the statistical analysis appropriate to answer the research question? | Yes | The paper reports adjusted odds ratios (e.g., Table 4) and clear descriptive comparisons of key outcomes (meeting PA recommendations, safety perceptions, SC), which are appropriate for the cross-sectional design. |  |
| 10.0 | MMAT | Reclaiming the streets for people: Insights from Ciclovías Recreativas in Latin America | 5.1. Adequate rationale for using mixed methods? | Yes | The study set two aims (describe programs via survey; analyze sustainability/scaling via case studies) and used a mixed-methods convergent parallel design applied concurrently with equal priority to address them. | Strong mixed-methods design with clear rationale, rigorous quantitative and qualitative components, and integrated interpretation of sustainability. The main gap is the lack of explicit treatment of quantitative–qualitative divergences, which slightly limits triangulation strength. |
|  |  |  | 5.2. Effective integration of components? | Yes | Quantitative survey results (67 programs; characteristics, inclusion, frequency) are presented alongside five qualitative case studies, with integrated interpretation of sustainability factors (Table/Fig networks). |  |
|  |  |  | 5.3. Outputs of integration adequately interpreted? | Yes | Discussion explicitly reflects on how the mixed methods characterize heterogeneity and sustainability drivers across settings; the qualitative analysis complements prior work focused on Bogotá. |  |
|  |  |  | 5.4. Divergences/inconsistencies addressed? | Can’t tell / Partial | Authors discuss limitations (e.g., 67 coordinator responses; interviews limited to coordinators) but do not explicitly analyze divergences between survey findings and case-study narratives. |  |
|  |  |  | 5.5. Components meet quality criteria of each tradition? | Yes | Quant: Defined sampling frame (CRA registry), online questionnaire with expert-developed items, typology & chi-square tests. Qual: Purposive sampling of 5 cities; semi-structured interviews, audio-recorded & transcribed; policy-document review; dual independent analysis; coordinators rated factor importance (Likert); IRB approval. |  |
| 11.0 | MMAT | Start Small, Dream Big: Experiences of Physical Activity in Public Spaces in Colombia. | 5.1. Adequate rationale for using mixed methods? | Yes | The study explicitly states a mixed-methods approach (semi-structured interviews, document review, and analysis of program data) to identify factors for sustainability and scale-up; authors also ground the choice in established sustainability frameworks. | Overall, this is a well-executed convergent mixed-methods evaluation that triangulates interviews, document review, and program metrics to explain sustainability and scale-up of public-space physical activity initiatives, though limited treatment of quant–qual divergences and context-bound sampling temper generalizability. |
|  |  |  | 5.2. Effective integration of components? | Yes | Authors used a convergent mixed-methods design and cross-compared qualitative themes with quantitative program indicators (e.g., trajectories of hubs, participants, budget) to explain challenges/strategies. |  |
|  |  |  | 5.3. Outputs of integration adequately interpreted? | Yes | The results synthesize qualitative categories (e.g., flexibility, champions) with descriptive trends to interpret how adaptations supported continuation and growth. |  |
|  |  |  | 5.4. Divergences/inconsistencies addressed? | Cant tell | While components are integrated, the paper does not explicitly discuss disagreements or inconsistencies between qualitative and quantitative findings. (No clear statement located.) |  |
|  |  |  | 5.5. Components meet quality criteria of each tradition? | Yes | Qualitative rigor: snowball sampling to information redundancy, independent coding by multiple analysts; ethics approved. Quantitative rigor: defined program indicators and analytic procedures. |  |
| 12.0 | MMAT | The Recreovía of Bogotá, a Community-Based Physical Activity Program to Promote Physical Activity among Women: Baseline Results of the Natural Experiment Al Ritmo de las Comunidades | 4.1. Is the sampling strategy relevant to address the research question? | Yes | Parks were grouped as: (1) future-Recreovía (n=3), (2) controls (n=3) matched on park type, SES, and PA target areas, and (3) existing-Recreovía (n=3). SOPARC observations followed a structured schedule across multiple time blocks—appropriate for comparing park use/PA by program status. | Strong fit between design, measures, and analysis for a baseline, cross-sectional comparison of parks with/without Recreovía. Main caveat is limited representativeness due to purposeful park selection and small N of parks. Findings—e.g., higher female presence and MVPA in Recreovía parks—are supported by the observational protocol and analyses. |
|  |  |  | 4.2. Is the sample representative of the target population? | Partial | Park selection for “future” sites was program-driven (community requests + neighborhood SES), existing sites included very high/very low attendance and the oldest hub; only nine parks total. This purposeful selection supports comparison but may not represent all Bogotá parks. |  |
|  |  |  | 4.3. Are the measurements appropriate? | Yes | Used SOPARC (validated observational tool) to classify users by gender/age/PA level; observers were trained and inter-observer agreement/reliability documented. Measures align with park use and MVPA outcomes. |  |
|  |  |  | 4.4. Is the risk of nonresponse bias low? | Not applicable | No human survey respondents; outcomes derived from systematic observations of park users (SOPARC). Nonresponse bias doesn’t apply here. |  |
|  |  |  | 4.5. Is the statistical analysis appropriate to answer the research question? | Yes | Descriptives plus multilevel multiple regression (intervention group, park type, neighborhood SES → number of visitors, sedentary, MVPA). Fits the design and park data. |  |
| 13.0 | MMAT | Social conditions and urban environment  associated with participation in the Ciclovia  program among adults from Cali, Colombia | 4.1. Sampling strategy relevant to the research question? | Yes | Four-stage probabilistic sampling of adults 18–44 across 67 neighborhoods, with random selection at neighborhood, block, household, and adult levels—appropriate for citywide correlates of Ciclovía participation. | Solid citywide, probability-based design with appropriate measures and multilevel modeling. Main limitation is nonresponse bias/uneven response rates, which tempers generalizability of participation estimates by SES. |
|  |  |  | 4.2. Sample representative of the target population? | Partial | Probability design supports representativeness, but the overall response rate was 66.9% and lower in upper-middle-class neighborhoods (48%), which could skew estimates. |  |
|  |  |  | 4.3. Measurements appropriate? | Yes | Participation measured as “in the last 4 weekends… yes/no”; social conditions captured (sex, age, education, etc.); neighborhood GIS indicators (e.g., presence/length of Ciclovía corridors, parks, bike paths, traffic fatalities). All align with the study aims. |  |
|  |  |  | 4.4. Risk of nonresponse bias low? | No | Authors report response bias concerns: response rate 66.9% overall and substantially lower in upper-middle neighborhoods, indicating nonresponse bias may be present. |  |
|  |  |  | 4.5. Statistical analysis appropriate to answer the question? | Yes | Used multilevel logistic regression with adjusted models for social and environmental covariates; results presented with PORs and 95% CIs (e.g., male sex POR = 1.85; Ciclovía corridor presence POR = 4.27; traffic fatalities POR = 0.45). |  |
| 14.0 | MMAT | Differences between leisure-time physical activity, health-related quality of life and life satisfaction: Al Ritmo de las Comunidades, a natural experiment from Colombia | 4.1. Sampling strategy relevant to the research question? | Yes | Baseline cross-sectional survey of 1,533 adults across nine parks (Recreovía, control, and “future” sites). Participants were selected using structured field procedures in parks (every 4th person in target areas), nearby households (every 4th house within 500 m), and community groups—appropriate to compare Recreovía vs non-Recreovía participants. | Methodologically suitable for a baseline, cross-sectional comparison of PA ↔ HRQoL/LS and Recreovía vs non-Recreovía participants. Strengths are the clear sampling procedures across multiple settings and validated instruments; the main limitation is uncertain representativeness/nonresponse bias due to the absence of response-rate reporting. |
|  |  |  | 4.2. Sample representative of the target population? | Partial / Can’t tell | Coverage from parks/households/community groups improves breadth, but procedures are not a citywide probability sample and no weighting/coverage adjustment is reported; representativeness of all Bogotá adults is uncertain. |  |
|  |  |  | 4.3. Measurements appropriate? | Yes | HRQoL measured with EORTC QLQ-30 (reliability reported for Colombia); Life Satisfaction with the Questions on Life Satisfaction scale; leisure-time PA with IPAQ (long)—all aligned with study aims. |  |
|  |  |  | 4.4. Risk of nonresponse bias low? | Can’t tell | Fieldwork and training are described, but response/participation rates and nonrespondent comparisons are not reported, so nonresponse bias cannot be ruled out. |  |
|  |  |  | 4.5. Statistical analysis appropriate to answer the question? | Yes | Analyses used group comparisons (t-tests) for LS/HRQoL across PA levels and Recreovía status, consistent with the descriptive cross-sectional design. |  |
| 15.0 | AMSTAR-2 | The Ciclovía-Recreativa: A Mass-Recreational Program With Public Health Potential | 1. Research question & inclusion criteria include PICO? | Yes | Population (cities/programs) and intervention (Ciclovía defined: ≥2 events/month; ≥1 km; streets closed to motor vehicles) and outcomes (public health/PA implications) are described; no explicit comparator. | Overall AMSTAR-2 confidence in review findings: Critically low. Rationale: at least two critical weaknesses—no a-priori protocol (Item 2), no risk-of-bias appraisal (Item 9), and no consideration of RoB in interpretation (Item 13), despite a very broad and thorough search (Item 4). As a result, this article is best treated as a narrative/scoping overview of Ciclovía programs rather than a systematic review producing graded evidence. |
|  |  |  | 2. Protocol established a priori / deviations justified | No | No protocol or preregistration reported. Methods describe what was done but not a pre-specified protocol. |  |
|  |  |  | 3. Rationale for eligible study designs | Yes | Review focuses on programs and includes peer-reviewed and grey literature plus expert input to capture non-indexed program info, but does not explicitly justify specific research designs. |  |
|  |  |  | 4. Comprehensive literature search | Yes | Multiple databases (LILACS, MEDLINE, MEDCARIB, PAHO, WHOLIS, SCIELO), web/Google, Facebook groups, RAFA/PANA outreach, expert consultations, follow-up searches, captures grey literature. |  |
|  |  |  | 5. Study selection in duplicate | Yes | Materials “screened and reviewed independently by 1 investigator and 2 research assistants,” implying more than one reviewer, but duplicate agreement procedures not detailed. |  |
|  |  |  | 6. Data extraction in duplicate | No | Not reported. (No statement about duplicate data extraction.) |  |
|  |  |  | 7. List of excluded studies with justification | Partial Yes | Provides an explicit list of excluded programs and reasons (irregular, inactive, <1 km, etc.); not a PRISMA-style list of excluded studies/citations. |  |
|  |  |  | 8. Description of included studies in adequate detail | Yes | Describes number and distribution of included programs, frequency, duration, length, participation estimates; detailed characteristics table referenced. |  |
|  |  |  | 9. Risk of bias (RoB) assessment for included studies | No | No RoB/quality assessment reported for included materials. (Not described in Methods/Results.) |  |
|  |  |  | 10. Report sources of funding for included studies | No | Funding of included primary sources not reported. |  |
|  |  |  | 11. Appropriate meta-analytic methods (if MA done) |  | No meta-analysis; review is narrative/descriptive. |  |
|  |  |  | 12. RoB accounted for in meta-analysis/synthesis |  | No meta-analysis. |  |
|  |  |  | 13. Consideration of RoB when interpreting results | No | Conclusions do not reference study-level bias/quality because none was assessed. |  |
|  |  |  | 14. Explanation of heterogeneity (if MA) |  | No meta-analysis. |  |
|  |  |  | 15. Investigation of publication bias (if MA) |  | No meta-analysis. |  |
|  |  |  | 16. Report of review authors’ conflicts of interest | Not reported | The PDF opening page lists affiliations; a formal COI statement isn’t shown in the article text we have. |  |
| 16.0 | MMAT | Innovative participatory evaluation methodologies to assess and sustain multilevel impacts of two community-based physical activity programs for women in Colombia | 1.1 Qualitative approach appropriate? | Yes | Participatory action evaluation using Our Voice and Ripple Effects Mapping to capture multilevel impacts and context is well aligned with the aims. | Strong qualitative rigor with rich, participatory data and coherent analysis. Typical limitations (acknowledged in the paper) include small, non-probabilistic samples and potential self-selection, which affect transferability rather than internal credibility. |
|  |  |  | 1.2 Data collection methods adequate? | Yes | Multiple complementary methods (geocoded photos + narratives, group mapping, stakeholder sessions) with clear procedures and participants (program users and stakeholders). |  |
|  |  |  | 1.3 Findings derived from the data? | Yes | Themes (facilitators/barriers, social bonding, empowerment, sustainability) are presented as outputs of the participatory data. |  |
|  |  |  | 1.4 Interpretation substantiated by data? | Yes | Interpretations reference participant-generated evidence (photos/narratives, REM maps) and are tied back to the socio-ecological framing. |  |
|  |  |  | 1.5 Coherence between data sources, collection, analysis, interpretation? | Yes | Good coherence: methods are described, analyses thematically synthesized, and interpretations reflect the integrated participatory outputs. |  |
| 17.0 | MMAT | Unintended impacts of the Open Streets program on noise complaints in  New York City | 3.1 Participants/units representative of the target population? | Yes | Analysis covers all NYC census tracts (N≈2,167) for summer 2019 vs 2021, so the ecological “sample” is effectively complete for the city. | Strong for an ecological, non-randomized design: citywide coverage, appropriate measures, and thoughtful adjustment/robustness checks. Usual caveats apply with residual confounding and complaints as a proxy (annoyance vs. measured noise), but the authors acknowledge these and test sensitivity to key assumptions. |
|  |  |  | 3.2 Measurements appropriate (valid/reliable)? | Yes | Exposure = proportion of Open Streets per tract; outcome = 311 noise complaints (established proxy with prior validation in NYC); aligns with study aims. |  |
|  |  |  | 3.3 Complete outcome data? | Yes | Daily complaints modeled citywide with tract random effects; results table describes distributions for all tracts across poverty quartiles in both periods. |  |
|  |  |  | 3.4 Confounders accounted for? | Yes | Models adjust for calendar time, day of week, COVID period, SES (poverty), density; also test weekday/weekend interactions and multiple sensitivity analyses. |  |
|  |  |  | 3.5 Intervention/exposure administered (occurred) as intended? | Yes | Exposure captured from official datasets; robustness checked using an alternative Open Streets dataset and controls for POIs/Open Restaurants and spatial dependence. |  |
| 18.0 | MMAT | Use of an Elevated Avenue for Leisure-Time Physical Activity by Adults from Downtown São Paulo, Brazil | 3.1 Are participants/units representative of the target population? | Partial / Can’t tell | Cluster sampling of adults living ≤1.5 km from access points; 12,030 numbers drawn, 235 completions. Low response suggests limited representativeness, although post-stratification weights were applied. | Methodologically appropriate for an analytic cross-sectional study: clear exposure/outcome definitions, adjusted regression, and weighting to address low response. Main limitations are representativeness (small completed sample vs large draw) and self-reported exposure, which may introduce bias. Still, the association is precisely estimated (e.g., PR 2.19; 95% CI 1.66–2.90 for meeting LTPA among users vs non-users), consistent with the study aims. |
|  |  |  | 3.2 Are measurements appropriate (valid/reliable)? | Yes | LTPA captured by structured questionnaire with domains (walking, moderate, vigorous) and analyzed as meeting ≥150 min/wk; exposure defined as use of the Minhocão and distance to the nearest access. These align with the research question. |  |
|  |  |  | 3.3 Are there complete outcome data? | Yes | Analytic sample comprised 235 completed questionnaires; results report LTPA outcomes for the full analytic sample. |  |
|  |  |  | 3.4 Have confounders been accounted for in the design/analysis? | Yes | Multivariable Poisson regression with stepwise adjustment: sex, age, education, distance, and environmental barriers; post-stratification weights used to mitigate response bias. |  |
|  |  |  | 3.5 Did the intervention/exposure occur as intended? | Can’t tell | Exposure (“use of the Minhocão”) is self-reported; potential misclassification can’t be ruled out (distance categories are objective, but “use” depends on recall/report). (Methods report exposure definitions but not validation.) |  |
| 19.0 | MMAT | Prevention of childhood obesity and food policies in Latin America: from research to practice | 1.1 Appropriate qualitative approach? | Yes | A case study methodology is stated; cases were coded for components explaining successful implementation/sustainability using the CAS framework—appropriate for a policy translation question. | As a qualitative, framework-driven case synthesis, the study shows good coherence and clear findings. The main limitation is insufficient transparency on data collection for each case (sources/sampling), hence 1.2 = Can’t tell. Still, the CAS-guided coding yields actionable implementation/translation insights. |
|  |  |  | 1.2 Data collection methods adequate? | Can’t tell | Case studies were developed by policy experts on each topic; the paper does not fully specify primary data sources or systematic document sampling beyond expert-developed narratives (no interview/records protocol described). |  |
|  |  |  | 1.3 Findings derived from the data? | Yes | The coding produced key elements (evidence base, civil-society advocacy, political will, intersectoral legislation/negotiation) presented as results. |  |
|  |  |  | 1.4 Interpretation substantiated by data? | Yes | Interpretations link back to the coded case studies and to the CAS constructs guiding analysis (feedback loops, networks, path dependence). |  |
|  |  |  | 1.5 Coherence between sources, collection, analysis, interpretation? | Yes | Clear chain: policy case selection → expert-authored case development → sub-team CAS coding → synthesis of cross-cutting elements. |  |
| 20.0 | MMAT | Evaluation of Event Physical Activity Engagement at an Open Streets Initiative Within a Texas–Mexico Border Town | 3.1 Are participants/units representative of the target population? | Can’t tell / Partial | Intercept surveys at 4 CycloBia 2015 events with a convenience sample of attendees (n interviewed across June–Dec); representativeness of all attendees is uncertain. | Suitable analytic, non-randomized design with appropriate PA measures, city-specific context, and adjusted modeling. The main limitation is uncertain representativeness due to convenience intercept sampling; nonetheless, findings (e.g., high cycling share and predictors of meeting guidelines) are consistent with the study aims and methods. |
|  |  |  | 3.2 Are measurements appropriate (valid/reliable)? | Yes | Outcomes include event PA engagement and meeting PA guidelines, captured via on-site interviews; behaviors along the route/hubs observed with adapted SOPARC—standard tools for PA evaluation. |  |
|  |  |  | 3.3 Are there complete outcome data? | Yes | Results report data for all interviewed attendees across the four events with descriptive tables (e.g., totals per event, % meeting guidelines). |  |
|  |  |  | 3.4 Have confounders been accounted for in the design/analysis? | Yes | Used logistic regression (unadjusted & adjusted) including past event attendance, sex, age, Hispanic ethnicity to estimate odds of meeting PA guidelines. |  |
|  |  |  | 3.5 Did the exposure occur as intended during the study? | Yes | Exposure variables (e.g., past event attendance) were measured as defined and analyzed per protocol; the study also included route counts/observations to characterize event activity as implemented |  |
| 21.0 | MMAT | Move on Bikes Program: A Community-Based Physical Activity Strategy in Mexico City | 3.1 Are participants/units representative of the target population? | Can’t tell / Partial | On-route intercept survey of participants (n=679) at 16 observation points; attendees (not the general population) were sampled during program hours, so representativeness of all users is uncertain. | Appropriate analytic, non-randomized design for an open-streets evaluation: relevant measures, clear modeling of correlates, and strong descriptive context. Main limitations are uncertain representativeness (intercept sampling of attendees) and self-reported exposure for the regression, which can introduce bias. Still, estimates (e.g., high MVPA accrued during events; correlates of frequent attendance) are consistent with the study aims. |
|  |  |  | 3.2 Are measurements appropriate (valid/reliable)? | Yes | PA minutes and intensity estimated using video-derived average speeds by activity; survey captured sociodemographics, travel mode to the route, and attendance frequency—appropriate for evaluating event PA and correlates. |  |
|  |  |  | 3.3 Are there complete outcome data? | Yes | Analyses report outcomes for the full analytic sample (e.g., MVPA minutes, % meeting ≥150 min/week during the event, and counts/attendance estimates). |  |
|  |  |  | 3.4 Have confounders been accounted for in design/analysis? | Yes | Ordinal regression modeled attendance frequency with multiple covariates (e.g., sex, age, activity level, travel mode, accompanying status). |  |
|  |  |  | 3.5 Did the exposure/intervention occur as intended (or was exposure measured accurately)? | Can’t tell | Exposure used in modeling, self-reported attendance frequency, may be prone to recall/report bias; objective program counts support context, but the modeled exposure itself is self-report. |  |
| 22.0 | MMAT | A “Ciclovia” in San Francisco: Characteristics and Physical Activity Behavior of Sunday Streets Participants | 3.1 Is the sampling strategy relevant to the research question? | Yes (with caveats). Systematic on-route intercept (“every fifth participant”; groups included) across the entire route — appropriate for describing event participants that day. |  | Appropriate quantitative design, however, representativeness and nonresponse are unclear/high-risk, so treat effect estimates as potentially biased toward more engaged/active attendees. |
|  |  |  | 3.2 Is the sample representative of the target population? | Can’t tell. Only 3 events were sampled while the season spanned multiple routes/events; nonresponse not quantified, so representativeness of all Sunday Streets participants is uncertain. |  |  |
|  |  |  | 3.3 Are the measurements appropriate? | Yes. Survey items drew on existing instruments, were piloted/translated; PA defined using established wording; subjective vitality scale showed good internal reliability (α=.84). |  |  |
|  |  |  | 3.4 Is the risk of nonresponse bias low? | No / Can’t tell. Survey took ~20–30 minutes; authors note refusals while people were active, but do not report a response rate — suggesting potential nonresponse bias. |  |  |
|  |  |  | 3.5 Is the statistical analysis appropriate? | Yes. Appropriate descriptive statistics and between-group MANOVAs with relevant outcomes (e.g., PA frequency, session duration) and a clear results table. |  |  |
| 23.0 | MMAT | Target Population Involvement in Urban Ciclovias: A Preliminary Evaluation of St. Louis Open Streets | 4.1 Sampling strategy relevant? | Yes | On-route observations at 3 locations and on-site intercepts at natural stopping points—appropriate for describing event participants. | Good fit for a preliminary descriptive evaluation (clear methods, appropriate measures). Main limitations: uncertain representativeness and nonresponse bias, so generalizability beyond surveyed attendees is limited. |
|  |  |  | 4.2 Sample representative of target population? | Can’t tell | Only some 2010 events sampled; survey limited to adults at selected points/times. Representativeness of all attendees is uncertain. |  |
|  |  |  | 4.3 Measurements appropriate? | Yes | Standardized counts by mode/age/sex and structured questionnaire on PA, travel, spending, demographics—fit the aims. |  |
|  |  |  | 4.4 Risk of nonresponse bias low? | No | Survey response rate 65.6%; refusals noted; potential bias remains. |  |
|  |  |  | 4.5 Statistical analysis appropriate? | Yes | Descriptive/frequency statistics reported for observations and surveys, aligned with descriptive aims. |  |
| 24.0 | AMSTAR-2 | Examining the Implementation of Play Streets: A Systematic Review of the Grey Literature | 1. Research question & inclusion criteria include PICO? | Yes | Aim and inclusion criteria are described (RE-AIM focus; Play Streets/Play-Streets–style definitions). | Even though the methods are transparent (PRISMA tracking; duplicate screening/extraction), missing a preregistered protocol, no full excluded-studies list, and—most importantly—no formal risk-of-bias assessment of included sources push the AMSTAR 2 rating to critically low. This doesn’t negate the paper’s descriptive value, but it limits confidence in causal inferences or generalizable effectiveness conclusions. |
|  |  |  | 2. Protocol established a priori / deviations justified | No | No protocol registration is reported → No. (Not mentioned anywhere; PRISMA tracking is noted but not a preregistered protocol.) |  |
|  |  |  | 3. Rationale for eligible study designs | Yes | Rationale for focusing on grey literature due to dearth of academic implementation detail → Yes. |  |
|  |  |  | 4. Comprehensive literature search | Partial Yes | Databases and terms are specified and searches updated, plus reference screening → Partial Yes (limited engines; no registries). |  |
|  |  |  | 5. Study selection in duplicate | Yes | Two researchers independently screened → Yes. |  |
|  |  |  | 6. Data extraction in duplicate | Yes | Two extracted; third/fourth checked consistency → Yes. |  |
|  |  |  | 7. List of excluded studies with justification | No | Exclusion reasons are described, and 7 items were excluded for lack of detail, but no full list is presented → No. |  |
|  |  |  | 8. Description of included studies in adequate detail | Partial Yes | Table of locations/dates/times; little demographic detail → Partial Yes. |  |
|  |  |  | 9. Risk of bias (RoB) assessment for included studies | No | No formal RoB assessment of grey-literature sources → No. |  |
|  |  |  | 10. Report sources of funding for included studies | No | Review funding stated; funding of included reports generally not reported → No. |  |
|  |  |  | 11. Appropriate meta-analytic methods (if MA done) |  |  |  |
|  |  |  | 12. RoB accounted for in meta-analysis/synthesis |  |  |  |
|  |  |  | 13. Consideration of RoB when interpreting results | No | Review funding stated; funding of included reports generally not reported → No. |  |
|  |  |  | 14. Explanation of heterogeneity (if MA) |  |  |  |
|  |  |  | 15. Investigation of publication bias (if MA) |  |  |  |
|  |  |  | 16. Report of review authors’ conflicts of interest | Yes | Declared; none reported → Yes. |  |
| 25.0 | MMAT | Adaptation of the Recreovía During COVID-19 Lockdowns Making Physical Activity Accessible to Older Adults in Bogotá, Colombia | 5.1 Adequate rationale for using mixed methods? | Yes | The paper explicitly describes a convergent mixed-methods design to assess how Recreovía adapted (program level) and to characterize older adults’ PA (individual/context levels). Mixed methods are justified by the dual aim of implementation description and behavior assessment. | Strong mixed-methods execution with clear rationale, good integration of qualitative explanations and quantitative park/user data, and practical implications for delivering PA to older adults under restrictions. Main gap is the absence of an explicit treatment of discordant quant–qual findings; conclusions are appropriately tempered by the cross-sectional nature and recruitment constraints. |
|  |  |  | 5.2 Effective integration of components? | Yes | Quantitative components (SOPARC park observations; PARA park quality; PA classification; plus survey/accelerometry) are interpreted alongside interviews with the program manager, instructors, and users to explain how and why adaptations worked across three delivery strategies (Facebook Live, balcony sessions, outdoor classes). Findings about higher park use and vigorous PA on Recreovía days are discussed with qualitative reports of positive experiences. |  |
|  |  |  | 5.3 Outputs of integration adequately interpreted? | Yes | The discussion links integrated results to implications (e.g., 72–79% meeting WHO PA guidelines during adapted delivery; more women and more vigorous activity on program days; relevance for age-friendly cities and emergencies). |  |
|  |  |  | 5.4 Divergences or inconsistencies addressed? | Partial / Can’t tell | Authors acknowledge limitations (cross-sectional design, inability to recruit balcony-session participants, no long-term follow-up), but they do not explicitly analyze quant–qual divergences. |  |
|  |  |  | 5.5 Components meet quality criteria of each tradition? | Yes | Quant: SOPARC procedures (multiple observation periods, trained observers, PA intensity categories) and PARA audits are clearly described. Qual: Interviews with program leadership/instructors/users detail the three adaptation strategies and implementation context. Reporting indicates coherent data collection/analysis for both strands. |  |
| 26.0 | MMAT | CicLAvia: Evaluation of participation, physical activity and cost of an open streets event in Los Angeles | 4.1 Sampling strategy relevant? | Yes | Route-wide camera placement and hub intercepts are well-aligned with describing event-day attendance and participant behavior. | Methodologically appropriate for descriptive, event-day estimation. The study triangulates route-wide cameras, on-route intercept surveys, and GPS-based speed/distance to estimate participation, PA dose (MET-hours), and cost per MET-hour, which fits its aims and meets MMAT criteria for relevant sampling strategy, appropriate measures, and suitable descriptive analysis. However, representativeness is uncertain (survey convenience sample at hubs; response rate not clearly reported), so nonresponse/selection bias may limit generalizability beyond surveyed attendees. |
|  |  |  | 4.2 Sample representative of target population? | Can’t tell | Camera counts cover the route, but survey respondents are a convenience/on-route sample at hubs; representativeness of all attendees cannot be verified. |  |
|  |  |  | 4.3 Measurements appropriate? | Yes | Cameras for volume, surveys for duration/mode, GPS-based speeds to estimate distance/intensity, then MET-hour calculation and cost per MET-hour—all matched to aims. |  |
|  |  |  | 4.4 Risk of nonresponse bias low? | Can’t tell | Response rates for intercept surveys are not clearly reported; potential for nonresponse remains. (Counts are not subject to survey nonresponse.) |  |
|  |  |  | 4.5 Statistical analysis appropriate? | Yes | Descriptive estimation and triangulation (counts + surveys + GPS) appropriately produce attendance, MET-hours, and cost metrics (e.g., 36,800–54,740 participants; 176,500–263,000 MET-hours; $1.29–$1.92 per MET-hour). |  |
| 27.0 | MMAT | Dover Micro Open Street Events: Evaluation Results and Implications for Community-Based Physical Activity Programming | 4.1 Sampling strategy relevant? | Yes | On-site every-third adult intercept at entry/exit points; standardized staff training before first event. Appropriate for describing event attendees. | Good descriptive fit for a pilot, multi-level Open Streets evaluation: relevant sampling approach; appropriate, triangulated measures across individual, program, and neighborhood levels; and suitable descriptive analysis. Main limitations are uncertain representativeness of surveyed attendees and nonresponse bias, plus cross-sectional self-report for several outcomes, generalizability beyond those present at the sampled events is limited. Findings should be interpreted as descriptive signals of reach and short-term intentions rather than causal effects. |
|  |  |  | 4.2 Sample representative of target population? | Can’t tell | Survey limited to eligible English-speaking adults encountered at selected points/times; only a subset of all attendees completed surveys (n=78). Representativeness of all MOSE participants is uncertain. |  |
|  |  |  | 4.3 Measurements appropriate? | Yes | 33-item survey (PA behaviors/intentions, facility awareness, community connectedness, health status), program EMA (Ecological Movement Assessments) counts, and neighborhood audit align with aims. |  |
|  |  |  | 4.4 Risk of nonresponse bias low? | No | Reported survey response 64.9%; authors note inability to compare responders vs non-responders and acknowledge possible response bias. |  |
|  |  |  | 4.5 Statistical analysis appropriate? | Yes | Descriptive analyses (means/SD, percentages) using SPSS match the study’s descriptive purpose. |  |
| 28.0 | MMAT | Quality of Life, Physical Activity, and Built Environment Characteristics Among Colombian Adults | 3.1 Participants/units representative? | Yes | Citywide adult sample (n≈1,334), response rate 66.7%; neighborhood SES in sample comparable to Bogotá’s, supports representativeness, though nonresponse may remain. | Strong analytic, non-randomized design for a population sample with validated measures and appropriate multilevel adjustment. Main limitation is self-report (PA/participation) and potential nonresponse bias despite a reasonable 66.7% response. |
|  |  |  | 3.2 Measurements appropriate (valid/reliable)? | Yes | HR-QOL via WHOQOL-BREF (cognitive interviewing; α≈0.83); perceived health via CDC Healthy Days; “future” item from WHOQOL; PA via IPAQ-long (adapted); BE via GIS for density/diversity/design/distance/transport (incl. Ciclovía length). |  |
|  |  |  | 3.3 Complete outcome data? | Yes | Outcomes reported for the analytic sample with distributions; PA/Ciclovía participation proportions provided. |  |
|  |  |  | 3.4 Confounders accounted for? | Yes | Multilevel models adjusted for individual covariates (sex, age, marital status, education, occupation, SES) and BE factors; ICCs and variance explained at block level reported. |  |
|  |  |  | 3.5 Exposure occurred/measured as intended? | Yes | PA and Ciclovía participation self-reported; BE exposures derived from administrative GIS (objective). Acceptable for cross-sectional analytic aims. |  |
| 29.0 | MMAT | Network Analysis of Bogota’s Ciclovia Recreativa, a Self- Organized Multisectorial Community Program to Promote Physical Activity in a Middle-Income Country | 3.1 Participants/units representative? | Yes (with caveat) | Target “population” = organizations involved in Ciclovía. Frame built from history review + program director listing; 22/25 responded (88%). Some nonresponse remains but coverage is high. | Methodologically strong analytic SNA: near-complete organizational roster, appropriate measures, and ERGM to handle network dependencies and covariates. Results (e.g., Sports & Recreation/Government/Security centrality; importance of the organization ↗ integration, years working on ciclovia ↘ integration; positive transitivity) are supported by the modeling. Main limitations are cross-sectional design and partial nonresponse (3 orgs), so causal claims about collaboration dynamics over time aren’t warranted. |
|  |  |  | 3.2 Measurements appropriate (valid/reliable)? | Yes | Relational link attributes: integration/contact/importance; node attributes: leadership/years/sector—well matched to aims. |  |
|  |  |  | 3.3 Complete outcome data? | Yes | Analyses use the full responding network (22 orgs), report centrality, communities, and model outputs. |  |
|  |  |  | 3.4 Confounders accounted for? | Yes | Uses exponential random graph models with structural terms (GWESP, GWOdegree, GWDSP) plus covariates (importance, years) to model likelihood of integration—appropriate adjustment for network structure. |  |
|  |  |  | 3.5 Exposure/intervention occurred/measured as intended? | Yes | Data collected via standardized questionnaire (face-to-face/email/phone) in Mar–Jun 2009; constructs align to collaboration as intended. Minor recall/reporting risks acknowledged by design. |  |
| 30.0 | MMAT | Moving the Barricades to Physical Activity: A Qualitative Analysis of Open Streets Initiatives Across the United States | 1.1 Is the qualitative approach appropriate to the research question? | Yes | Phenomenological/experiential focus via organizer interviews matches the aim to capture perceptions and lived implementation experience. | Strong qualitative fit for capturing organizers’ perspectives on Open Streets: appropriate method, adequate data collection, and coherent analysis→interpretation. Primary limitation is limited reporting of rigor checks (e.g., member checking/dual coding), so treat results as well-founded thematic insights rather than exhaustive triangulated evidence. |
|  |  |  | 1.2 Are the data collection methods adequate to address the research question? | Yes | Structured telephone interviews with lead organizers (27 of the 2011 events’ contacts), recorded and transcribed; sampling is fit-for-purpose for expert informants. |  |
|  |  |  | 1.3 Are the findings adequately derived from the data? | Yes | Themes (e.g., health/transport goals, funding/logistics challenges, democratizing public space) are grounded in interview data and presented with supporting explanation. |  |
|  |  |  | 1.4 Is the interpretation of results sufficiently substantiated by data? | Yes | Interpretations align with reported themes; typical qualitative credibility procedures (e.g., member checking, inter-coder agreement). |  |
|  |  |  | 1.5 Is there coherence between data sources, collection, analysis, and interpretation? | Yes | Clear line from organizer interviews → transcription → constant comparative coding → thematic conclusions about implementation, barriers, and perceived benefits. |  |
| 31.0 | MMAT | Moving targets: Promoting physical activity in public spaces via open streets in the US | 1.1 Appropriate qualitative approach? | Yes | Semi-structured interviews with program organizers and constant comparative analysis are well-matched to exploring implementation barriers/facilitators. | Strong qualitative fit for capturing organizers’ lived implementation experience nationwide. Adequate sampling and analytic approach yield actionable themes (funding, permitting, branding/communication) that align with the paper’s purpose. |
|  |  |  | 1.2 Data collection adequate? | Yes | 32 unique programs interviewed; interviews were transcribed verbatim for analysis—adequate depth for thematic development. |  |
|  |  |  | 1.3 Findings derived from the data? | Yes | Themes (e.g., funding costs ≥$10,000/event, permitting hurdles, branding/communication challenges) are clearly grounded in interview data, with illustrative quotes. |  |
|  |  |  | 1.4 Interpretation substantiated by data? | Yes | Interpretations about expansion barriers and program sustainability are supported by quotes; formal credibility checks (e.g., member checking) aren’t detailed. |  |
|  |  |  | 1.5 Coherence between data sources, collection, analysis, interpretation? | Yes | Clear chain: identify programs → conduct/record interviews → transcribe → constant comparative coding → report themes (barriers, facilitators, success metrics). |  |
| 32.0 | MMAT | Translating evidence to policy: urban interventions and physical activity promotion in Bogotá, Colombia and Curitiba, Brazil | 1.1 Qualitative approach appropriate? | Yes | A policy case-study design guided by the Physical Activity Policy Framework directly matches the question about policy processes shaping the two programs. | High-quality qualitative policy analysis. The design is well-aligned with the research question, data collection is robust (two cities, multiple sectors, documented sampling), and analysis is rigorous via framework analysis with steps to enhance credibility (dual coding in one site, negative-case searches). |
|  |  |  | 1.2 Data collection adequate? | Yes | Multi-source strategy: systematic literature/grey-literature review plus semi-structured interviews with diverse actors (Bogotá n=20; Curitiba n=19), recruited via expert listing and snowball sampling; interviews were recorded, transcribed, IRB-approved. |  |
|  |  |  | 1.3 Findings adequately derived from data? | Yes | Results synthesize multisector policies enabling each program and present a structured account (history, sectoral policies) and a comparative Table 1 of program characteristics/policy enablers. |  |
|  |  |  | 1.4 Interpretation substantiated by data? | Yes | Analysis used framework analysis with clear stages (familiarization → thematic framework → indexing → charting → mapping), dual-coding for Bogotá, attention to contrasts/negative cases, and audit trails (memos, charts). |  |
|  |  |  | 1.5 Coherence between data sources, collection, analysis, interpretation? | Yes | Consistent chain: policy framework → literature review + stakeholder interviews → framework analysis → policy themes and sector-specific conclusions (e.g., sports/recreation & urban planning as key enablers). |  |
| 33.0 | MMAT | Assessing the effect of physical activity classes in public spaces on leisure_x0002_time physical activity: “Al Ritmo de las Comunidades” A natural experiment in Bogota, Colombia | 3.1 Are participants/units representative of the target population? | Yes | Adults sampled systematically from parks/households/community groups; eligibility specified; large citywide recruitment. Potential selection bias acknowledged. | Robust natural-experiment design with appropriate self-report and objective PA measures and multilevel modeling. Main threats are incomplete follow-up, modest accelerometer sample, and potential selection bias—all acknowledged by authors. |
|  |  |  | 3.2 Are measurements appropriate (valid/reliable)? | Yes | LTPA via IPAQ-LT with guideline-based thresholds; objective PA via ActiGraph GT3X with standard wear/processing; BMI measured objectively. |  |
|  |  |  | 3.3 Are there complete outcome data? | No | Follow-up only in new/control parks; loss to follow-up ≈27% and small accel. subsample—authors note under-power and reliance on self-report. |  |
|  |  |  | 3.4 Have confounders been accounted for in the design/analysis? | Yes | General linear mixed models with park as random effect; fixed effects included gender, age, occupation, education, marital status, SES, distance to park, and group. |  |
|  |  |  | 3.5 Did the intervention/exposure occur as intended? | Yes | New sites offered 45-min classes every Sunday 8:00–12:00; exposure was naturalistic and voluntary to preserve external validity. |  |
| 34.0 | MMAT | Implementation of childhood obesity prevention and control policies in the United States and Latin America: Lessons for cross-border research and practice | 1.1 Qualitative approach appropriate? | Yes | A comparative case-study synthesis with an implementation framework (RE-AIM) fits the policy-translation questions. | Strong qualitative synthesis using an established implementation framework with multi-source evidence (docs + key informants). Conclusions (need for evidence-based advocacy, political will, scalable designs, and robust equity-focused monitoring/evaluation) are well grounded in the mapped cases. |
|  |  |  | 1.2 Data collection adequate? | Yes | Uses literature/grey sources plus key-informant interviews across US/LATAM cases, adequate breadth for thematic synthesis. |  |
|  |  |  | 1.3 Findings derived from the data? | Yes | Cross-cutting themes (e.g., evidence-based advocacy, political will, context-specific adaptation) are presented as results of the RE-AIM mapping. |  |
|  |  |  | 1.4 Interpretation substantiated by data? | Yes | Interpretations explicitly reference the RE-AIM domains and case evidence when drawing lessons for practice. |  |
|  |  |  | 1.5 Coherence between sources, collection, analysis, interpretation? | Yes | Coherent chain: case identification → documents/interviews → RE-AIM coding → synthesized lessons on reach, implementation, maintenance, equity monitoring. |  |
| 35.0 | MMAT | Mixed method assessment of built environment and policy  responses to the COVID-19 pandemic by United States  municipalities focusing on walking and bicycling actions | 5.1 Rationale for using mixed methods | Yes | Authors aimed to systematically describe actions across cities and add context/explanation via interviews—explicit mixed-methods purpose. | Good mixed-methods study with quantitative coverage with verification, plus qualitative interviews that explain how and why responses varied. Primary limitations are typical of rapid scans, possible documentation bias (what cities posted), limited detail on inter-rater metrics, and no explicit treatment of quant–qual divergences. Results should be interpreted as descriptive evidence of municipal actions during 2020 (e.g., closures, shared/slow/open streets, dining reallocation) rather than causal effects on walking/bicycling, something the authors also note as a next step. |
|  |  |  | 5.2 Integration of qualitative & quantitative components | Yes | Quantitative scan (web audit + cross-check with Shifting Streets DB) produced the action counts; interviews contextualized and added insight to these results. |  |
|  |  |  | 5.3 Interpretation of the integrated outputs | Yes | Findings combine counts—353 actions; 294 likely facilitated vs 59 limited—with interview interpretation; authors call for next-step causal evaluations. |  |
|  |  |  | 5.4 Handling divergences/inconsistencies | Partial / Can’t tell | Methods describe triangulation (municipal websites + crowd-sourced DB) and verification (12% second rater), but the paper doesn’t explicitly analyze discordant quant–qual findings. |  |
|  |  |  | 5.5 Adherence to quality criteria for each component | Yes | Quant: comprehensive frame (314 cities), dual-source verification, and inter-rater reliability check on a 12% subsample. Qual: recorded, transcribed, and coded interviews with municipal leaders. |  |
| 36.0 | MMAT | Do Health Benefits Outweigh the Costs of Mass Recreational Programs? An Economic Analysis of Four Ciclovía Programs | 3.1 Are participants/units representative of the target population? | Can’t tell | Units are the four entire programs (not a sampled subset), so results are representative for those programs. Generalizability to other cities is uncertain. | Methodologically appropriate economic appraisal with transparent formulas and sensitivity analyses (varying DHB, user counts, and cost types; Monte Carlo for Bogotá). Findings, cost-benefit ratios >1 for Bogotá, Medellín, and San Francisco; ≈1–1.23 for Guadalajara, are credible for the four programs studied, but rest on modeled DHB assumptions and limited clinical-cost data in some cities. Treat as good-quality economic evidence, not causal health-effect estimation. |
|  |  |  | 3.2 Are measurements appropriate (valid/reliable)? | Yes | Clear specification of average CBA, explicit DHB equations/assumptions (inflation-adjusted US DHB; city-specific scaling using medical-cost ratios), and program cost components; HEAT also referenced. |  |
|  |  |  | 3.3 Are there complete outcome data? | Yes | Cost benefit ratios reported for all four programs; where inputs were missing (e.g., Guadalajara medical costs), the authors used bounded ranges. |  |
|  |  |  | 3.4 Have confounders been accounted for in design/analysis? | No | This is an economic evaluation (not an effect study); no adjusted effect modeling was undertaken—appropriate to the aim but this MMAT item is not met. |  |
|  |  |  | 3.5 Did the intervention/exposure occur as intended (or measured accurately)? | Partial | Program exposure and costs were taken from directors/surveys; however, for San Francisco the DHB assumes a weekly program (while it actually ran monthly), flagged by the authors as a modeling assumption. |  |
| 37.0 | MMAT | Ciclovía Participation and Impacts in San Diego, CA: The First CicloSDias | 4.1 Sampling strategy relevant? | Yes | City-wide RDD (random digit dialing) sampling before/after the event; on-route intercept surveys at 4 hubs; counts at 3 locations along a 5.2-mile route—appropriate strategies to describe awareness, attendees, and participation, though intercepts are convenience-based. | Good descriptive evaluation with multiple complementary measures (RDD awareness, intercept PA/demographics, business survey, on-route counts). Major limitation is uncertain representativeness/nonresponse; hence generalizability is limited. Findings, ~8.3k attendees; mean 144 min PA with high guideline attainment; mostly neutral/positive business reports; under-representation of Latino/non-White residents, should be interpreted as event-day descriptive evidence, not causal effects. |
|  |  |  | 4.2 Sample representative of target population? | Can’t tell | The paper doesn’t document response rates for the intercept or RDD surveys; authors also report Latinos/non-White residents were under-represented among attendees when compared to city demographics. |  |
|  |  |  | 4.3 Measurements appropriate (valid/reliable)? | Yes | Attendance estimation via standardized counting procedure; PA minutes self-reported in intercept survey; domains cover travel, marketing reach, PA, purchases, and demographics; business impacts captured with a 5-point comparative scale. |  |
|  |  |  | 4.4 Risk of nonresponse bias low? | Can’t tell | Survey response rates not reported; city-wide RDD oversampled near-route ZIP codes (50%) for awareness analyses, which is reasonable but doesn’t address nonresponse. |  |
|  |  |  | 4.5 Statistical analysis appropriate? | Yes | Analyses are descriptive/estimation and match aims (e.g., 8,311 attendance from count method; average 144 min PA; guideline attainment; business outcomes). |  |
| 38.0 | MMAT | Bridging the gap between research and practice: an assessment of external validity of community-based physical activity programs in Bogotá, Colombia, and Recife, Brazil | 1.1 Qualitative approach appropriate? | Yes | A case-study/qualitative design guided by RE-AIM fits the aim of examining external validity dimensions (reach, adoption, implementation, maintenance). | Strong qualitative rigor for policy-and-practice relevance: appropriate framework (RE-AIM), suitable informants, and coherent analysis. Limitations are typical of qualitative case studies (context-specificity, potential social-desirability in KI interviews). Findings credibly support that both programs reach underserved populations, leverage public spaces, and depend on stable funding/policy for maintenance. |
|  |  |  | 1.2 Data collection methods adequate? | Yes | Key-informant interviews across roles (program coordinators, unit leads, instructors) in both cities; purposive sampling is appropriate for program knowledge holders. |  |
|  |  |  | 1.3 Findings derived from the data? | Yes | Themes and exemplars (e.g., reach to underserved groups, free classes in public spaces, organizational capacity, funding/policy for maintenance) are grounded in interview content and RE-AIM coding. |  |
|  |  |  | 1.4 Interpretation substantiated by data? | Yes | Interpretations link directly to coded RE-AIM domains; authors also report member/expert validation, strengthening credibility. |  |
|  |  |  | 1.5 Coherence between sources, collection, analysis, interpretation? | Yes | Consistent chain: purposive (KI) key-informant interviews → recorded/transcribed interviews → constant comparative analysis within RE-AIM → coherent cross-case conclusions for Bogotá/Recife. |  |
| 39.0 | MMAT | Talking the Walk: Perceptions of Neighborhood Characteristics from Users of Open Streets Programs in Latin America and the USA | 1.1 Qualitative approach appropriate? | Yes | A walk-along photo/voice approach with thematic synthesis is well-suited to eliciting lived neighborhood perceptions during Open streets (OS) vs. non-Open Street days. | Strong qualitative design for a feasibility/field study: appropriate citizen-science method, coherent analysis, and cross-source corroboration. Main limitations are small site samples and measurement timing/translation caveats that constrain transferability, both acknowledged by the authors. |
|  |  |  | 1.2 Data collection methods adequate? | Yes | Standardized two-walk protocol (pre non-OS; post OS), DT training, on-route photo/audio justifications, and post-walk Reflection Survey provide rich, multi-source qualitative evidence. |  |
|  |  |  | 1.3 Findings derived from the data? | Yes | Authors report 18 themes (10 shared across ≥2 sites; 4 shared at all sites: community/social connectedness, family-friendly environment, physical activity, safety), explicitly grounded in participants’ DT photo/narratives. |  |
|  |  |  | 1.4 Interpretation substantiated by data? | Yes | Thematic results align with Reflection Survey shifts (e.g., ease of walking, safety, friendliness “seemed better”), supporting inferences about social/health co-benefits of OS. |  |
|  |  |  | 1.5 Coherence between sources, collection, analysis, interpretation? | Yes | Clear chain: DT (Discovery tool) walk-alongs → coded themes → triangulation with Reflection Survey → cross-site conclusions; limitations noted (small samples; translation issues; post defined as OS walk rather than true after-event posttest). |  |
| 40.0 | MMAT | Active Living Logan Square Joining Together to Create Opportunities for Physical Activity | 1.1 Qualitative approach appropriate to the question? | Yes | A community case study framed by the ALbD 5P model (preparation, promotion, programs, policy, physical projects) suits the implementation/process focus. | A sound qualitative case study of a community partnership applying the ALbD framework. Strengths include a well-articulated implementation model, concrete outputs (Open Streets pilots, school and public-space changes), and explicit practical lessons. Limitations are typical of case reports (no formal qualitative coding/triangulation is described; the resident survey is descriptive and not the focus of qualitative analysis), so generalizability is limited; nonetheless, the narrative is well supported by documented activities and context. |
|  |  |  | 1.2 Data collection methods adequate? | Yes | The partnership documented activities and gathered context via a face-to-face resident survey (n≈400) to understand barriers and guide actions. |  |
|  |  |  | 1.3 Findings adequately derived from the data? | Yes | Reported results—piloting Open Streets, advocating the Bloomingdale Trail, and school culture/policy changes—are presented as outputs of the documented partnership process. |  |
|  |  |  | 1.4 Interpretation substantiated by data? | Yes | The paper’s lessons learned (need for a full-time coordinator, culturally relevant strategies, and flexibility) are explicitly tied to the partnership’s experience and outcomes. |  |
|  |  |  | 1.5 Coherence between sources, collection, analysis, interpretation? | Yes | Clear chain: community/partner prep → 5P-guided implementation in schools & community → documented outputs and reflections. Funding and staffing structure are reported. |  |
| 41.0 | MMAT | Air quality impacts of a CicLAvia event in Downtown Los Angeles, CA | 3.1 Participants/units representative? | Partial / Can’t tell | “Units” are route segments/time blocks during one CicLAvia and two comparison Sundays. Good coverage of the event corridor, but representativeness for other routes, seasons, or events is uncertain. | Strong quasi-experimental field study for a single event: appropriate instruments, concurrent control comparisons, and meteorological adjustment. Main limitations are scope (one event, three Sundays), potential instrument/route-specific biases, and limited generalizability beyond the studied corridor and season. Findings (≈−21% UFP and −49% on-road PM₂.₅; neighborhood PM₂.₅ ≈ −12% on average during the event) are credible as short-term, local effects of the temporary street closure. |
|  |  |  | 3.2 Measurements appropriate (valid/reliable)? | Yes | Portable instruments for UFP (CPC/WCPC) and PM₂.₅ (DustTrak); concurrent traffic counts; standard field procedures for mobile monitoring. |  |
|  |  |  | 3.3 Outcome data complete? | Yes | Reports pre/event/post data across three daily sessions for on-road and neighborhood locations; no critical missing blocks noted. |  |
|  |  |  | 3.4 Confounders accounted for? | Yes | Adjusted for meteorology using ratios to a control route; considered time-of-day; documented freeway volumes to rule out broader traffic shifts. |  |
|  |  |  | 3.5 Exposure occurred/measured as intended? | Yes | The event created near-zero traffic on the corridor; measurements captured the planned closure period and immediate surroundings. |  |
| 42.0 | MMAT | The Impact of a Temporary Recurrent Street Closure on Physical Activity in New York City | 4.1 Sampling strategy relevant? | Yes | Screen-line counts at multiple points along the 6.9-mile route to estimate total participation; on-route intercept survey to describe attendee PA and characteristics—appropriate strategies for an event-day descriptive study. | Good descriptive evaluation of NYC’s Summer Streets: appropriate, multi-source event-day measurements and clear reporting of participation and PA dose. Main limitations are representativeness (single day, on-route intercepts) and unreported nonresponse, so generalizability beyond the sampled day/locations is uncertain. Treat findings as event-day descriptive evidence, not causal effects. |
|  |  |  | 4.2 Sample representative of target population? | Can’t tell | One Saturday of a 3-Saturday program; survey covers attendees present where interviewers were stationed. Authors report attendees skewed white, ages 25–64, and Manhattan-based, suggesting possible selection limits vs NYC population. |  |
|  |  |  | 4.3 Measurements appropriate? | Yes | Standardized screen-line counts for volume; 28-item intercept survey for route distance/time and PA; traffic study for vehicular impacts, well-matched to aims. |  |
|  |  |  | 4.4 Risk of nonresponse bias low? | Can’t tell | Intercept survey response rate not clearly reported; refusal while moving is plausible, so nonresponse bias cannot be ruled out. |  |
|  |  |  | 4.5 Statistical analysis appropriate? | Yes | Descriptive estimation (e.g., ~50,000 attendees; ~72–86 min of moderate-equivalent PA per participant; no measurable traffic congestion) aligns with the study’s descriptive purpose. |  |
| 43.0 | AACODS | Taking Physical Activity to the Streets: The Popularity of Ciclovía and Open Streets Initiatives in the United States | Authority | Yes | Clearly identified authors (Hipp, Eyler, Zieff, Samuelson) and labeled as a Commentary in the American Journal of Health Promotion, a peer-reviewed journal; the piece is also cited by subsequent peer-reviewed work, indicating recognition. • (AACODS guidance on Authority). | Suitable for background/context, not for causal inference. High Authority and Significance, accurate within its concise length; coverage is necessarily limited and the tone is pro-Open Streets (acknowledge potential bias). Use it to frame the U.S. landscape (2008–2013) and motivations, then rely on empirical studies for effects. |
|  |  |  | Accuracy | Yes | States its purpose (why Open Streets, growth, motivations) and supports claims with documented references (e.g., U.S. cities hosting events, benefits, prior evaluations). It is an opinion/overview but cites authoritative sources and prior studies. • (AACODS Accuracy prompts). |  |
|  |  |  | Coverage | Partial | Scope is intentionally brief (2-page commentary) summarizing motivations and growth (2008–2013) with a table; limits (period, geographies) are implicit rather than fully explicit. Useful snapshot but not systematic. • (AACODS Coverage prompts). |  |
|  |  |  | Objectivity | Partial / Yes | Stance is supportive of Open Streets (advocacy-oriented tone), but arguments are referenced and not purely opinion. Balance is acceptable for a commentary, though potential positive bias is likely. • (AACODS Objectivity prompts). |  |
|  |  |  | Date | Yes | Publication window and content timeframe are clear (2008–2013 growth; published 2014), so it’s historically appropriate but not reflective of later expansion. • (AACODS Date prompts). |  |
|  |  |  | Significance | Yes | Concise, widely cited snapshot that contextualizes why U.S. cities pursue Open Streets and summarizes motivations/outcomes; often used as an entry-point reference (e.g., cited in Preventive Medicine). (AACODS Significance prompts). |  |
| 44.0 | MMAT | Ciclovía initiatives: engaging communities, partners and policymakers along the route to success | 1.1 Appropriate qualitative approach? | Yes | A comparative case-study/process evaluation matches the goal of explaining development/implementation structures and surfacing practical lessons. | A useful, practice-focused qualitative case comparison with strong ecological validity for implementers. Strengths: multi-source evidence and clear, actionable guidance (collaborations/MOUs, volunteer systems, early route planning, sustained promotion/wayfinding, leveraging longer routes/hours). Limitations: reliance on document review and organizer communications with limited reporting of formal qualitative rigor checks, so findings should be interpreted as well-grounded practice insights. |
|  |  |  | 1.2 Adequate data collection? | Yes | Broad document review + organizer communications provide rich detail across domains (buy-in/collaboration, route selection, programming, partnerships, outreach/media, merchant support, staffing/volunteers, funding). |  |
|  |  |  | 1.3 Findings adequately derived from the data? | Yes | The paper presents convergent findings (many structures/processes similar) and contrasts (staffing/volunteer capacity; funding). It also enumerates actionable lessons (e.g., formalize collaborations; cultivate community/merchant/government buy-in; build volunteer bank; streamline programming; early route selection; longer routes/hours expand reach; fund promotion/wayfinding). |  |
|  |  |  | 1.4 Interpretation substantiated by data? | Yes | Interpretations are explicitly grounded in the compiled evidence and synthesized into recommendations for stakeholders/policymakers; comparative tables further substantiate differences (e.g., staffing/volunteers, sponsors, participant volumes, route-selection goals). |  |
|  |  |  | 1.5 Coherence between sources, collection, analysis, interpretation? | Yes | There’s a coherent chain: documents + organizer input → thematic comparison across domains → lessons learned. However, formal qualitative procedures (e.g., coding schema, inter-coder checks, member-checking) aren’t described in detail. |  |
| 45.0 | MMAT | Prevalence and Factors Associated with Walking and Bicycling for Transport Among Young Adults in Two Low-Income Localities of Bogotá, Colombia | 3.1 Participants/units representative of the target population? | Yes | Multistage, stratified household cluster sample of 18–29 y residents in two low-income localities with a ~98% response rate, appropriate for that defined population. | A strong cross-sectional analytic study for its scope: population-based sampling within two low-income areas, validated PA instrument, and appropriate multivariable modeling. Main limitations are self-report (IPAQ recall), cross-sectional design (no causality), and generalizability restricted to young adults in two Bogotá localities (terrain/context differences noted). |
|  |  |  | 3.2 Measurements appropriate (valid/reliable)? | Yes | Transport PA from Spanish, culturally adapted IPAQ-long; sociodemographic/social/environmental correlates (incl. Ciclovía use) match aims; acknowledged self-report limits. |  |
|  |  |  | 3.3 Complete outcome data? | Yes | High participation and clear reporting of outcome prevalence (walking, bicycling) and correlates for the full analytic sample. |  |
|  |  |  | 3.4 Confounders accounted for in design/analysis? | Yes | Logistic regression models include multiple covariates (sex, education, locality/terrain, leisure-time PA, Ciclovía participation, etc.). |  |
|  |  |  | 3.5 Exposure/intervention occurred or was measured as intended? | Yes | Exposures (e.g., Ciclovía use, leisure PA, social/environmental factors) measured per protocol; all cross-sectional and largely self-reported. |  |
| 46.0 | MMAT | Results from Chile’s 2018 Report Card on Physical Activity for Children and Youth | 4.1 Sampling strategy relevant to the question? | Yes | The scorecard intentionally draws on national data sources (probability-based surveys and official datasets) appropriate for describing country-level indicators. | The 2018 Chile Report Card provides a methodologically appropriate national snapshot using recognized AHKGA methods and broad national data sources. Its main limitations are indicator-level heterogeneity (many rely on adolescent self-report and have gaps for younger ages) and the inability to quantify a unified nonresponse risk. |
|  |  |  | 4.2 Is the sample representative of the target population? | Can’t tell / Partial | Many indicators rely primarily on self-report among adolescents, with limited data for younger children; representativeness therefore varies by indicator and age group. |  |
|  |  |  | 4.3 Are the measurements appropriate? | Yes | Indicators and grading follow the AHKGA (Active Healthy Kids Global Alliance) standardized rubric; however, several grades are based on self-reported PA/sedentary time, which introduces recall/social-desirability bias. |  |
|  |  |  | 4.4 Is the risk of nonresponse bias low? | Can’t tell | As a synthesis, the report card aggregates across surveys with varying response rates; a unified nonresponse assessment is not presented. |  |
|  |  |  | 4.5 Is the statistical analysis appropriate? | Yes | The approach is descriptive grading (A–F) per predefined cut-points rather than inferential modeling—appropriate for a national scorecard. |  |
| 47.0 | MMAT | Policy and Built Environment Changes in Bogota´ and their Importance in Health Promotion | 1.1 Qualitative approach appropriate? | Yes | A city case analysis is suitable to explore how multisector urban/transport policies may influence PA. | A useful, early case account of Bogotá’s urban/transport reforms for PA promotion. Strengths: clear policy descriptions and balanced interpretation (signals benefits while flagging evidence gaps). Limitations: no primary qualitative data or systematic methods; therefore, treat as contextual/background evidence rather than empirical effect evaluation. |
|  |  |  | 1.2 Data collection methods adequate? | Partial | Uses documented municipal policies, program facts, and published reports; however, formal qualitative procedures (e.g., interview guides, coding) are not described. |  |
|  |  |  | 1.3 Findings derived from the data? | Yes | Narrative links policies (e.g., street closures, license-plate restrictions) to plausible traffic/air quality and PA pathways; cites reported traffic/pollution reductions for Pico y Placa. |  |
|  |  |  | 1.4 Interpretation substantiated by data? | Yes | Conclusions emphasize potential PA benefits and explicitly note limited direct evidence and need for research, appropriate caution for a descriptive paper. |  |
|  |  |  | 1.5 Coherence between sources, collection, analysis, interpretation? | Yes | Coherent chain from policy descriptions → expected mechanisms → cautious conclusions; acknowledges cross-sector origins and evaluation gaps. |  |
| 48.0 | MMAT | Participation and Physical Activity in Recreovia of Bucaramanga, Colombia | 4.1 Sampling strategy relevant? | Yes | Repeated, scheduled observations (Sep–Nov 2017; Sundays 8:00–12:00) at predefined points along the 2.5-km route, appropriate for describing event users. | A well-executed descriptive observation study using validated SOPARC methods with excellent reliability, yielding detailed profiles (e.g., street users mostly men/adults with ~98% MVPA; classes mostly women/adults with ~76% vigorous). Main limitations are site/time coverage (four points, event hours only) and corridor-level representativeness. Interpret findings as robust event-hour descriptive evidence, not population-wide estimates. |
|  |  |  | 4.2 Sample representative of target population? | Can’t tell | Coverage limited to four sites and event hours; results may not generalize to the entire corridor or to non-observed Sundays. |  |
|  |  |  | 4.3 Measurements appropriate? | Yes | Standard SOPARC/iSOPARC protocols with reported high reliability (ICC > .95); observers coded sex, age group, activity type, and PA intensity. |  |
|  |  |  | 4.4 Risk of nonresponse bias low? | Not applicable (observational) | No participant survey; counts are unobtrusive observations. Remaining risks are double-counting or misclassification, which the protocol aims to minimize. |  |
|  |  |  | 4.5 Statistical analysis appropriate? | Yes | Descriptive summaries of 38,577 observations and χ² tests to compare distributions across sites/times/strata suit the descriptive aims. |  |
| 49.0 | MMAT | La promoción de estilos de vida saludable aprovechando los espacios públicos | 5.1 Rationale for using mixed methods | Yes | The design explicitly combines environmental observation with a survey to link perceived attributes and well-being, each addresses a different facet of the question. | A sensible two-stage mixed design with clear aims and appropriate measures. Strengths include a grounded description of the setting and use of validated survey scales. Key limitations are integration mostly at interpretation (not analytic), non-probability online sampling (potential selection bias), self-report, and cross-sectional associations. Treat conclusions—e.g., that perceived safety/cleanliness/attractiveness and the program’s multisector nature support well-being—as descriptive and context-specific rather than causal. |
|  |  |  | 5.2 Integration of qualitative & quantitative components | Partial | Components are presented as Estudio 1 (qualitative/observational) and Estudio 2 (survey); integration occurs mainly in the narrative synthesis rather than via formal mixed-methods analysis. |  |
|  |  |  | 5.3 Interpretation of the integrated outputs | Yes | Conclusions jointly interpret that the route provides an attractive, safe, clean environment supporting health/well-being and multisector collaboration. |  |
|  |  |  | 5.4 Handling divergences/inconsistencies | Can’t tell | The paper does not explicitly report or analyze discordant findings between components. |  |
|  |  |  | 5.5 Adherence to quality criteria for each component | Partial / Yes | Qual: systematic field description of route design/operations, but limited detail on qualitative procedures (no coding/audit trail). Quant: uses previously published scales for route attributes, PA, and well-being; demographics and visit patterns collected appropriately. |  |
| 50.0 | MMAT | Logic model of the Recreovía: a community program to promote physical activity in Bogota | 5.1 Rationale for using mixed methods | Yes | Logic-model building benefits from triangulating operational records (quant) with experiential insights (qual) to verify activities, outputs, and assumptions. The paper explicitly follows a convergent approach. | A good mixed-methods design: strong program realism from combining admin metrics with insider perspectives, producing a clear, usable logic model for evaluation and planning. Main limitations are typical for practice-oriented logic-model papers: limited reporting of qualitative rigor and data-quality auditing, and no formal treatment of qual–quant discrepancies. |
|  |  |  | 5.2 Integration of qualitative & quantitative components | Yes | Evidence from interviews and admin records is merged to populate each logic-model box (inputs→activities→outputs→outcomes) and to cross-check volumes (e.g., sites/sessions/users) against staff/budget narratives. |  |
|  |  |  | 5.3 Interpretation of the integrated outputs | Yes | The final logic model and narrative explicitly link resources and activities to short-, medium-, long-term outcomes, interpreting how the combined evidence supports program pathways. |  |
|  |  |  | 5.4 Handling of divergences/inconsistencies | Can’t tell | The paper does not report discordant qual–quant findings or how such discrepancies were resolved (e.g., mismatched user counts vs. staff perceptions). |  |
|  |  |  | 5.5 Adherence to quality criteria for each component | Partial / Yes | Qual: Semi-structured coordinator interviews are appropriate, but reporting on sampling, coding procedures, inter-coder checks, or member-checking is limited. Quant: Administrative counts/budget and site data are clearly tabulated (software noted), though data auditing/validation procedures aren’t detailed. |  |
| 51.0 | AACODS | Criterios técnicos para implementación de una Ciclovía Recreativa | Authority | Yes | Produced by Ministerio de Salud del Perú – Dirección General de Promoción de la Salud; an official government technical guideline/manual with legal deposit and first-edition details. | Treat as grey literature on how Ciclovías should be implemented and measured in Peru. Do not treat it as empirical evidence of impact; instead cite it when describing program standards, required modules, minimum route lengths, frequency, and monitoring procedures. |
|  |  |  | Accuracy | Yes | Specifies purpose, scope, definitions, and procedures; prescribes minimum route lengths (linked to municipal population), recurring weekly operation (typically Sundays, ~4 hours), five required modules (3 physical activity, 1 healthy eating, 1 health promotion), staffing/safety/volunteer roles, and standardized monitoring (participant counts and short surveys) with annexed forms/checklists. Content is internally consistent and operationally detailed, though it is not peer-reviewed research. |  |
|  |  |  | Coverage | Yes | Covers the full implementation cycle: intersectoral coordination, planning and programming, budgeting via Programa Presupuestal 101, execution standards (set-up/tear-down, detours, signage), and final reporting to regional health authorities. Designed for Peruvian municipalities under the 2015 incentive plan (context-specific but comprehensive within that remit). |  |
|  |  |  | Objectivity | Partial | The document’s goal is program promotion and standardization by the health ministry; tone is prescriptive/advocacy-leaning rather than neutral appraisal. Nevertheless, it includes verifiable operational criteria and data-collection protocols (counts/surveys). |  |
|  |  |  | Date | Yes | 2015 publication clearly stated. Useful for historical and methods context; if applying to current policy, check for updated editions or superseding circulars. |  |
|  |  |  | Significance | Yes | Provides nationally standardized criteria that many municipalities likely used to run/measure Ciclovías. Highly relevant to implementation context (what counts as a “compliant” Ciclovía, how participation is counted/surveyed). Not a source of causal effects but directly informs program design and monitoring. |  |
| 52.0 | AACODS | Open Streets for Whom? Toward a Just Livability Revolution | Authority | Yes | Published as a Viewpoint in the Journal of the American Planning Association (peer-reviewed planning journal), which confers strong venue credibility. | "This viewpoint contributes a conceptual/equity framework to interpret who benefits (and who may be excluded) from Open Streets. It is not an empirical effects study, but it provides a robust lens (framework + paradoxes + recommendations) to assess distributional, procedural, recognitional, and interactional justice in program design and policy.     " |
|  |  |  | Accuracy | Yes | The article clearly states its purpose and builds an environmental-justice framework (distributional, interactional, procedural, recognitional) and examines six paradoxes; assertions are grounded in planning/justice literature and recent open/slow streets examples. |  |
|  |  |  | Coverage | Partial | Focuses on conceptual analysis with primarily U.S. examples, while noting ~600 cities worldwide engaged in open/slow/safe streets by Jan 2021; not intended as a systematic or empirical review. |  |
|  |  |  | Objectivity | Partial | Normative stance (justice-oriented planning), yet the piece explicitly surfaces tensions/paradoxes (e.g., displacement, safety, recognition) and proposes policies/programs/partnerships, showing reflexivity rather than one-sided advocacy. |  |
|  |  |  | Date | Yes | 2021, timely to pandemic-era street reallocations; appropriate for contextual/equity framing of recent open-streets practice. |  |
|  |  |  | Significance | Yes | Offers a practice-relevant environmental justice lens and actionable guidance (policies, programs, partnerships) for making open/slow streets more just, useful to interpret implementation choices and potential inequities. |  |
| 53.0 | AMSTAR-2 | From “streets for traffic” to “streets for people”: can street experiments transform urban mobility? | 1. Research question & inclusion criteria include PICO? | No | Paper develops a conceptual framework/typology, not a PICO-framed question with explicit inclusion criteria. | Critically low rating according to AMSTAR-2. This reflects a scope mismatch: the article is valuable as a conceptual framework/typology of street experiments and their potential for system change, not as an effect-size synthesis. |
|  |  |  | 2. Protocol established a priori / deviations justified | No | No protocol/registration described (fits narrative/theoretical piece). |  |
|  |  |  | 3. Rationale for eligible study designs | No | No inclusion criteria by design; it is not a study-effects synthesis. |  |
|  |  |  | 4. Comprehensive literature search | No | No inclusion criteria by design; it is not a study-effects synthesis. |  |
|  |  |  | 5. Study selection in duplicate | No | The paper is a narrative review/discussion; no systematic search methods reported. |  |
|  |  |  | 6. Data extraction in duplicate | No | Not reported; no standardized extraction. |  |
|  |  |  | 7. List of excluded studies with justification | No | Not reported; no standardized extraction. |  |
|  |  |  | 8. Description of included studies in adequate detail | Partial Yes | Provides examples/typology of street experiments and discusses cases, but not a structured study table. |  |
|  |  |  | 9. Risk of bias (RoB) assessment for included studies | No | No RoB appraisal (the goal is framework building, not effect estimation). |  |
|  |  |  | 10. Report sources of funding for included studies | No | No RoB appraisal (the goal is framework building, not effect estimation). |  |
|  |  |  | 11. Appropriate meta-analytic methods (if MA done) |  | No meta-analysis performed (conceptual paper). |  |
|  |  |  | 12. RoB accounted for in meta-analysis/synthesis | No | Not applicable/undertaken. |  |
|  |  |  | 13. Consideration of RoB when interpreting results | No | Interpretation focuses on transition/justice frameworks and acknowledges limited evidence of broader transformation, not RoB. |  |
|  |  |  | 14. Explanation of heterogeneity (if MA) |  | No Quantitative synthesis. |  |
|  |  |  | 15. Investigation of publication bias (if MA) | No | Not applicable/undertaken. |  |
|  |  |  | 16. Report of review authors’ conflicts of interest | Yes | Funding statements and conflicts of interests provided. |  |
| 54.0 | MMAT | Community Benefits and Lessons for Local Engagement in a California Open Streets Event: A Mixed-Methods Assessment of Viva CalleSJ 2018 | 5.1 Rationale for using mixed methods | Yes | The evaluation explicitly combines a broad survey (reach, satisfaction, PA, spending, access) with qualitative interviews/observation to understand how and why benefits occurred and to surface engagement gaps/solutions. | A good, practice-oriented mixed-methods evaluation: strong on breadth (large survey) and context (many interviews + observation), and it delivers clear recommendations for future engagement. Main limitations are non-probability sampling, limited reporting on qualitative rigor/coding, and only partial treatment of quant–qual divergences. Treat findings as credible descriptive evidence with actionable implementation lessons, not as causal estimates. |
|  |  |  | 5.2 Integration of qualitative & quantitative components | Yes | Quantitative findings (e.g., high satisfaction, ≥1 h PA, on-route purchases) are triangulated with interview themes (e.g., generally positive merchant/resident sentiment but advance outreach gaps) and field notes; integration appears in combined results and recommendations. |  |
|  |  |  | 5.3 Interpretation of the integrated outputs | Yes | The report synthesizes strands into actionable guidance (strengthen pre-event engagement, information channels, logistics) grounded in the convergence of survey patterns and qualitative perspectives. |  |
|  |  |  | 5.4 Handling divergences/inconsistencies | Partial / Can’t tell | The narrative acknowledges pockets of concern (e.g., some merchants felt under-informed) alongside overall positive participant/merchant impressions, but it does not systematically analyze discordant cases or quantify where/why results diverged. |  |
|  |  |  | 5.5 Adherence to quality criteria for each component | Yes | Quant: Large on-route convenience sample with clear measures (PA time, spending, mode, satisfaction); sampling limits and potential self-selection are implicit. Qual: Substantial number of stakeholder interviews with described topics and context; methodological details on coding/reliability are limited. |  |
| 55.0 | MMAT | A Survey of Viva CalleSJ Participants:San Jose, California 2016 | 4.1 Sampling strategy relevant? | Yes | Convenience sampling of participants along the 6-mile route during the event; aligns with the goal of describing attendees’ experiences/behaviors that day. | A useful event-day snapshot with clear, straightforward descriptive analysis. Main limitations are representativeness (convenience sample vs. total attendees) and unknown nonresponse, plus standard self-report biases. |
|  |  |  | 4.2 Sample representative of target population? | Can’t tell | Convenience intercepts (n=318) from five sites may not represent all attendees (city estimated ~100k). No weighting or coverage assessment reported. |  |
|  |  |  | 4.3 Measurements appropriate (valid/reliable)? | Yes | Brief questionnaire (English/Spanish/Vietnamese) captured PA minutes, mode, spending, info sources, and demographics, appropriate to aims; all measures are self-reported, with unknown test–retest reliability. |  |
|  |  |  | 4.4 Risk of nonresponse bias low? | Can’t tell | Response rate and refusals not reported for on-route self-completion; self-selection likely among those willing to stop and fill a survey. |  |
|  |  |  | 4.5 Statistical analysis appropriate? | Yes | Descriptive summaries (e.g., ≥60 min PA for ~72% of respondents; top info sources; spending categories) match the non-inferential purpose. |  |
| 56.0 | MMAT | Do open streets initiatives impact local businesses? The case of Sunday Streets in San Francisco, California | 3.1 Are participants representative of the target population? | Can’t tell / Partial | Routes were selected by SFMTA across five neighborhoods; data were collected from businesses along these routes at six events (two Mission, one each Bayview, Chinatown, Western Addition, Excelsior). Representativeness beyond these corridors is unclear. | A good quasi-experimental merchant evaluation with appropriate pre/post vs event measurement and model-based analyses; field procedures (e.g., Tuesday revenue collection) strengthen measurement fidelity. Main limitations are representativeness (route-bounded, agency-selected corridors), incomplete reporting on response/attrition, and limited detail on broader confounder control. |
|  |  |  | 3.2 Are measurements appropriate (valid/reliable)? | Yes | Instruments captured revenue, customer activity, employees, and engagement; collection moved to Tuesday mornings so merchants could compile accurate revenue totals from the prior Sunday. Measures are appropriate but self-reported. |  |
|  |  |  | 3.3 Are outcome data complete? | Can’t tell / Partial | Analytic models report n≈333 observations (e.g., Table 8), but the paper notes practical field constraints (busy shops, variable closing times). A consolidated response/attrition profile across pre/post vs event waves isn’t fully detailed. |  |
|  |  |  | 3.4 Are confounders accounted for in design/analysis? | Partial Yes | Authors use regression (e.g., multinomial logit in Table 8) including merchant engagement level and business type. Broader controls (e.g., route/time fixed effects) are not thoroughly described in the excerpts available. |  |
|  |  |  | 3.5 Did the exposure occur as intended? | Yes | Event exposure was tied to specific Sunday Streets dates on selected routes; baseline measures were taken two weeks before and two weeks after non-event Sundays per protocol. |  |
| 57.0 | MMAT | Mapping Equality in Access: The Case of Bogotá's Sustainable Transportation Initiatives | 4.1 Sampling strategy relevant? | Yes | Not a sample of people; a full-population spatial analysis of the city networks and SES blocks, appropriate for a descriptive access question. | A strong descriptive GIS study: citywide coverage, transparent access metrics, and relevant equity scenarios. Main limitations are inherent to potential access proxies (not behavior), cross-sectional design, and minor temporal misalignment between network and SES data. |
|  |  |  | 4.2 Sample representative of target population? | Yes | Coverage is citywide for the study years, so representative of Bogotá’s built networks/SES then; caveat: temporal mismatch (2009 networks vs 2005 SES) and networks evolve. |  |
|  |  |  | 4.3 Measurements appropriate (valid/reliable)? | Yes | Uses walking-time buffers for TransMilenio and distance buffers for Cicloruta/Ciclovía, standard proxies for potential spatial access (not actual use). Thresholds and assumed walking speed are stated. |  |
|  |  |  | 4.4 Risk of nonresponse bias low? |  | No surveys; analysis is based on administrative/GIS layers and census data. |  |
|  |  |  | 4.5 Statistical analysis appropriate? | Yes | Descriptive SES-by-access comparisons and scenario mapping are aligned with aims; results are presented as distributions/percentages and interpreted against known usage patterns. |  |
| 58.0 | AACODS | The Open Streets Guide | Authority | Yes | Produced by The Street Plans Collaborative with the Alliance for Biking & Walking and OpenPlans (credits listed), credible sector organizations. | Treat the Guide as authoritative grey literature for how programs are organized and implemented (typologies, logistics, examples). Because it’s 2012, pair it with newer syntheses or local documents when making current claims, but keep it for definitions, models, and historical context. |
|  |  |  | Accuracy | Yes | States project scope and methods (web/print scan, phone/email interviews, 10-question online survey), and compiles 67 case studies organized into seven model types with a companion site for updates. Methods and deliverables are explicit. |  |
|  |  |  | Coverage | Yes | States project scope and methods (web/print scan, phone/email interviews, 10-question online survey), and compiles 67 case studies organized into seven model types with a companion site for updates. Methods and deliverables are explicit. |  |
|  |  |  | Objectivity | Partial | Focuses on North American initiatives; includes overview, best practices, typology, summary graphics, and a city map showing growth (2005–2011). Provides an appendix with resources and a full bibliography. |  |
|  |  |  | Date | Yes | Volume 1 — February 2012 is clearly stated; the guide notes the companion website for continuing updates, signaling content may evolve. |  |
|  |  |  | Significance | Yes | Early, widely-cited compilation and typology that documents program models, organization/funding patterns, and route settings, useful for designing and comparing initiatives. |  |
| 59.0 | MMAT | Influences of Built Environments on Walking and Cycling: Lessons from Bogota | 3.1 Are participants representative of the target population? | Yes | Probability sampling across 30 neighborhoods with block–household selection supports representativeness of urban residents in sampled areas. | A strong cross-sectional analytic study: probability-based household sampling, objective GIS exposures, validated PA instrument, and multilevel modeling. Main limitations are cross-sectional design (no causality), self-reported PA, and possible limits to citywide representativeness beyond sampled neighborhoods. |
|  |  |  | 3.2 Are measurements appropriate (valid/reliable)? | Yes | Transport PA from IPAQ-long (Spanish, adapted) with accelerometer validation (ρ≈0.42; test–retest r≈0.69); built-environment exposures from GIS (street density/connectivity, proximity to TransMilenio, etc.), fit the aims. |  |
|  |  |  | 3.3 Are outcome data complete? | Yes | Analytic sample reported for the full survey (≈1,500); no major missing-data losses described. |  |
|  |  |  | 3.4 Are confounders accounted for in design/analysis? | Yes | Multilevel logistic regressions used; models include covariates (socio-demographics, neighborhood factors) when estimating associations with walking/cycling. |  |
|  |  |  | 3.5 Did the exposure occur as intended? | Yes | Exposures are objective GIS metrics (street network, transit proximity), and outcomes follow IPAQ protocol; acknowledge typical self-report bias for PA. |  |

**1.7 Supplementary Table 7.** ERIC strategies not relevant to Open Streets Strategies

| ERIC strategies are not relevant to Open Street Strategies | |
| --- | --- |
| 1 | Change accreditation or membership requirements |
| 2 | Change liability laws |
| 3 | Change Record Systems |
| 4 | Conduct cyclical small tests of change |
| 5 | Create new clinical teams |
| 6 | Develop an implementation glossary |
| 7 | Develop disincentives |
| 8 | Make billing easier |
| 9 | Organize clinician implementation team meetings |
| 10 | Place innovation on fee for service lists/formularies |
| 11 | Provide ongoing consultation |
| 12 | Remind clinicians |
| 13 | Revise professional roles |
| 14 | Shadow Professional roles |
| 15 | Use capitated payments |
| 16 | Use data warehousing techniques |
| 17 | Use train-the-trainer strategies |
| 18 | Visit other sites |
| 19 | Provide clinical supervision |
| 20 | Model and simulate change |
| 21 | Obtain and use patients and family feedback |

**1.8 Supplementary Table 8.** List of Open Streets Strategies

| **List of Open Streets Strategies** | | **Ref.** |
| --- | --- | --- |
| 1 | Apply tactical urbanism* to Open Street programs to promote mobility in times of health emergencies. | [18] |
| 2 | Encouraging communication between different parties promotes engagement as a common goal of PA promotion. | [34] |
| 3 | Utilize family cohesion to motivate children to participate in Open Streets. | [35] |
| 4 | Motivate participants by promoting social interactions. | [36] |
| 5 | PA instructors play an important role in PA promotion through social media. | [37] |
| 6 | Active transportation can be socially transmitted and become a norm to promote PA in Open Streets programs. | [38] |
| 7 | The program's evaluation strategy involves studying the distance between Open Streets and socioeconomic status (SES) categorized areas. | [39] |
| 8 | Allow flexibility of the program to adjust to changes in budget and increase participation. | [42] |
| 9 | Encouraging capacity building to increase the quality of the program. | [42] |
| 10 | Budget allocation and accountability to improve and scale up the programs. | [42] |
| 11 | Identify champions. | [50] |
| 12 | A thorough evaluation of programs and identification of barriers, facilitators, and outcomes are key for sustainability. | [47] |
| 13 | Consider the length and frequency of the program, which can influence participation. | [52] |
| 14 | Direct communication and marketing efforts toward city residents who experience health disparities. | [54] |
| 15 | Incorporate common evaluation metrics to support future open street evaluations, such as motivation to host and cost/staffing requirements to maintain the initiative. | [55] |
| 16 | Utilizing indoor and outdoor settings as alternatives to the Open Street program for older adults during public health emergencies. | [56] |
| 17 | Increasing open street programs’ reach, capacity, and frequency to lower costs. | [57] |
| 18 | A small-scale open street event can be easily disseminated and feasible for long-term physical activity participation. | [58] |
| 19 | Gather and foster local political support and collaboration. | [61] |
| 20 | Community PA programs must engage with multiple sectors for widespread integration. | [60] |
| 21 | Incorporate open street initiatives in broader citywide agendas. | [61] |
| 22 | Partner with academic institutions or organizations that regularly implement evaluations to assess open streets. | [61] |
| 23 | Programs must reduce and expedite permitting requirements. | [62] |
| 24 | New programs should consider different funding mechanisms for costs related to traffic control (such as policing, firefighters, and EMS). | [62] |
| 25 | Open street programs should build a consistent brand and visibility to provide identity and encourage repeat participation and sponsorship. | [62] |
| 26 | Foster collaboration between international networks and multidisciplinary groups. | [63] |
| 27 | Selecting and targeting populations benefitting from the program is an optimal strategy. | [64] |
| 28 | Increase individuals' participation by creating buddy systems, educational activities, and policy and environmental changes. | [64] |
| 29 | With political support, other countries can benefit from road closures and existing infrastructure to promote PA. | [67] |
| 30 | Community-based participatory research and including a more diverse population in outreach can increase the participation of the under-resourced. | [68] |
| 31 | Plan for sustainability and funding by engaging businesses. | [68] |
| 32 | PA promotion is underlined with free classes; funding is important for sustainability. | [69] |
| 33 | PA promotion can be sustainable by fostering good relationships, culturally sensitive programs, and the right staff. | [70] |
| 34 | Community groups are responsible for Play Streets' operation to engage population’s PA. | [72] |
| 35 | A diversity of entities and stakeholders, such as policymakers, health insurance companies, funders, businesses, and communities, are required to sustain the program. | [73] |
| 36 | Involve community partners, merchants, residents, and city agencies in the implementation process. | [74] |
| 37 | Promote the intervention through media. | [74] |
| 38 | Consider context to develop culturally appropriate approaches. | [75] |
| 39 | Increase awareness among the population about using the structure available for Open Street programs. | [77] |
| 40 | Strengthen alliances with the public and private sectors. | [80] |
| 41 | Multi-sectoral collaboration to encourage co-benefits of the program. | [65] |
| 42 | Implement health education sessions for physical activity, nutrition, and wellness. | [81] |
| 43 | Ensure widespread dissemination of the open street program and its benefits. | [79] |
| 44 | Utilize public health representatives to stress the program's importance and justification for health and physical activity promotion. | [81] |
| 45 | Use of static publicity of the programs in widely used spaces. | [81] |
| 46 | Introduce anti-displacement policies and strategies. | [82] |
| 47 | Create a policy to site open streets near schools/ community centers. | [82] |
| 48 | Explore and enact community-based alternatives to policing. | [82] |
| 49 | Provide technical assistance to BIPOC businesses in adapting their strategies. | [82] |
| 50 | Build partnerships within the environmental justice community. | [82] |
| 51 | Understand and leverage community power. | [82] |
| 52 | Build an anti-racist planning culture from within. | [82] |
| 53 | Encourage agenda setting for open streets by BIPOC residents. | [82] |
| 54 | Ensure targeted outreach to businesses and community-based organizations to increase participation. | [84] |
| 55 | Encourage people to market the open street event to peers through multiple channels. | [85] |
| 56 | Utilize entertainment, food trucks, and resource tables to attract participants. | [85] |
| 57 | Encourage merchants and businesses to support the community goal of open street programs. | [77] |
| 58 | Open street organizers should undertake several concurrent political and logistical planning efforts that successfully move the proposal from concept to implementation. | [87] |
| 59 | Open street organizers should build a willing coalition of advocacy, municipal, and/or private-sector supporters. | [87] |
| 60 | Establishing political support from the mayor, city council, and/or other political representatives is important because elected officials most commonly allocate the public resources needed for implementation. | [87] |
| 61 | Once government and political buy-in is obtained, the municipal leaders should assign the appropriate department to organize the initiative or serve as the liaison between the city’s dedicated resources and the lead organizing entity. | [87] |
| 62 | Acquire Municipal Funding and/or Significant In-Kind Support from Public authorities should lead in dedicating public funds and/or resources. | [87] |
| 63 | Organizers should critically evaluate and share the successes and failures to improve the next effort. | [87] |
| ***Tactical Urbanism**: an approach involving short-term, low-cost, and scalable interventions to improve public spaces and utilize underutilized areas. | | |
